# The growing repertoire of putative pathogen interaction genes in the *Biomphalaria glabrata* vs *Schistosoma mansoni* system

**DOI:** 10.1371/journal.pntd.0014803

**Published:** 2026-10-21

**Authors:** Javier Calvelo, Stephanie R. Bollmann, Jacob A. Tennessen, Michael S. Blouin

**Affiliations:** 1 Department of Integrative Biology, Oregon State University, Corvallis, Oregon, United States of America; 2 Immunology and Infectious Diseases, Harvard T.H. Chan School of Public Health, Boston, Massachusetts, United States of America; Universite de Perpignan, FRANCE

## Abstract

Schistosomiasis is major neglected tropical disease of humans that is caused by trematodes in the genus *Schistosoma*. Humans are infected during contact with water in which schistosomes are being shed from their intermediate hosts, freshwater snails. Snail control is effective at reducing transmission, but can be expensive and ecologically problematic. Therefore, there is substantial interest in understanding the genetic mechanisms behind variation in snail resistance to infection by schistosomes, with the goal of leveraging that information to reduce transmission at the snail stage. *Biomphalaria glabrata* is an important host snail for transmission of *S. mansoni* in the New World. Here we reanalyzed an older Genome-Wide Association Study (GWAS) dataset from *Biomphalaria glabrata* snails challenged by the trematode, *Schistosoma mansoni*. We identified two new regions of association with resistance to infection, named LG5R and LG11L. LG11L contains a cluster of genes for a diverse set of secreted lectins, which could be involved in some type of recognition. LG5R contains a cluster of genes for what appear to be membrane-bound receptors. These genes are very similar to those identified in an adjacent genomic region by a recent GWAS on the African snail, *B. sudanica* challenged by *S. mansoni*. Clusters of genes for single-pass transmembrane proteins also characterize two other genomic regions of association with resistance to *S. mansoni* that were previously described for *B. glabrata* (the PTC1/GRC and PTC2 regions). We hypothesize that the proteins coded for in such clusters of genes are involved in some type of matching polymorphism that may explain the ubiquitous parasite-population by snail-population interaction in compatibility that characterizes the *B. glabrata-S. mansoni* system. We discuss our results in light of the state of the field of genome-wide mapping studies on *Biomphalaria sp.*, and discuss what types of loci found to date would be the best targets for reducing transmission in natural populations.

## Author summary

Schistosomiasis is a major neglected tropical disease of humans that is caused by trematodes in the genus *Schistosoma*. Schistosomes require aquatic snails as obligate intermediate hosts. Humans become infected by contact with water in which snails are shedding parasites. Recent efforts have focused on understanding the genetic basis of variation in resistance of snail hosts to infection by the parasite. In this work, we characterized two new genomic regions in the host snail, *Biomphalaria glabrata*, at which genetic variation controls resistance to infection by *S. mansoni*. One region contains a cluster of genes for membrane-bound receptors, while the other contains a diverse array of genes for secreted lectins. We hypothesize that genes in both regions are involved in recognition of the parasite. We compare these results to those of other mapping studies that have turned up other clusters of genes that appear to have similar functions. We discuss what mapping studies to date have revealed about the genetic basis of resistance in snails and consider the types of loci that would be the best targets for making snails resistant to infection by schistosomes.

## Introduction

Schistosomiasis is an important neglected tropical disease whose transmission requires aquatic snails as the intermediate host [1]. There are no effective vaccines, and treatment relies on a single drug, praziquantel [2]. Mass drug administration alone has proven ineffective at eliminating the disease and could select for drug resistance [3–6]. Eliminating snail hosts using molluscicides or habitat alteration can be very effective at reducing transmission [6,7]. However, snail population control is often impractical or entails unacceptably high economic and ecological costs [8,9]. Therefore, alternative approaches that target the snail stage would be very useful. Genetic modification of snail populations to make them less able to transmit the parasite is a plausible approach [10–13]. However, we first need to understand the genetic architecture of resistance, characterize the functions of key genes, and assess their variation and fitness effects in natural populations.

Genome-wide scans and candidate gene approaches have identified at least nine genomic regions in two snail species at which allelic variation influences resistance to schistosomes (see Zhang et al., 2025 and Table 2 in Blouin et al. [14], for description of seven regions in *Biomphalaria glabrata*, and Pennance et al. [15], for description of two regions in *B. sudanica*). To date, functional studies have implicated actual genes in only two of those regions (*Cu-Zn sod1*, which is involved in the oxidative burst [16,17] and *grctm6*, which codes for a putative membrane-bound receptor [18]). Nevertheless, a recurring pattern under many of the peaks of association identified so far is that the regions tend to be highly variable in both DNA sequence and chromosome structure, and to be enriched for what appear to be clusters of genes for membrane-bound receptors [14,15,19,20]. Furthermore, different genomic regions stand out as significant in studies using different combinations of snail and parasite populations [21–24] It has thus been suggested that these receptor loci are involved in some type of matching alleles or gene-for-gene interaction [14,15,25]. Such loci might not be ideal targets for genetic modification if allelic effects are specific to particular schistosome genotypes, rather than being universal. Therefore, studies using a variety of populations of snail and parasites are needed to better understand how the immune system of snails works, and to determine which loci might be the best targets for universal application.

*Schistosoma mansoni* and its neotropical host, *Biomphalaria glabrata*, are the most well studied model for snail-schistosome compatibility. In a previous study, Tennessen et al. [20] used pool-seq GWAS on snails from the 1316R population of *B. glabrata* to map loci that control their susceptibility to infection by PR1-population *S. mansoni*. A sliding-window Fst analysis revealed a very strong peak that we described as the PTC2 region. We also observed minor peaks such as those surrounding the *Cu-Zn sod1* gene and the *RADres1* marker locus, which we had previously studied in the 1316R population [16,17,26,27] (hereafter referred to as the SOD and RADres regions). In that study and subsequent studies, we focused our attention on the PTC2 region (e.g., [14,28]) because the signal strength suggested it contained causal genes that disproportionally contributed to the infection outcome in that infection challenge. Nevertheless, the genes located in the regions with a lower effect are still components of the snail immune system that merit analysis.

In Tennessen et al. [20] we had mapped Illumina reads to a PacBio assembly created in 2017 from inbred line i68, which was derived from a 1316R-population snail. Although the i68 assembly was the best genome available for *B. glabrata* at the time, it was highly fragmented (e.g., contig N50 = 743 Kb). Although we had pulled most of the i68 contigs into linkage groups using sequence capture markers [27], 22% of the genome could not be assigned to a linkage group. Thus, it is possible that we missed some regions of association if relevant contigs were in the “unassigned” pile. Furthermore, within each linkage group we could not reliably order many of the contigs. The peaks around the SOD and RADres regions appeared to be very wide, encompassing much of their respective linkage groups (Fig 1 in Tennessen et al. [20]). In the case of the SOD region, this was not surprising, as we already knew it to be a large (~10 Mb) block of high linkage disequilibrium (LD) [27]. In the case of the *RADres* locus, it appeared there might be multiple distinct peaks on its linkage group. But because we could not properly order the contigs in that linkage group, we could not rule out that it was just one large block of LD, as in the case of SOD.

**Fig 1:**
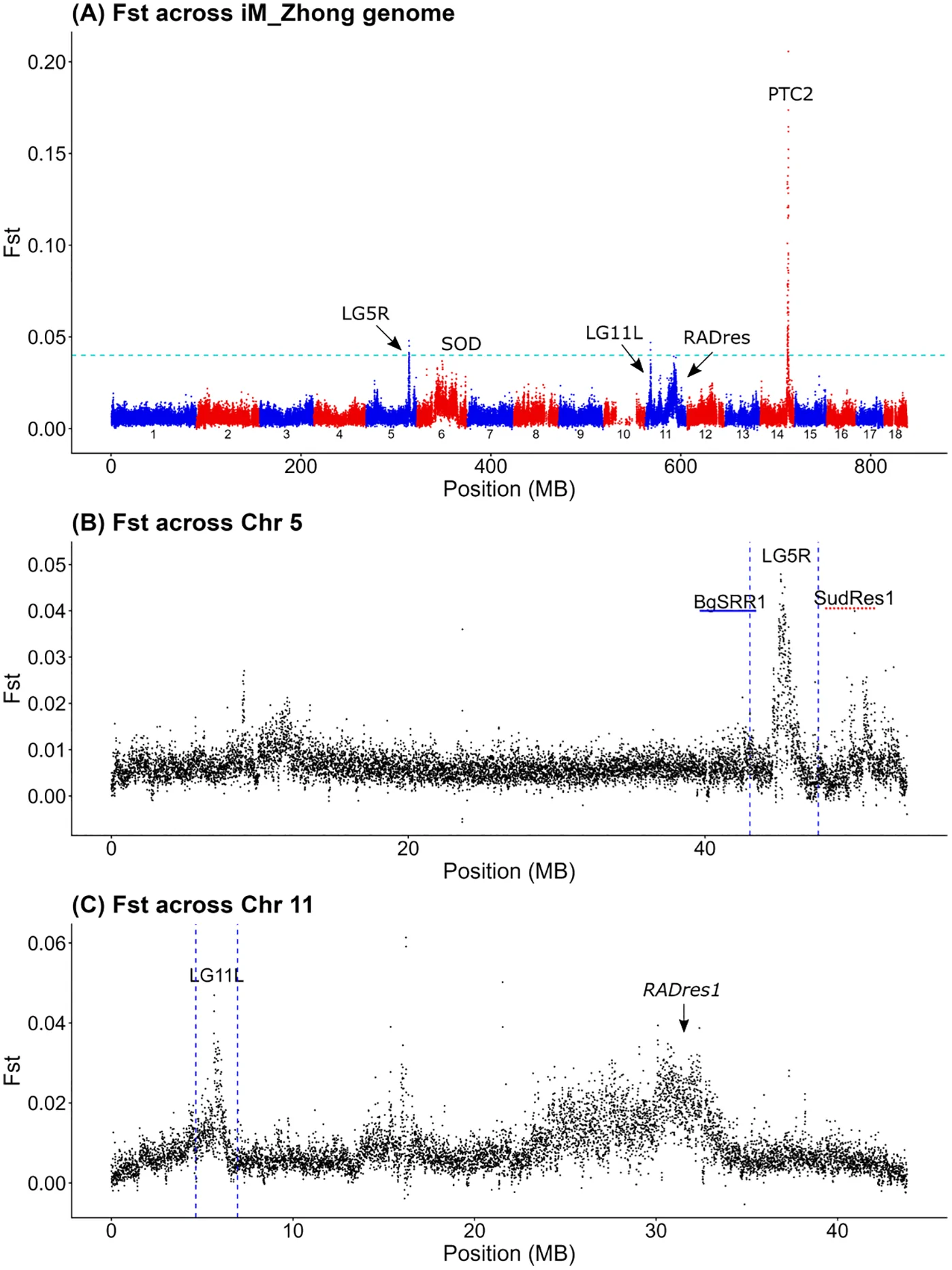
Sliding window Fst analysis (10 Kb windows), with reads aligned to the iM_Zhong reference assembly. **(A)** Reads aligned to the entire genome, plotting only windows having at least 150 SNPs per window. Dotted line is the Fst = 0.04 cutoff. The two new regions are identified as LG5R and LG11L. The SOD, RADres and PTC2 regions were identified in previous studies. **(B)** Sliding window Fst across chromosome 5 only, using windows having at least 5 SNPs per window. Blue bar = location of the BgSRR1 region from Zhang et al. [29], Dotted Red Bar = approximate location of SudRes1 [30]. **(C)** Sliding window Fst across chromosome 11 only, using windows having at least 5 SNPs per window. Arrow = original *RADres1* marker locus scored in Tennessen, Bonner, et al., (2015). We did not call the diffuse peak in the middle of chromosome 11 because it is a region of extreme structural variation that also shows elevated Fst in the 50:50 mixture comparisons (S1 File). The dashed blue lines on panel B and C mark the analyzed regions for LG5R (CM074206.1: 43,022,826 to 47,628,293) and LG11L (CM074212.1: 4,648,498 to 6,946,461) respectively.

There now exist much higher-quality genome assemblies for *B. glabrata* [31–34]. So here we mapped the Illumina reads from Tennessen et al. [20] onto Zhong et al. [32] chromosome-level assembly, and repeated the sliding window Fst analysis. Our goal was to determine firstly, if there were any minor peaks we missed the first time, and secondly, whether the RADres region on chromosome 11 is one large block of LD, or whether there are multiple independent peaks. We identify a new peak on chromosome 5 that we missed the first time, and we determine that chromosome 11 has two distinct peaks on it, one of which contains the original *RADres* marker locus. We describe each of the new peaks of association and speculate about which protein-coding loci therein are the most compelling candidates to explain the variation in susceptibility. We also reannotate the loci under the PTC1 (originally named GRC) and PTC2 regions [20,26] and comment on similarities among loci in those regions and those in our new regions. We end by discussing our results in light of what has been learned from other genome-wide mapping studies to date, including which types of loci would be the best targets for genetic manipulation of host snails in the field.

## Methods

### Origins of the study populations discussed in this manuscript

The 1316R GWAS population has been in culture at Oregon State University since the late 1970s, first in Christopher Bayne’s lab, now in Michael Blouin’s. It was developed at the NIH by Richards and colleagues in the 1970s when they were experimenting with crosses involving Brazilian and Caribbean isolates of *B. glabrata* (see Fig 1 in Richards & Shade [21], and references therein). The original M-line population from NIH was developed in the 1950s via a cross between an albino Brazilian snail and a pigmented Puerto Rican snail, followed by selection for albinism and susceptibility to a Puerto Rican strain of *S. mansoni*, PR1 [35,36]. 1316R is very likely descended from the M-line population, as both have the albino phenotype (and C. Bayne, pers. Comm.). The BS90 population was reportedly isolated in Salvador, Bahia, Brazil, in the 1960s, and has been in labs in the US since the 1990s [37], cited in Knight et al. [38]; E.S. Loker, pers. comm.). All BS90 are completely resistant to PR1-population *S. mansoni*, while M-line and 1316R are of high and low/medium susceptibility, respectively. Populations of M-line and BS90 have been maintained and distributed by the Biomedical Research Institute (BRI) schistosomiasis resource center (https://www.afbr-bri.org/schistosomiasis/) for decades. The source of the Berriman & Buddenborg [33] genome assembly (hereafter the ‘Bg2024 population’) appears to be from a M-line population maintained in the United Kingdom since 1962 [33].

### Reanalysis of the Tennessen et al. (2020) GWAS

Tennessen et al. [20] used 600 infected and 600 non-infected snails from the 1316R population of *B. glabrata* as cases and controls in a pool-seq GWAS to find loci that control susceptibility to the PR1 population of *S. mansoni*. Each infection group consisted of two, independent pools of 300 individuals (incorrectly referred to as technical replicates in the original manuscript). They created an individually-barcoded library for each pool of 300, and then sequenced all four libraries via 151-bp paired-end reads across six lanes of an Illumina HiSeq 3000 (NCBI BioProject Accession PRJNA638474). Here we re-mapped those Illumina reads to the iM line genome assembly of Zhong et al. [32] (hereafter reference ‘iM_Zhong’). We then compared the infected and noninfected groups across the entire genome via sliding-window Fst analysis as in Tennessen et al. [20] (10 Kb windows, moving 5 Kb each interval). For calling Fst peaks across the entire genome, we used a conservative approach of plotting only windows that had a minimum of 150 SNPs per window, and ignoring stray points that were not part of a well-defined peak. Having identified peaks of interest on chromosomes 5 and 11, we plotted Fst across those two chromosomes using a more liberal criterion of minimum 5 peaks per window to better describe the boundaries of each peak.

### Identifying significant peaks in the GWAS

Given the pool-seq study design, it is difficult to assign P-values to individual Fst peaks. However, the peaks around the known loci, SOD and RADres (Fst ~ 0.04) stood out as well above the background in the original analysis (Fig 1 in Tennessen et al. [20]). So, any new peaks that are as high or higher than the peaks for SOD or RADres are likely to be real. Secondly, from simulations in Tennessen et al. [20] we estimated that in comparing two pools of N = 600 each, Fst is unlikely to exceed 0.04 by chance (P < 0.0001; S1 File). Thirdly, when we did the original pool-seq GWAS we used two individually-barcoded libraries of N = 300 snails each for each group (infected or non-infected). That allows us to bioinformatically mix libraries and do a sliding window Fst analysis between two artificial pools of N = 600 each that are both a 50:50 mix of infected and uninfected snails. Fst peaks in that comparison can’t have anything to do with resistance/susceptibility and so tell us how high random peaks are likely to get. In that analysis only one Fst window exceeded Fst = 0.03, and no well-defined peaks (supported by multiple adjacent windows) exceeded Fst ~ 0.025 (S1 File). So, Fst > ~0.04 appears to be a conservative cutoff for identifying significant peaks in the reanalysis.

We identified a new peak on chromosome 5 and two peaks on chromosome 11, one of which contains the original *RADres1* locus from Tennessen, Bonner, et al. [19]. We named the regions under these three peaks LG5R, LG11L, and RADres. We validated the two new peaks (LG5R, and LG11L) using PCR on an independent sample of 430 challenged snails from the outbred 1316R population (PCR and marker locus details in S1 Table), and tested for association via binary logistic regression (using SYSTAT 13 [39]). These validation snails were originally used in [Tennessen et al. [19], and were challenged using the same protocols as the GWAS snails (5 miracidia per snail, checked for shedding weekly between weeks 5–10 after challenge). We still have tissue from the original 1200 GWAS snails, so we also individually genotyped them for technical validation, and to describe patterns of dominance at each locus.

### 1316R genome assemblies used in this study

Larson et al. [40] developed 52 inbred lines from the 1316R population, of which we chose four to create PacBio assemblies in the late 2010s. Three of these assemblies, i68, i90 and i171, were used in Tennessen et al. [20] (representing PTC2 alleles *R*, *S1* and *S2*, respectively). A fourth, lower-quality assembly (i36) remained unpublished. After identifying the new regions of association in this study, we identified resistant (R) and susceptible (S) haplotypes at each region. To do this we first examined the lead windows in each peak (those having highest average Fst) and identified which alleles at each SNP in those windows were in higher frequency in the uninfected group, and which alleles were in higher frequency in the infected group. This defined short haplotypes that are associated with resistance (R) versus susceptibility (S) (S2 Table). We also defined short haplotypes associated with R and S at the right and left edges of each Fst peak (S2 Table). We then determined which haplotype each of our 1316R PacBio assemblies carries at each region and genotyped our remaining 1316R inbred lines to determine their genotypes. We then obtained two additional PacBio assemblies for this study to ensure we have at least two good scaffolds of each haplotype at each region to better distinguish actual allelic variation between R and S haplotypes from sequencing errors (Table 1). These included a snail from line i46, plus another snail from line i36 to improve upon the original i36 assembly (hereafter ‘i36b’). For each PacBio assembly a single adult snail was selected, and genomic DNA was extracted from the headfoot using the Qiagen Blood and Tissue kit. We had to genotype i36 snails to select a homozygous snail for the regions of interest because the i36 line had been contaminated with another susceptible inbred line since the first i36 PacBio assembly was created. Libraries were produced with barcodes using the PacBio SMRTbell Template Preparation kit and sequenced on a PacBio Revio 25M unit across 3 SMRT cells. Genomes were assembled using hifiasm v0.25.0-r726 [41–43] with the “-primary” flag option. PacBio assemblies for i36b and i46 are available at NCBI BioProject Accession PRJNA1463873.

**Table 1:** Haplotype of each 1316R assembly in each new region of association. R = matches resistant haplotype (more common in the uninfected pool), S = matches susceptible haplotype (more common in the infected pool).

Assembly	LG5R	LG11L
i36b	S	S
i90	S	R
i171	S	R
i46	R	S
i68	R	R

### Comparison between 1316R assemblies and reference genome assemblies

The three best *B. glabrata* genome assemblies currently available are the iBS90 assembly from Bu et al. [31], the iM line assembly published in Zhong et al. [32] (an improved version of the iM line assembly presented in Bu et al. [31]), and the Bg2024 assembly from the Sanger Institute [33,34] (hereafter, the three ‘reference genomes’; summarized in Table 2). We used these reference assemblies for two main purposes: (1) to scaffold the older 1316R assemblies, and (2) for annotation transfer. In a few cases when part of a 1316R haplotype was not a good match to any of the three reference assemblies, we checked, on an *ad hoc* basis, whether that section matched any of three additional BS90 assemblies that are also available [14,28].

**Table 2:** *B. glabrata* genome assemblies referenced in this manuscript.

Assembly nickname^1^ (Genbank ID #)	Comments on source population	N50^2^	References
From populations putatively descended from the original M-line population			
iM_Zhong (GCA_025434175.2)	Isogenic line derived from the M-line population in Loker lab (originally from Bayne lab, Oregon State University in 1980s).	47.4Mb	[32]
iM_Bu (GCA_025434175.1)	Earlier version of iM_Zhong	22.7Mb	[31]
Bg2024 (GCA_947242115.1)	Outbred snail. Population obtained from NIH in 1962 and maintained in UK ever since (called NMRI in 2026 update).	48.5Mb	[33,34]
i68 (GCA_014525025.1) i90 (GCA_014524965.1) i171 (GCA_014524955.1) i36b (GCA_060697395.1) i46 (GCA_060697335.1)	Inbred lines (3 generations of selfing) derived from the outbred 1316R population in Blouin/Bayne lab (originally from NIH).	0.7Mb, 2.6Mb, 0.4Mb, 36.4Mb, 22.5Mb	[19,20,40] and this study
*From populations descended from the original BS90 population*			
iBS90_Bu (GCA_025434165.1)	Highly inbred line recently derived from the outbred BS90 population in Loker lab (origin = Lobato Paraense lab, Fundação Oswaldo Cruz, Brazil in 1990s).	19.4Mb	[31]
BS90_FSS5 (GCA_024586215.1)	Inbred line (2 generations of selfing) derived from the outbred BS90 population in Blouin lab (origin = TJC Anderson lab, Texas Biomedical Research Institute, in 2017).	2.3Mb	[14,28]

^1^Not the names used in the original publications. We assigned new names for clarity of discussion.

^2^listed in same order as the assembly names in first column.

Because the original 1316R assemblies were highly fragmented, we first scaffolded each of the original 1316R assemblies on to each of the three best reference genomes (iBS90_Bu, iM_Zhong and Bg2024) using RagTag v2.1.0 [44]. This greatly improved the quality of the old 1316R assemblies, with over 96% of their bases now assigned to a scaffold (S3 Table). Hereafter, when we refer to a 1316R assembly we mean these reference-guided assemblies or the new ones, not the original, highly fragmented versions. 1316R assemblies were then annotated by using Liftoff v1.6.3 [45] to transfer annotations from the three best reference genomes, plus iM_Bu (the earlier version of iM_Zhong from Bu et al. [31]). We added iM_Bu because it differed in annotation from the updated assembly in Zhong et al. [32]. We also transferred annotations among the three reference assemblies. Transfer success statistics are in S1 Table. The annotated 1316R assemblies used in this study and their genome assembly quality metrics are provided in S3 Table.

Large scale alterations of homologous regions of association and surrounding sequence between assemblies were evaluated through dotplot analysis using blastn2dotplots v1.1.2 [46] based on blastn searches [47].

### Gene model definitions

A quick visual inspection of the annotation tracks quickly revealed a gene fragmentation problem with several of the reference genome annotations. This usually manifested when exons from a complete gene in one reference annotation (usually Bg2024) were identified as individual genes in the others. To mitigate the issue, we opted to keep all annotation transfers independent and then we manually grouped gene predictions in each region based on exon overlaps. These “Gene models” were annotated based on protein domains identified with InterProScan [48], Signal Peptides (SP) identified with Signalp6 [49], Mitochondrial Transit Peptide (mTP) with TargetP [50], transmembrane alpha-helices with TMHMM2.0 [51] and KO orthologues identified with KofamKOALA [52]. In a few instances, gene models were found within the introns of other gene models, likely non-coding RNAs (both reported as such or as short putative proteins). These are reported as Gene Model Complexes, identified by the larger Gene Model. Cases where inconsistencies in the Gene Model relative location between assemblies are documented in S2 File.

For each of the two new regions of association, the location of genes on each 1316R assembly was confirmed manually based on synteny conservation with the reference genomes. For each region we also identified which reference was the closest match to each 1316R assembly based on gene synteny and percent identity within those genes. It is worth recalling that while BS90 is a purely Brazilian population, the 1316R, Bg2024 and M-line populations are all related and of Brazilian x Caribbean hybrid origin. Given the hybrid ancestry and many generations of recombination that have ensued since establishment of the 1316R, M-line and Bg2024 populations, it is not surprising that for any region of the genome, our 1316R assemblies often differ from each other and are a best match to different reference assemblies.

### Allelic variation between homologues on the S and R haplotypes

To further identify the most compelling candidate genes in each region of association, we also compared alleles of homologous loci between the R and S haplotypes of the 1316R assemblies to identify putatively function-altering substitutions (non-synonymous substitutions, premature stop codons, splice site disruptions). This was carried out by mapping the 1316R assemblies to the reference assembly that best represented one of the haplotypes with Minimap2 v2.30-r1287 [53]. The expected impact of each variant was predicted with SnpEff v5.4a [54]. See S4 File for details on variant filters and count criteria.

### Genome-wide density of single-pass transmembrane (TM1) genes

Because the PTC2 region appears to have an unusually high density of single-pass transmembrane (TM1) genes [20,28], we examined whether the same was true for our two new regions of association. We used TMHMM v.2.0 to identify genes across the iM_Zhong assembly that have a single-pass transmembrane structure, and plotted their density in 2 Mb sliding windows (sliding 0.5 Mb at a time) on the iM_Zhong assembly. Because annotation differs among the reference assemblies, we repeated this analysis using the annotations transferred from the three other reference assemblies (iM_Bu, iBS90_Bu and Bg2024), which all gave similar results.

### Orthology analysis with SudRes1 in *B. sudanica*

Gene annotation on LG5R suggested a link with the recently described region SudRes1 in *B. sudanica* [30]. To explore the relationship, we conducted a reciprocal blast search between *Biomphalaria sudanica* (assembly Bs111) vs Bg2024 proteins and then cross-referenced their location in the iM_Zhong assembly. Only blast hits with 40% identity and 70% query coverage were considered for the reciprocal blast. To avoid potential partial annotations, genes transferred to the iM_Zhong genome with less than 80% coverage by Liftoff were discarded.

### Code availability

BASH and R scripts utilized in this work are available at: https://github.com/J-Calvelo/Bglabrata_LG5R_and_LG11L.git

## Results

### Reanalysis of the 2020 GWAS data reveals two new peaks

Sliding window Fst analysis of the 2020 GWAS reads mapped to the iM_Zhong reference assembly revealed two new peaks, LG5R and LG11L, that were not apparent in the original analysis (Fig 1). The RADres and SOD regions show broad Fst peaks that cover most of chromosomes 11 and 6 respectively, and therefore a detailed analysis of potential causal genes is not practical. Below we focus on gene content in just the two new regions: LG5R and LG11L.

### Region of association LG5R

On Chromosome 5 there is a clear, strong peak (highest windows mean Fst ~ 0.05) that we named LG5R for its relative position on chromosome (linkage group) 5 (Fig 1B). We missed this region in 2020 because it maps to contigs that were not assigned to a linkage group in our original i68 assembly. LG5R is over 2.5 Mb downstream of the peak identified by Zhang et al. [29] as BgSRR1 in their QTL mapping cross between the iBS90 and iM line snail inbred lines (Fig 1B and S1 Fig). LG5R is also just upstream from, but distinct from, the SudRes1 peak identified in *B. sudanica* [15]. Interestingly, the part of chromosome 5 that corresponds to SudRes1 shows elevated Fst in our GWAS (Fig 1B), although it did not rise to significance under our criteria.

PCR genotyping of individual snails at the LG5R region validated the significance of the peak in both the GWAS snails and in the validation sample (Fig 2 and S4 File). In the GWAS snails (N = 1182) we see evidence for partial dominance of the R allele: 0.28 of RS individuals were infected, 95% CI = 0.22-0.35, versus the midpoint between the homozygotes of 0.40 (P = 0.001 via the normal approximation test for a single proportion). Binary logistic regression showed modest preference for an R-allele-dominant model over an additive model (ΔAIC = 1.7). Odds ratios (OR) = 3.07 (P < 0.0005) for the dominance model (R_ vs SS) and 2.75 (P < 0.0005) for the additive model (odds of infection 7.6 times higher for SS than for RR individuals).

**Fig 2:**
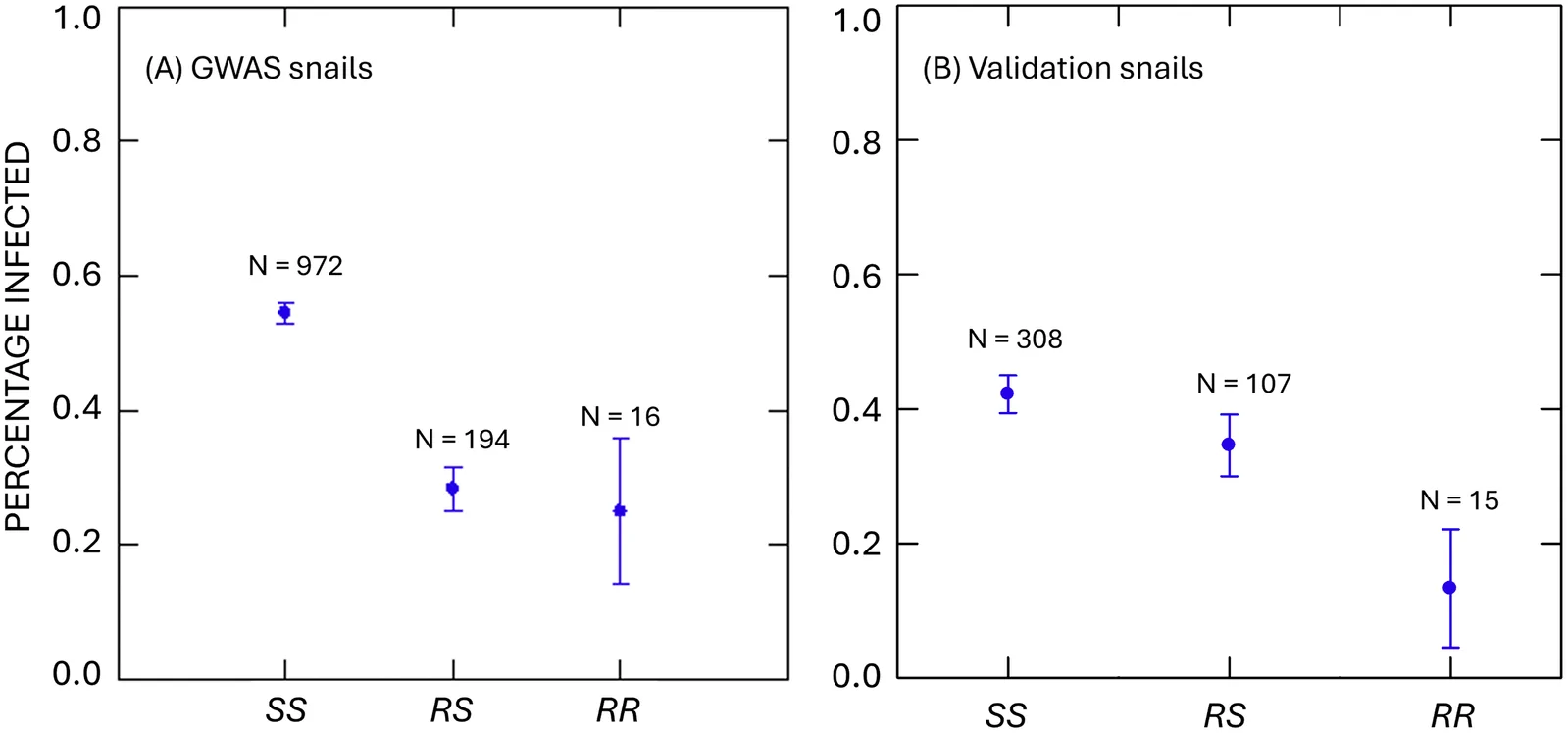
Percentage of snails of each LG5R genotype (RR, RS or SS) that became infected after challenge with 5 miracidia of PR1-population *S. mansoni.* (A) 1182 of the 1200 snails used in the PoolSeq GWAS (frequency of R allele, *f*_*R*_ = 0.10). **(B)** Independent sample of 430 snails for validation (infection rate = 0.39, *f*_*R*_ = 0.16). Error bars are the standard error of the mean. Note that the validation snails were a random sample from the population, while the GWAS snails included equal numbers of cases and controls.

In the validation sample (N = 430) we see little evidence for deviation from an additive model (0.35 of RS individuals infected, 95% CI = 0.26-0.44 vs midpoint between the homozygotes of 0.28). In this sample OR = 1.59 with P = 0.019 for the additive model (odds of infection 2.5 times higher for SS than for RR individuals).

### Physical structure of the region and similarities among assemblies

For the purpose of sequence and gene composition analysis, we analyzed the area under the Fst peak plus about 1 Mb flanking it on the left and right (area between the blue lines for Fig 1B; see S4 Table for details). The LG5R Fst peak is a little less than 2 Mb wide at its base (Fig 1B). Assemblies i90, i171 and i36b carry the S haplotype while i46 and i68 carry the R. Under the main Fst peak, S and R haplotypes diverge from each other, with all the S assemblies resembling iM_Zhong and both R assemblies resembling the BS90 assemblies iBS90_Bu and FSS5 (S2E Fig). Comparison with reference sequences reveals multiple recombination points in the flanking regions (S2 Fig), but none within the Fst peak itself. The R and S haplotypes show moderate levels of sequence divergence across the Fst peak region (BLAST identity ≥90%), save for a non-homologous ~120kb portion toward the left side that corresponds with a drop in the Fst peak (Fig 3).

**Fig 3:**
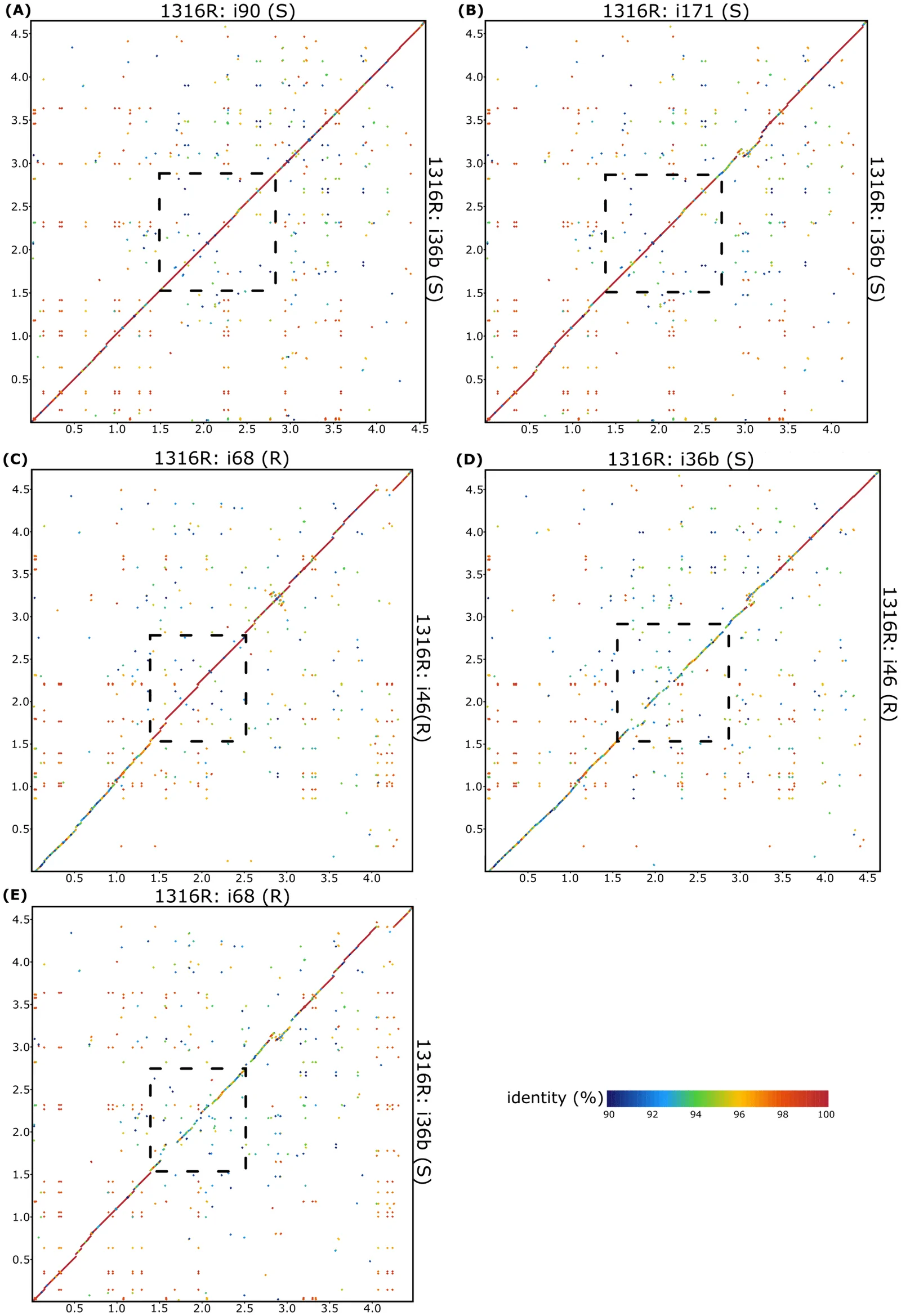
R and S assemblies diverge from one another under the LG5R Fst peak. Dotplots for the LG5R region of association and surrounding sequence between various R and S haplotype 1316R assemblies: A) i90(S) vs i36b(S), B) i171(S) vs i36b(S), C) i68(R) vs i46(R), D) i36b(S) vs i46(R) and E) i68(R) vs i36b(S).The black square marks the rough location of the Fst peak. Axis coordinates refer to the start and end of the regions analyzed for each assembly, see S4 Table for details.

### Genes within the LG5R region

The Fst peak covers ~47 gene models, about half of which appear to be small gene fragments with no identified protein domains (Fig 4 and S5-S12 Tables). In the ~ 120 Kb non-homologous region between R and S haplotypes nine gene models are present on the S version of the block (Gene Model 72–80), eight of which appear to be gene fragments (only gene model 72 appears to be a complete gene). No gene models appear on the R version of the block. References iBS90_Bu and Bg2024 share the R version of this 120 Kb block, and iM_Zhong shares the S version of this block, so we are confident in these conclusions about gene presence/absence (see relevant gene models on S6 Table).

**Fig 4:**
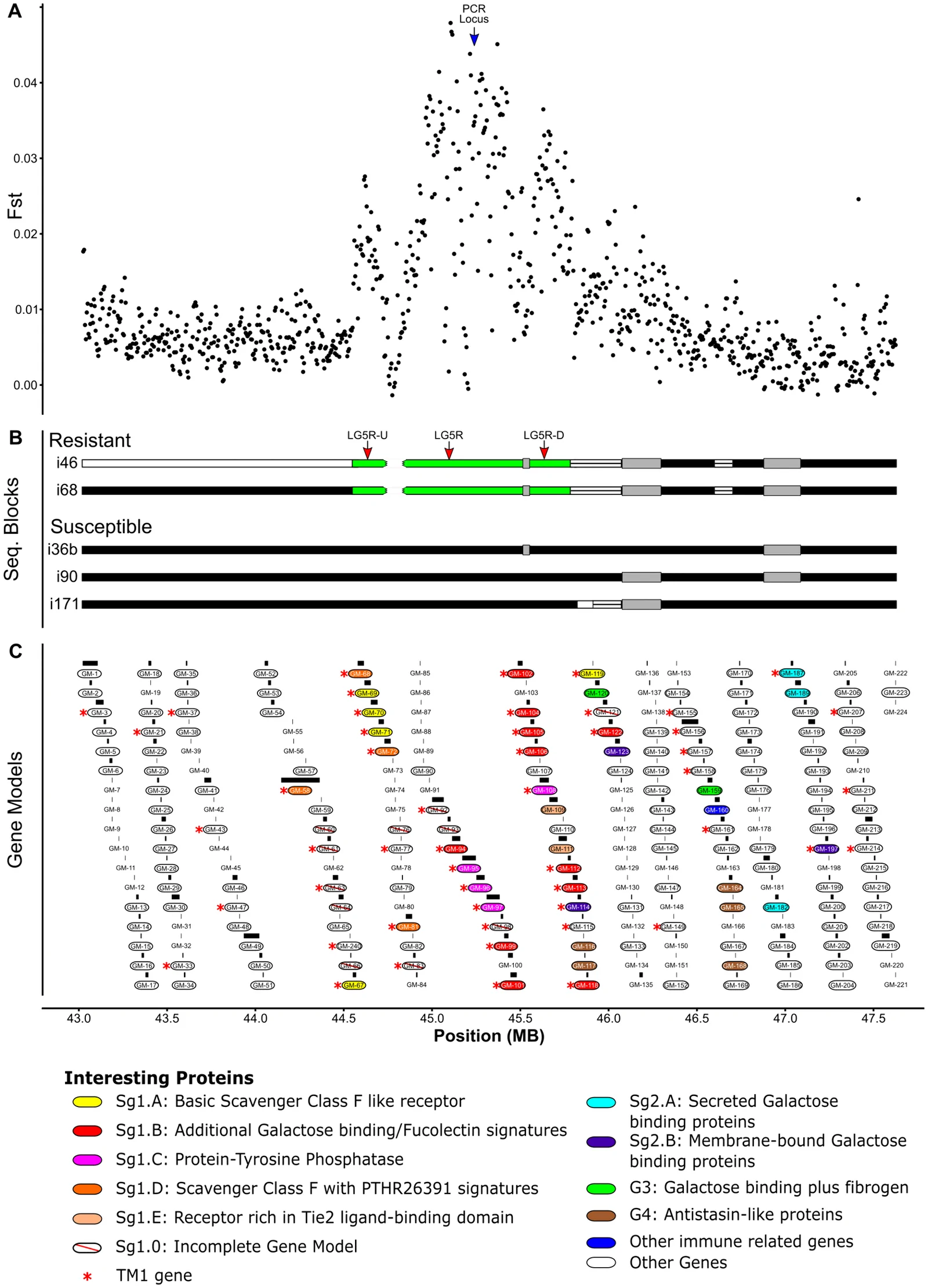
LG5R contains a cluster of genes for single-pass transmembrane (TM1) proteins that share a similar protein domain architecture. Shown are gene models under and around the Fst peak at LG5R. X-axis = position on iM_Zhong chromosome 5 (CM074206.1). **(A)** Sliding window Fst data for the region in and around LG5R. Blue arrow indicates the position of the PCR locus used for validation in Fig 2 above. **(B)** Sequence blocks found on the R and S 1316R assemblies, and key structural features (see S3 Fig for a more in-depth map). Sequence blocks were identified through dotplot comparisons and Liftoff gene reports: Black = Match to iM_Zhong, Green = match to BS90 assembly FSS5, White = no match to a reference assembly, and White with a strike = unknown but similar to other 1316R assemblies. The break in the i46 and i68 bars marks the location of gene models exclusive to iM_Zhong like genomes (the S haplotype on this region of association), while grey blocks highlight regions of non-conserved gene model composition among assemblies. Red arrows mark the positions of the three short haplotypes we used to initially decide which haplotype each 1316R assembly carried (S4 File). **(C)** Gene Model positions. Note the cluster of TM1 genes marked in red and pink under the Fst peak.

Like the PTC1/GRC and PTC2 regions [14,20], LG5R is located in a region that is unusually rich in genes that code for single-pass transmembrane (TM1) proteins (supported by annotation transfers from all four reference genomes; Fig 5). Gene model annotation suggests that in addition to being anchored in the membrane by a single TM domain, genes under the Fst peak in LG5R share a similar protein domain organization. This includes a signal peptide (SP) motif, at least one “Tie2 ligand-binding domain superfamily” signature, and a transmembrane α-helical transmembrane domain. In some examples there is an additional galactose binding and/or fucolectin-like signature in the extracellular region. In most cases the cytosolic domain is small and without identifiable domain signatures with one exception represented by 4 genes (see Subgroup 1C below). Individual Epidermal Growth Factor repeats (EGF) are detected inconsistently among proteins, typically associated with “Tie2 ligand-binding domain superfamily” signatures. In addition, InterProScan reported five protein family signatures competing for the annotation of these membrane receptors, in some cases even for different isoforms of the same gene (Fig 6). The most frequent annotation includes “Multiple EGF-like domains/Integrin beta-like” (Entry: IPR052108) and “MEGF10/SREC1/2-like” (Entry: IPR042635). Both cover the “Scavenger Receptor Class F” protein family according to their entry description, and InterProScan results are similar to human scavenger receptor genes SCARF1 (Uniprot ID: Q14162) and SCARF2 (Uniprot ID: Q96GP6).

**Fig 5:**
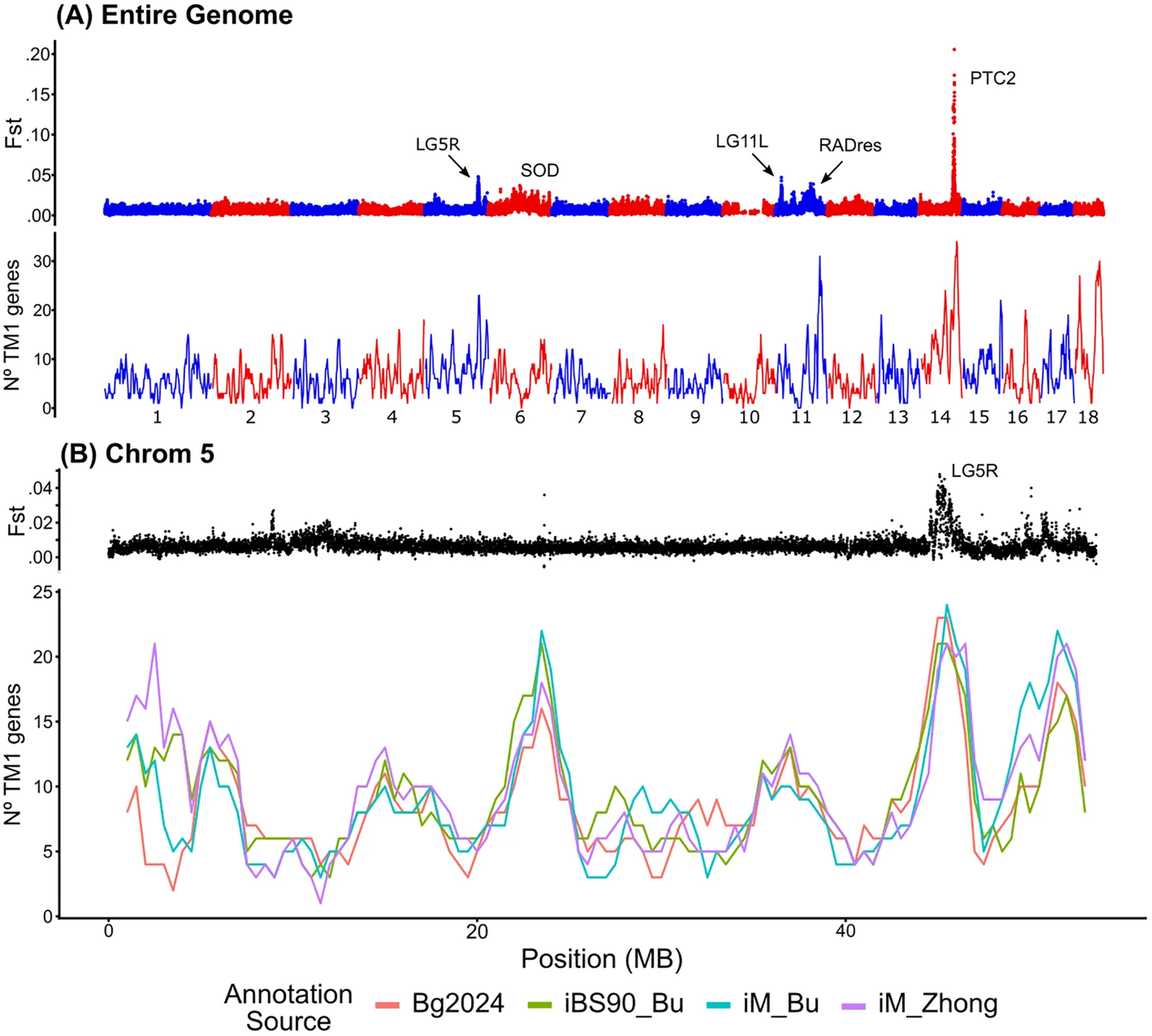
LG5R and PCT2 are in regions of unusually high density of single-pass transmembrane (TM1) genes. **(A)** Fst and density of TM1 genes across the entire genome. Fst plot is a compressed version of Fig 1A. TM1 density was estimated using annotations from the Bg2024 assembly and plotted across the iM_Zhong assembly. Note that the TM1 peak on chromosome 11 is just to right of the RADres region of association, not in it. **(B)** Fst and TM1 density plotted on Chromosome 5 (TM1 in 2Mb sliding windows). Colored lines are TM1 density plotted on the iM_Zhong assembly using annotations from all four reference genomes (iM_Zhong, iM_Bu, iBS90_Bu and Bg2024; legend at bottom of figure).

**Fig 6:**
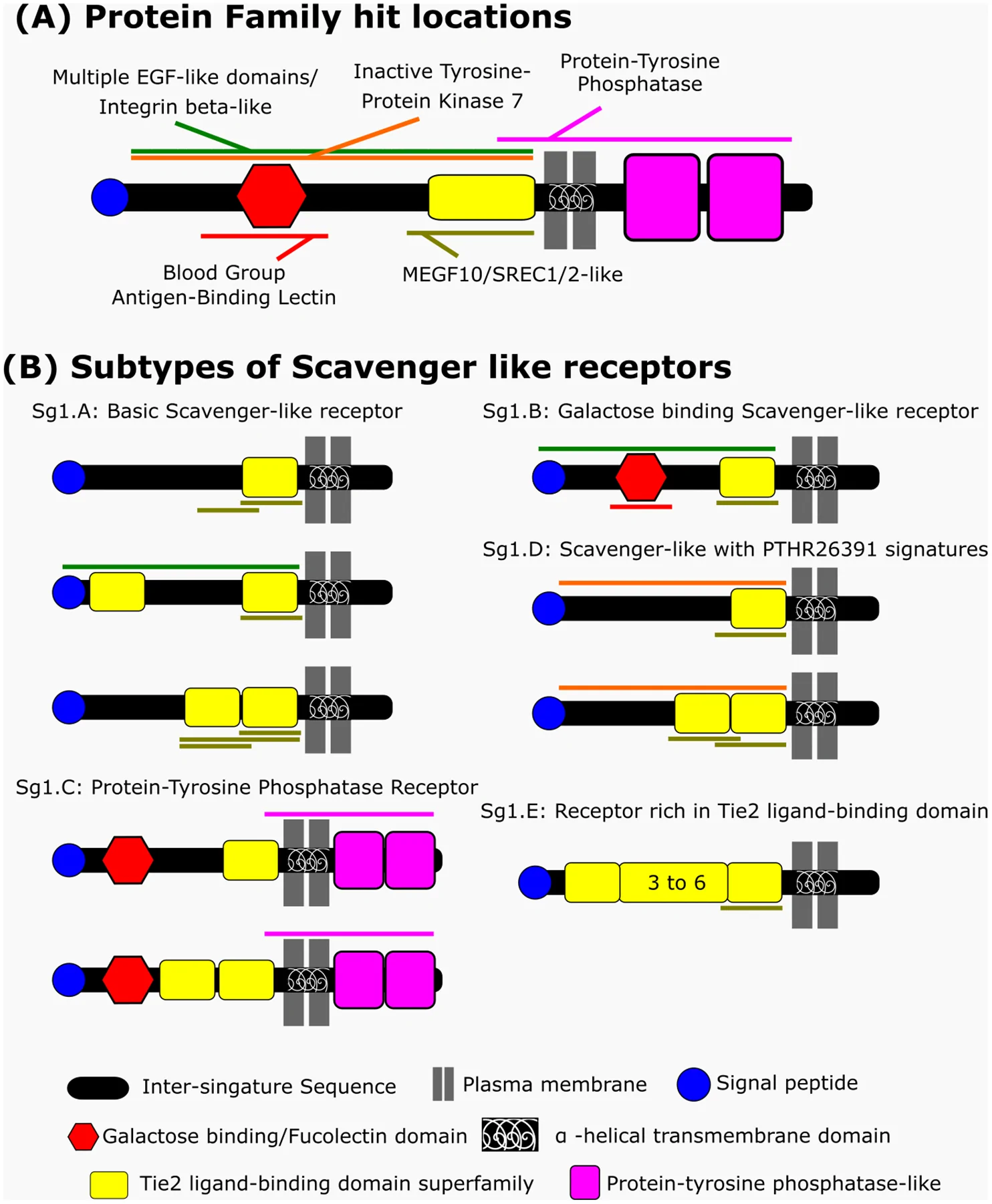
Mock representation of the scavenger-like receptors found in the region of association LG5R. **(A)** Schematic display of the locations of the various protein families identified by InterProScan showing the areas of the protein being identified. **(B)** Domain organization of each subgroup of Potential Scavenger class F-like receptors. Colored lines mark the rough location of protein family hits in at least one of the protein isoforms included in the gene models for that subgroup.

Based on these results we classified the gene models into the same potential protein family named “Potential Scavenger class F-like receptors”, subdivided into 5 subgroups. Genes that lacked the signal peptide or the transmembrane domain, but shared similar domain signatures, were included within this family but as potential fragmented annotations.

- Subgroup 1A: The basic organization described above, with no additional domains. This group is the closest to the human SCARF1and SCARF2 genes in domain signature composition.
- Subgroup 1B: Galactose binding/Fucolectin signatures in the extracellular region
- Subgroup 1C: like 1B but with two “Protein-tyrosine phosphatase-like” domain signatures in a comparatively large cytosolic region.
- Subgroup 1D: Like 1A but with signatures for “Inactive Tyrosine-Protein Kinase 7” (Panther ID: PTHR26391, not related to the human “Tyrosine-Protein Kinase 7” gene, see supplementary material) instead of “Multiple EGF-like domains/Integrin beta-like”.
- Subgroup 1E: Multiple signatures for “Tie2 ligand-binding domain superfamily” across the entire extracellular region.

Scavenger receptors are a highly diverse family that are grouped together by their role in the elimination of targets bearing Damage-Associated Molecular Patterns (DAMPs) or Pathogen-Associated Molecular Patterns (PAMPs) [55–57]. Subgroups 1B and 1C probably identify a glycan of some kind (presumably one containing fucose and/or galactose [58]). Subgroup 1C is noteworthy due to their intracellular phosphatase domains, which may play a direct role in signal transduction [59,60].

### Reciprocal BLAST search against *B. sudanica*

The Potential Scavenger class F-like membrane receptors domain structure detected here matches those in the SudRes1 region recently described in *B. sudanica.* In particular, Subgroup 1C matches the receptor-like tyrosine-specific protein phosphatase (RPTP) receptors in their domain composition [15], and both are in proximity with Antistasin-like genes (Gene Models 116 and 117, located just outside the Fst peak). To assess the orthology between both regions we carried out a reciprocal blast search between proteins reported for both species (Bg2024 vs BS111). A reciprocal best hit was found for three similar receptors in both species (Gene Model 95, 97 and 108), but none of them form part of SudRes1 (S13 Table). Instead, they are consistent with one of the additional concentrations of similar genes reported by Pennance et al. [15]. Thus, LG5R is not orthologous with SudRes1.

SudRes1 itself appears to be located further downstream of LG5R on chromosome 5 in iM_Zhong (approximately CM074206.1: 50.2 to 50.9Mb). Curiously, none of SudRes1 RPTP genes had clear reciprocal hits in our analysis (S14 Table) and we are inferring the position of the SudRes1 region in iM_Zhong by the unambiguous identity of other genes encoded in the *B. sudanica* genome’s contigs reported to form the region. This limits our ability for straight forward comparison based on orthology, though genes LOC106059782 vs BSUD.17727 are close to meeting our thresholds. Furthermore, the SudRes1 region shows elevated Fst in our GWAS that did not meet our cutoff criteria for significance (visible on Fig 1B) and is also enriched for TM-1 genes (Fig 5B).

### Variants between R and S haplotypes described within this region

Based on the dotplot analysis against the references, iM_Zhong was selected for variant identification and evaluation since it matches closely the S haplotype in LG5R. In total 7,337 variants met our criteria to be counted within this region of association, of which 231 were classified as Moderate and six as High impact, meaning they are likely to change protein function (see S15 Table, and raw VCF data for the region on S5 to S24 Files). Most annotated genes within the Fst peak have several moderate impact variants that differ between R and S haplotypes, which makes it difficult to narrow down the list of candidate genes under the Fst peak based by this criterion alone. Nevertheless, two gene models possess high impact variants and merit further discussion. Gene Model 90 was annotated as “E3 ubiquitin-protein ligase SopA” and possesses a premature stop codon in the R haplotype (SNP on 44,952,250). Gene Model 104 possesses three High Impact variants: two linked deletions that combine for the removal of four amino acids within the Fucose/Galectin binding domain (coords 45,544,115 and 45,544,115 in iM_Zhong) and a premature stop codon before the TM domain (coord 45,549,714 in iM_Zhong). Both variants are found in the R haplotype, and therefore the gene is likely not functional on this haplotype.

### Summary of LG5R

To summarize, LG5R bears multiple genes in the region that are plausible candidates for modulating the immune response against *S. mansoni* in some way (either by identifying the pathogen or being tricked by it into prematurely shutting down or diverting the response). They consist of TM1 genes that share a similar protein domain structure and appear to be related to the Scavenger Class F protein family. These genes are similar in structure to those identified in the SudRes1 region of *B. sudanica*, which is only a few megabases downstream of LG5R. Because most of the genes under the Fst peak show potentially function-altering substitutions between alleles on the R and S haplotypes, it is difficult to use this criterion to narrow down the list of candidates. Nevertheless, Gene Models 72 and 104 are particularly interesting as they are only found on the S haplotype, and as such might be targeted in some way by *S. mansoni*.

### Region of association LG11L

On chromosome 11 there were two strong peaks that stood out via our conservative inclusion criteria (Fig 1A and 1C). First, there was a broad, diffuse peak surrounding the original *RADres1* locus of Tennessen, Bonner, et al. [19], so we retained the name RADres for that peak. Second, there was a strong, sharp peak (highest mean Fst window ~ 0.05) far upstream that we named LG11L (for linkage group 11 left). Inspection of the Fst plot for just chromosome 11, using the more liberal inclusion criterion of minimum 5 SNPs per window, suggested there might be a third peak in the middle of the chromosome. However, that region also shows elevated Fst in the two 50:50 mixture comparisons, including one window with Fst > 0.06 (S1 File). It is also a region of low read coverage, extreme structural diversity and very low identity between haplotypes (S4 Fig)**.** So, we did not call that middle peak as a true region of association with the resistance phenotype. For the purpose of sequence and gene composition analysis, we analyzed the region spanning the Fst peak and 1 Mb of flanking sequence on either side (area between the blue lines in Fig 1C; see S4 Table for details).

PCR genotyping of individual snails at the LG11R region validated the significance of the peak in both the GWAS snails and in the validation sample (Fig 7 and S4 File). In the GWAS snails (N = 1192) we see evidence for partial dominance of the R allele, with 0.49 of RS individuals infected (95% CI = 0.45-0.53) versus 0.56 as the midpoint between the homozygotes (P < 0.0005). Binary logistic regression also gave stronger support for an R-allele-dominant model than an additive model (ΔAIC = 3.5). Odds ratios (OR) = 2.57 (P < 0.0005) for the dominance model (R_ vs SS) and 1.59 (P < 0.0005) for the additive model (odds of infection 2.5 times higher for SS than for RR individuals).

**Fig 7:**
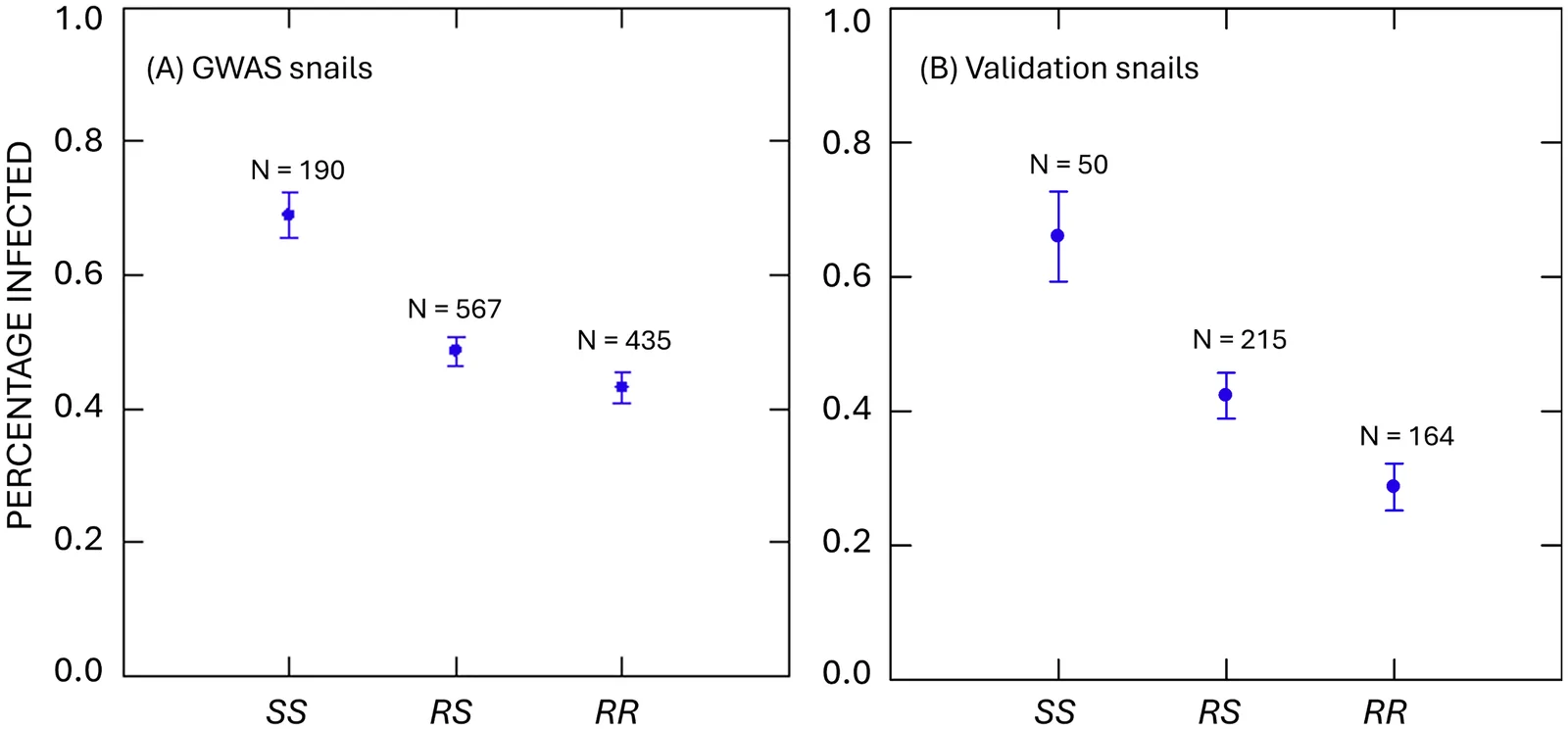
Percentage of snails of each LG11L genotype (RR, RS or SS) that became infected after challenge with 5 miracidia of PR1-population *S. mansoni.* (A) 1192 of the 1200 snails used in the GWAS. *f*_*R*_ = 0.60. **(B)** Independent sample of 429 snails used for validation. *f*_*R*_ = 0.63. Error bars are the standard error of the mean.

In the validation sample (N = 429) we see a similar trend toward partial dominance of the R allele, but not significantly so: 0.42 of RS individuals were infected (95% CI = 0.36-0.49) versus 0.47 as the midpoint between the homozygotes (P = 0.14). Under an additive model the OR = 1.59 (P = 0.019), such that the odds of infection were 2.5 times higher for SS than for RR individuals.

### Physical structure of the region and similarities among assemblies

The LG11L region of association’s main Fst peak is approximately 0.45 Mb wide. Assemblies i36b and i46 have the S haplotype for the region, while i68, i90 and i171 carry the R haplotype. The three R assemblies are very similar to each other, and the two S assemblies are very similar to each other, but R and S diverge for most of the analyzed sequence (Fig 8 and Dot plots in S5 Fig). There appears to be a recombination hot spot at the right-hand edge of the Fst peak (between gene models 56 and 71) where we observe multiple breakpoints in which reference best matches each 1316R assembly (Fig 8B).

**Fig 8:**
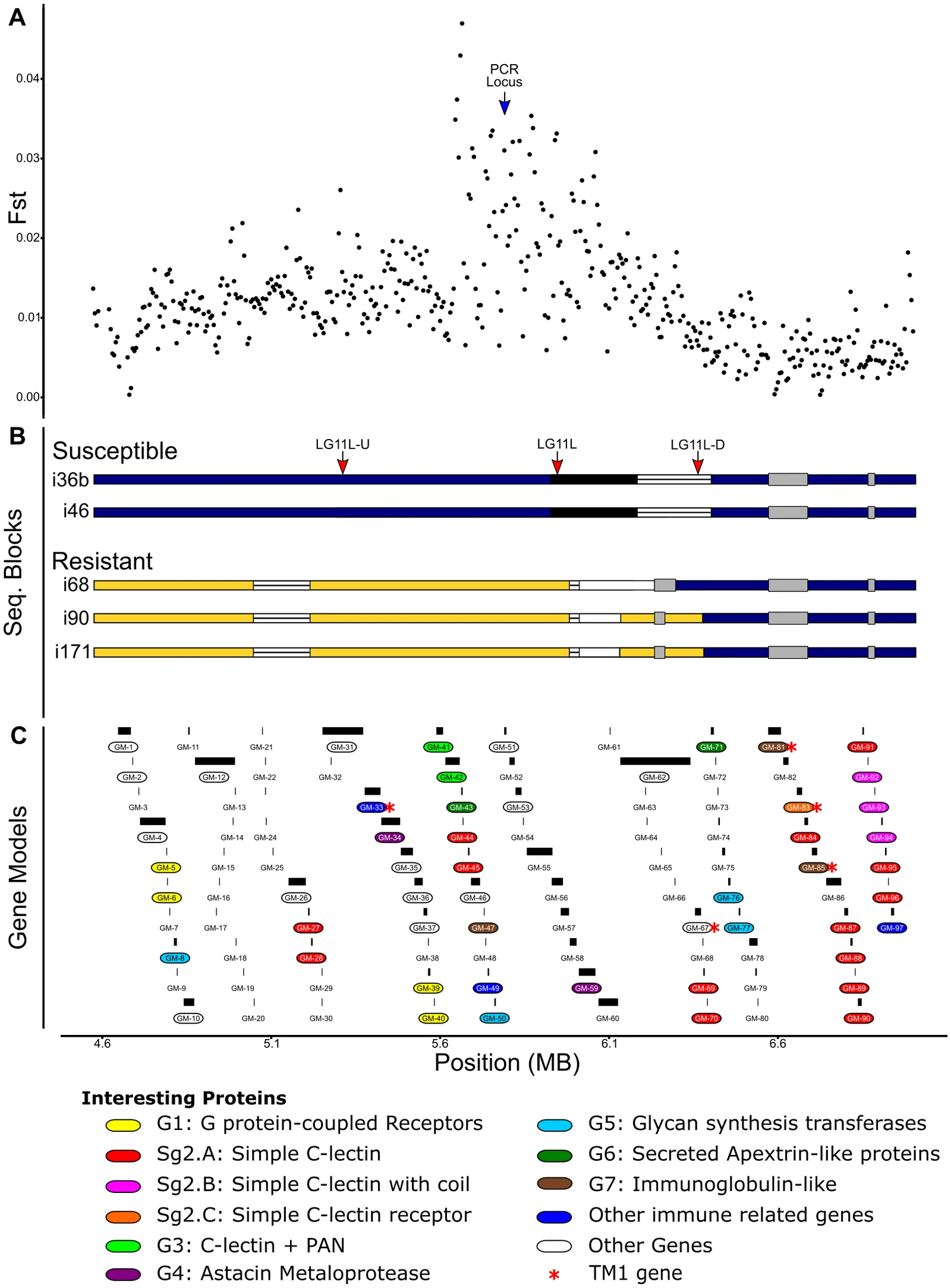
Haplotype blocks and gene content under LG11L X-axis = position on iM_Zhong chromosome 11 (CM074212.1). **(A)** Sliding window Fst data covering LG5R and flanking sequence. Blue arrow = PCR locus used for validation in Fig 7 above. **(B)** Representation of the sequence blocks found on the R and S assemblies, and key structural features (see S6 Fig for a more in-depth map). Sequence blocks were identified through dotplot comparisons and Liftoff gene reports, with colors representing which reference assembly each block resembles (if any): Grey = Bg2024, Black = iM_Zhong, Yellow = iBS90_Bu, White = unknown, White with a strike = unknown but like other 1316R assemblies. Grey blocks highlight regions of non-conserved gene model composition. Red arrows mark the positions of the three short haplotypes we used to initially decide which haplotype each 1316R assembly carried (S4 File). **(C)** Gene Model positions plotted on iM_Zhong.

### Genes within the region of association LG11L

The main peak of association contains approximately 19 genes (gene models 42–60 in Fig 8). Two appear as gene fragments, and eight genes were of inconclusive annotation and could not be assigned potential function. Unlike other regions of association, LG11L is not enriched for TM1 genes, and the genes found under the peak are more structurally diverse. Interestingly, several genes are encoded in pairs, with at least one case of a recent secondary loss (i.e., Gene Model 77 is missing on Bg2024-like assemblies but is present on iM_Zhong and iBS90_Bu, and sequence divergence between them suggests it is not a recent duplication). We speculate that LG11L and flanking regions have been subject to frequent tandem duplications, although this has not led to the propagation of a single protein family within this locus, as appears to be the case in LG5R.

Interesting candidates within this region include the following.

- Gene Model 41 (and adjacent gene model 42) possesses PAN/Apple domain, followed by a C-lectin. PAN/Apple domains mediate protein-protein and/or protein-carbohydrate interactions [61] while C-lectins are a highly diverse domain family specialized in pattern recognition [62]. Different annotations conflict over a 4 or a 2-domain protein composition (one or two repeats).
- Gene Model 43 has an apextrin-like domain, which is less studied relative to C-lectins, but the gene shares the same structure as mediators of bacterial recognition in amphioxus [63].
- Gene Model 44 and 45 are small, secreted C-lectin genes.
- Gene Model 49 was annotated as a fibrinogen-related domain containing protein in all references, but with discrepancies on the specifics (FREP24 in iBS90_Bu and iM_Zhong, FREPA in iM_Bu, and just “fibrinogen-like protein A”), and we confirmed the presence of a C-terminal Fibrinogen domain. Similar proteins are well-known part of the snail immune system [64] and the fibrogen domain is known to have lectin activity in proteins like Ficolins in humans [65].
- Gene Model 47 is a membrane receptor with an immunoglobulin domain, which by itself suggests a role in cell binding and communication like immune response, development and tissue organization [66]. It is noteworthy that the gene is present in the S haplotype and absent on the R haplotype of the 1316R assemblies.
- Gene Model 59 is a multi-domain Astacin Metalloprotease located right at start of the region of high recombination. Such genes can be involved in a wide range of functions, from tissue differentiation, development stages or digestion [67–69].

Interesting to note, genes that are better described as secreted lectins are all concentrated toward the left of LG11L and trend to follow the architecture: “Signal Peptide, spacer sequence, binding domain with lectin activity”. The only exceptions are Gene Model 41 and 42 that could be the result of gene fusion.

### Variants described within this region

Based on the dotplot analysis against the reference assemblies, Bg2024 was selected for variant identification and evaluation since it matches closely the S haplotype in LG11L. In total 14,307 variants met our criteria to be counted within this region of association, of which 319 were classified as Moderate, and five as High impact, variants (see S15 Table, and raw VCF data for the region on S5 to S44 Files). Two gene models within the Fst peak have High impact variants: Gene Model 52 has no known function; Gene Model 53 is a “mitochondrial aspartate/glutamate transporter”, but only some isoforms of this gene are affected (splice acceptor variant at 38,028,623 on Bg2024, seq. ID NC_074721.1). Most genes have moderate impact variants impacting their protein sequence. Among them Gene Model 44 stands out with 26 non-synonymous substitutions, though this high number is a mirage caused by alternative splicing. Two of the 3 reported isoforms for this gene have different C-lectin domains exons (Gene ID: LOC129928991, isoforms XM_056045621.1 and XM_056045622.1 vs XM_056045623.1). The main difference between them is a mutually exclusive terminal exon that contains a different C-lectin domain and likely arose through a partial tandem duplication of the gene.

### Summary LG11L

LG11L contains multiple genes that could explain the observed resistance against *S. mansoni* by playing the role of secreted lectins. As such, one or more of the genes in the region may recognize the parasite and help coordinate the snail immune response against them by recruiting additional proteins. While most of them share a similar organization, their terminal binding domains are diverse with no common origin. At a broader scale, the Fst peak appears to be located next to a recombination hotspot. The observed gene arrangement in pairs suggests multiple small scale tandem duplications affecting one gene at a time, with at least one case of recent gene loss. These observations paint a picture of structural instability that could drive higher rates of evolution.

### Re-annotation of PTC1/GRC and PTC2 genes

The genes in the PTC1/GRC and PTC2 regions were described 5–10 years ago. So, to compare them with the genes in LG5R and LG11L, we re-annotated them using the same methodology applied here (S16-S18 Tables). Both regions are located within local concentrations of TM1 genes, though the specifics of their location and/or magnitude are dependent on the source annotation, particularly for PTC2, which is characterized by multiple large indels among haplotypes [20].

The PTC1/GRC genes can be subdivided into two classes based on the presence and number of two kinds of signatures: Fibronectin type 3 domains and intrinsically disordered regions with polar amino-acids (Fig 9 and S16-S18 Tables). Fibronectin type 3 domains get their name from the glycoprotein fibronectin, where it plays key roles in its conformation and binding to other molecules [70]. On the other hand, disordered regions in the protein are regions whose 3D structure is unstable under physiological conditions. Given their location on these receptors, they likely represent additional binding sites to other proteins [71]. On PTC2 we were only able to identify IG-like domain signatures in six proteins that share the same organization: Signal peptide, IG-Like domain and then the TM domain (Fig 9 and S16-S18 Tables). Proteins containing this type of domain have been associated with multiple cellular functions that involve cell binding and communication, including immune response, development and tissue organization (see [66]).

**Fig 9:**
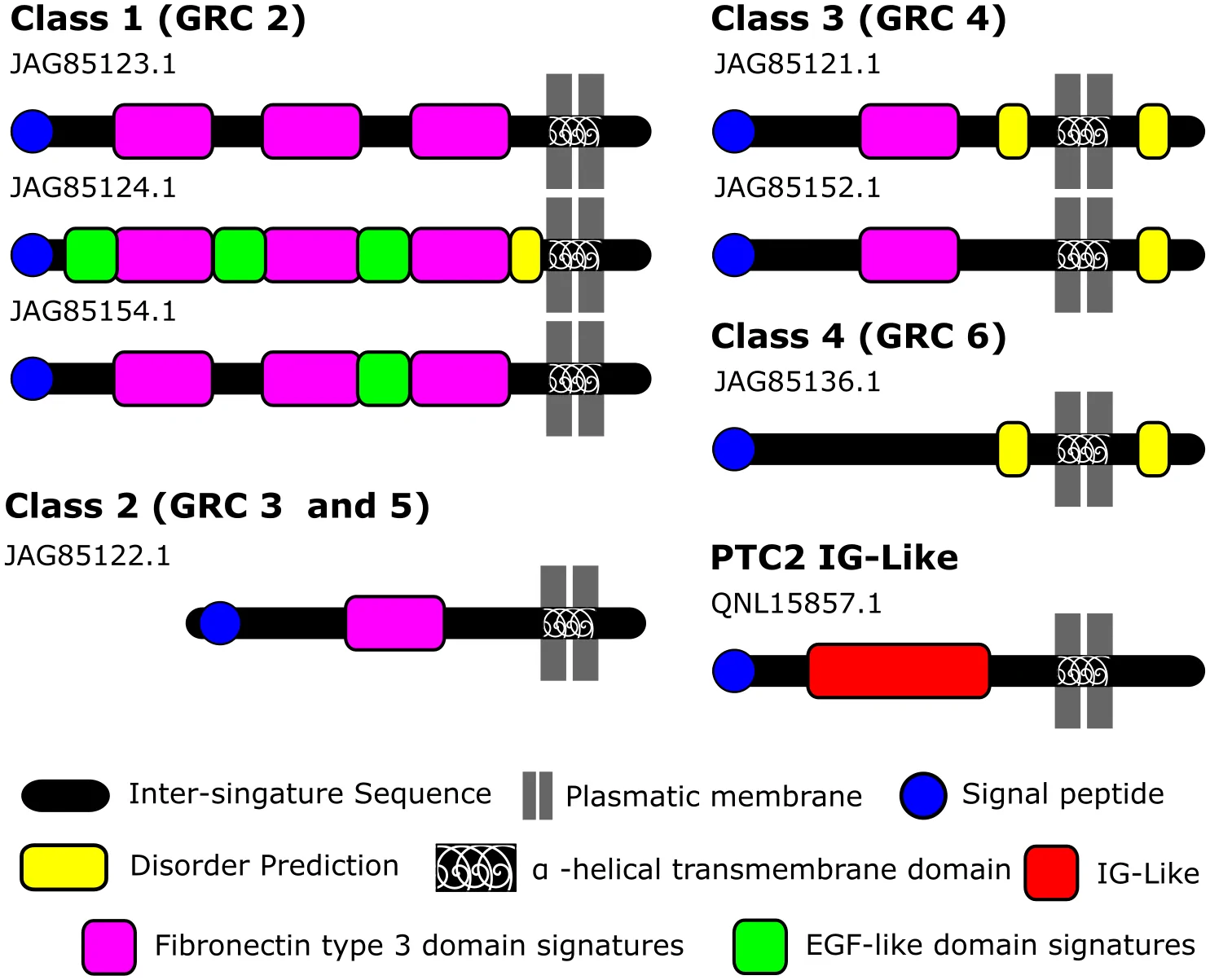
Different classes of TM1 receptors found in the regions.

## Discussion

Reanalysis of the GWAS data from Tennessen et al. [20] using an improved reference genome identified two new regions of association, LG5R and LG11L. In both regions the R and S haplotypes are highly divergent in sequence, with most coding genes showing allelic variation that likely alters protein function. At this point, causal genes and mutations responsible for schistosome resistance have not yet been identified. Nevertheless, it is worth highlighting the protein-coding genes that strongly suggest a potential immune function.

The LG5R region is enriched with TM1 genes, a pattern also observed at the PTC1/GRC, PTC2 and neighboring SudRes1 regions. The cluster of galactose binding/fucolectin domain genes that characterize LG5R is particularly intriguing for several reasons. First, they are concentrated in the tip of the Fst peak, the region of highest statistical association with resistance. Secondly, hemocytes that differed in PTC1/GRC region genotype responded differentially in oxidative burst to galactose, a common PAMP [72]. Thirdly, fucosylated glycans are unusually abundant in the glycoproteome of *S. mansoni* adults [73], which would make them an abundant target if the same is true in the snail stages. Fourth, these genes resemble those in the adjacent SudRes1 region, which showed strong association with resistance in *B. sudanica*. Thus, one or more of these galactose binding/fucolectin domain genes are very plausible candidates to be the causal gene/s in this region. LG11L on the other hand, contains multiple genes that appear to code for secreted proteins with lectin activity. Thus, genes in the LG11L region may reveal a similar function - recognition of pathogen PAMPs - but through a different signaling pathway. We speculate that, like the putative LG5R membrane receptors, these proteins are involved in identifying the parasite.

### What have we learned about snail-schistosome interactions from genome-wide mapping studies to date?

As there have now been multiple genome-wide mapping studies using various populations of *B. glabrata* and *S. mansoni* [14,20,26,29,31,38], and one using *B. sudanica* and *S. mansoni* [15], we can now speculate about commonalities. One interesting result is that these studies don’t repeatedly find the same genomic regions. Instead, different regions appear as major QTL in studies that use different populations of *B. glabrata* and *S. mansoni*. For example, the PTC2 region has a huge effect in 1316R snails challenged by SmPR1 schistosomes, but zero effect in BgGUA population snails challenged by SmGUA population schistosomes [26]. Another pattern is the repeated discovery of regions that contain clusters of genes seemingly involved in PAMP/DAMP recognition or cell communication. Typically, they consist of membrane bound receptors like those in the LG5R, the PTC1/GRC [26], PTC2 [14,20] and the SudRes1 and SudRes2 regions from *B. sudanica* [15]. These observations are interesting in light of two other well-documented phenomena. Firstly, both resistance of *B. glabrata* to *S. mansoni*, and infectivity of *S. mansoni* to *B. glabrata*, are highly heritable traits and easily selected for [21,24,26,74,75]. Secondly, snail populations that are highly resistant to one *S. mansoni* population often remain highly susceptible to other *S. mansoni* populations, and *vice versa* for parasite populations’ abilities to infect different snail populations [22,24,76,77]. These observations are all consistent with the hypothesis that some type of matching-alleles or gene-for-gene interactions [78,79] are behind the ubiquitous compatibility polymorphisms we observe in schistosome-snail interactions (e.g., [25]). We hypothesize that the clusters of putative recognition loci that association mapping studies keep turning up are involved in those interactions. If so, this has substantially broadened our view of the likely molecular basis of compatibility polymorphisms in snail-schistosome interactions beyond the classical FREP-SmPoMuc model [80].

It might be considered surprising that loci for many proteins that are well-known to be involved in snail immunity against schistosomes have never been identified in mapping studies (e.g., BgTLR [81], BgTEP [82] and the FREPs cluster on chromosome 13 [32]. However, a locus will stand out in a GWAS only if there is allelic variation in the snail population that is functionally important in the context of the particular parasite population used to challenge it. Also, it seems likely that different components of the immune system may be called into play in different host-parasite population combinations. Thus, studies using a variety of host-parasite population pairs remain useful – every new locus that is identified gives us further insight into the snail immune system.

### Speculation about immune related loci evolution in *Biomphalaria*

The LG5R, PTC1/GRC and PTC2 regions are all enriched with genes that code for proteins that are anchored to the plasmatic domain through a single alpha-helix TM domain, and that show evidence of copy number variation. This organization is consistent with a history of ancient gene duplications and later sub and/or neofunctionalization [83,84]. Yet each region seems to be enriched for a different family of proteins. This variation in types of receptors that are all putatively involved in some type of PAMP recognition mirrors the diversity of pattern recognition receptors that have evolved in invertebrates [85]. In the absence of a vertebrate-style mechanism of somatic rearrangements to diversify receptors, invertebrates have relied on the presence of large gene families that encode a wide variety of protein isoforms, and on genomic instability to promote sequence variation within those families (unequal crossovers, gene conversion, paralogous mispairing, and so on) [85]. We see evidence of such instability in our TM1-enriched regions such as the PTC2, which is characterized by multiple large indels and gene presence/absence among haplotypes [14,20], patterns typical of regions under various forms of diversifying selection [86,87]. The similarities between LG5R and SudRes1, namely the membrane receptors they contain and their association with antistasin genes, highlight that these regions can be duplicated partially or in their entirety. Furthermore, the fact that regions containing members of the same protein family were found associated with resistance against the same pathogen in two species highlight their importance in the snail immune system.

LG11L on the other hand contains a small but diverse collection of secreted lectins with different binding domains, and while there is evidence of repeated tandem duplications (they seem to be organized into gene pairs) no one protein family dominates the region under the peak. However, most of the genes in the region have a comparable structure organized as “signal peptide + spacer + lectin domain”, even if the binding domains have variable origin. Furthermore, the region immediately to the right of LG11L contains a cluster of secreted simple C-lectin genes that suggest tandem duplication and later diversification as in the LG5R. Therefore, the lectin cluster inside LG11L might be a splinter of the C-lectin cluster, that later underwent repeated exon shuffling. Such events have been suggested in other organisms without adaptative immune systems as a mechanism to generate variation (e.g., [88,89]) and such shuffling would be facilitated by the binding domain being encoded in a single exon for most lectins in the region. The apparent recombination hotspot immediately to the right of LG11L adds to suspicions of genomic instability for the region [85].

### Practical application of this knowledge

We speculate that many regions identified by mapping studies on *B. glabrata* contain clusters of polymorphic receptor genes that are involved in some type of recognition system. Whether such genes would be good targets for gene-drive modification of snail populations is debatable. That is because such systems tend to involve highly polymorphic loci in *both* the parasite and the host. Thus, one might only be able to make snails resistant to their local population of parasites at best, and at worst, the parasite will quickly evolve to match whatever snail alleles are driven to high frequency. We must also consider that schistosomes are not the only pathogens such a system evolved to deal with. For example, we see evidence of balancing selection at the PTC1/GRC region, which suggests fitness tradeoffs exist among alleles [26]. Thus, in the search for loci to use in gene drives, we might be better served by focusing on genes involved in basic effector mechanisms that are downstream of recognition, rather than on highly polymorphic genes involved in some type of matching. Examples might include *sod1*, part of the oxidative burst killing mechanism [16,17,90], and the pore forming toxins biomphalysins [91].

A methodological consideration is that mapping approaches such as GWAS might be biased towards discovery of polymorphic matching loci because they have very large effects, but only in particular host-parasite population combinations (the outsized effect of PTC2 in the 1316R snail by SmPR1 schistosome combination, as a case in point). Indeed, the effector locus *sod1* was initially found via a candidate gene approach, not via genome-wide mapping [92]. Loci in the LG5R, LG11L, SOD and RADres regions all show odds ratios around 2.0 for substituting an R allele for an S. We detected those loci in this dataset because it used N = 600 cases and N = 600 controls. PoolSeq studies using much smaller sample sizes would likely be underpowered to the point where they would have missed such loci. For example, Blouin et al. [14] identified the PTC2 region in BS90 snails using N = 96 cases and 96 controls. But the high background noise would have obscured the signal of loci of lesser effect, such as the four above. So, we recommend substantially larger sample sizes if one hopes to identify allelic variation in other components of the immune system besides large-effect loci involved in matching polymorphisms.

Regardless of whether clusters of highly polymorphic genes would be good targets for genetic manipulation of snails, their discovery has potentially identified fundamental parts of the snail immune system. There may be other ways than gene drives to use this knowledge to block transmission at the snail stage, especially once the matching molecules on the parasite side have been identified.

## Supporting information

S1 Fig BgSRR1 and LG5R are nearby but independent loci in the *B. glabrata genome.* A) Fst measured in our analysis and B) Fst measured in Zhang et al. (2024) and the reported recombination bins supported by more than 5 SNPs (yellow bars bellow the plot). The location of BgSRR1 and LG5R is shown in both panels with dotted blue and red lines respectively.
(PDF)

S2 Fig Dotplot analysis shows link with the iM_Zhong and BS90_FSS5 for the S and R haplotypes for LG5R respectively. A) i36b vs iM_Zhong; B) i46 vs iM_Zhong; C) i46 vs iBS90_Bu; D) i46 vs Bg2024; E) BS90_FSS5 vs i46; and F) iM_Zhong vs Bg2024. Of the observed genomes BS90_FSS5 is the strongest match for the area covered by LG5R Fst peak itself (dotted square in all panels).
(PDF)

S3 Fig Detailed logic map of the LG5R across R and S 1316R assemblies. Information includes: 1) Gene model composition and basic annotation. 2) the best matching reference for each gene according Liftoff identity and coverage metrics. 3) Shared missing genes between 1316R and reference assemblies. 4) Observed number of Moderate + High impact variants assigned to the gene model. 5) Region matching the BS90_FSS5 assembly on the R haplotype (Green bar) and the Fst peak itself (Red bar). And 6) Places with unreliable annotation. Pannel A) Shows the logistic map in general, Pannel B) Details regions of inconsistent Gene Model organization between assemblies.
(PDF)

S4 Fig Dotplot analysis shows the limited homology between reference assemblies in the spurious region of association located between LG11L and RADres. A) Bg2024 vs iBS90_Bu. B) Bg2024 vs iM_Zhong. And C) iM_Zhong vs iBS90_Bu. Region coordinates were defined following the same methodology as LG5R and LG11L: 1) Bg2024 as NC_074721.1: 27,345,383–28,709,944 in Bg2024 (anchored in genes BgiBS90_038122 from BS90_bu_et_al_qtl and LOC106058724 from Bg_2024, analyzed in the reverse complement). 2) iBS90_Bu as JAKZJK010000019.1: 11,329,525–12,323,073 (anchored in BgiMline_021922 and BgiMline_021939, both from iM_Bu) and 3) iM_Zhong as CM074212.1: 15,116,011–16,477,347 (anchored in genes BgiBS90_038122 from BS90_bu_et_al_qtl and LOC106058724 from Bg_2024).
(PDF)

S5 Fig Dotplot analysis shows significant divergence between 1316R assemblies with S and R haplotypes, but internal homogeneity for LG11L. A) i36b vs i46; B) i68 vs iM_Zhong; C) i171 vs i90; D) i46 vs i68. The area of homogeneity extends beyond the Fst LG11L peak itself (dotted square in all panels).
(PDF)

S6 Fig Detailed logic map of the LG11L across R and S 1316R assemblies. Information includes: 1) Gene model composition and basic annotation. 2) the best matching reference for each gene according Liftoff identity and coverage metrics. 3) Shared missing genes between 1316R and reference assemblies. 4) Observed number of Moderate + High impact variants assigned to the gene model. 5) Region matching the iBS90_Bu assembly on the R haplotype (Green bar), the area where all 1316R match the Bg2024 reference genome (red bar), both separated by a transition zone suspected to be a recombination hotspot (Grey Bar), and the Fst peak itself (Red bar below). And 6) Places with unreliable annotation. Pannel A) Shows the logistic map in general, Pannel B) Details regions of inconsistent Gene Model organization between assemblies.
(PDF)

S1 Table Primers using for genotyping individual snails at each of the two loci (LG5R and LG11L) and their locations in the Zhong et al. (2024) genome. All loci were scored as amplicon size on agarose gels.
(XLSX)

S2 Table Short haplotypes used to decide which of the 1316R PacBio assemblies carried R vs S haplotypes at LG5R and LG11L. (XLSX)

S3 Table Genome assemblies quality metrics used in this study and the transferred gene counts from the 4 reference assemblies. (XLSX)

S4 Table Target region location across the genome assemblies used in this study, anchor genes and raw counts of genes found within them from each annotation reference. (XLSX)

S5 Table Location of the transferred genes from each of the Reference Assemblies (Bg2024, iBS90_Bu, iM_Bu and iM_Zhong) to the genome assemblies analyzed in this study. For each case it is specified the original annotation and the Identity and Coverage of the original gene, reported by Liftoff.
(XLSX)

S6 Table Annotation notes for all the gene models described for the target regions. Results for SignalP6, TargetP and TMHMM are summarized as presence “X” or absence “.” in at least one reported isoform for the gene.
(XLSX)

S7 Table InterProScan results for the genes predicted to be located within the regions of association in at least one assembly. (XLSX)

S8 Table Signal Peptide predictions carried out by Signalp6 for the genes reported for the target regions. (XLSX)

S9 Table Mitochondrial Transit Peptide predictions carried out by TargetP for the genes reported for the target regions. (XLSX)

S10 Table Transmembrane alpha helices predictions carried out by TMHMM for the genes reported for the target regions. (XLSX)

S11 Table Coordinates for transmembrane domains identified by TMHMM and the reported cellular regions relative to the plasmatic membrane for the genes found in the regions of association. (XLSX)

S12 Table Assigned KEGG orthologues by KofamKOALA to the genes reported for the target regions. (XLSX)

S13 Table Reciprocal blast genes from Bg2024 and successfully transferred to CM074206.1 (Chr5) in iM_Zhong, vs *Biomphalaria sudanica* proteins included in SudRes1 or (Assembly BS111). Only Genes reported to be part of SudRes1 or sharing a similar descripton to its membrane receptors are included: epidermal growth factor (EGF), galactose binding domain (GBD), multiple epidermal growth factor (MEGF) and forming a receptor-like PTP (RPTP).
(XLSX)

S14 Table Reciprocal BLAST results between reported proteins of *B. sudanica* genome assembly (assembly Bs111) vs *B. glabrata* assembly (assembly Bg2024) identified as RPTP in Pennance et al. [15]. Only gene BSUD.17727 had a reciprocal hit in Bg2024 (gene LOC106059782 but the transfer quality to iM_Zhong was below the 80% coverage threshold.
(XLSX)

S15 Table Variant counts for each gene model, sorted according to their associated group of 1316R assemblies: “S” and “R” for Susceptible and Resistant, plus All 1316R assemblies. And then further categorized by biological impact estimated by SnpEff: Modifier (e.g., intronic variation), Low - (i.e., synonymous substitution), Moderate (i.e., non-synonymous substitution), or High (e.g., stop codon gain/lost, splice site alteration, deletions, etc.). References where pick for their close match to one of the haplotypes for each region of association.
(XLSX)

S16 Table Signal Peptide predictions carried out by Signalp6 for the genes reported for PTC1/GRC and PTC2 regions. (XLSX)

S17 Table Coordinates for transmembrane domains identified by TMHMM and the reported cellular regions relative to the plasmatic membrane for the genes found in the regions PTC1/GRC and PTC2. (XLSX)

S18 Table InterProScan results for the genes from PTC1/GRC and PTC2. (XLSX)

S1 File Methodological details for the Fst peak identification. (PDF)

S2 File Notes on discrepancies in the gene composition found among different assemblies inside the regions of association LG11L and LG5R. The causes of the discrepancies are likely a combination of biological factors (e.g., large structural variants) and artifacts of various causes (e.g., lack of sequence in the assembly forces liftoff to identify the wrong paralog to the gene or report a secondary best hit).
(PDF)

S3 File Methodological details for Variant analysis: Identification, estimated functional impact and count criteria among haplotypes. (PDF)

S4 File Phenotype and genotype data for the validation snails and the GWAS analysis. (XLSX)

S5 File Raw VCF file for the LG5R region comparing the 1316R assembly i36b against the reference assembly iM_Zhong, using the annotation transferred from Bg2024 for variant effect estimation. (VCF)

S6 File Raw VCF file for the LG5R region comparing the 1316R assembly i36b against the reference assembly iM_Zhong, using the annotation transferred from iBS90_Bu for variant effect estimation. (VCF)

S7 File Raw VCF file for the LG5R region comparing the 1316R assembly i36b against the reference assembly iM_Zhong, using the annotation transferred from iM_Bu for variant effect estimation. (VCF)

S8 File Raw VCF file for the LG5R region comparing the 1316R assembly i36b against the reference assembly iM_Zhong, using the annotation transferred from iM_Zhong for variant effect estimation. (VCF)

S9 File Raw VCF file for the LG5R region comparing the 1316R assembly i46 against the reference assembly iM_Zhong, using the annotation transferred from Bg2024 for variant effect estimation. (VCF)

S10 File Raw VCF file for the LG5R region comparing the 1316R assembly i46 against the reference assembly iM_Zhong, using the annotation transferred from iBS90_Bu for variant effect estimation. (VCF)

S11 File Raw VCF file for the LG5R region comparing the 1316R assembly i46 against the reference assembly iM_Zhong, using the annotation transferred from iM_Bu for variant effect estimation. (VCF)

S12 File Raw VCF file for the LG5R region comparing the 1316R assembly i46 against the reference assembly iM_Zhong, using the annotation transferred from iM_Zhong for variant effect estimation. (VCF)

S13 File Raw VCF file for the LG5R region comparing the 1316R assembly i68 against the reference assembly iM_Zhong, using the annotation transferred from Bg2024 for variant effect estimation. (VCF)

S14 File Raw VCF file for the LG5R region comparing the 1316R assembly i68 against the reference assembly iM_Zhong, using the annotation transferred from iBS90_Bu for variant effect estimation. (VCF)

S15 File Raw VCF file for the LG5R region comparing the 1316R assembly i68 against the reference assembly iM_Zhong, using the annotation transferred from iM_Bu for variant effect estimation. (VCF)

S16 File Raw VCF file for the LG5R region comparing the 1316R assembly i68 against the reference assembly iM_Zhong, using the annotation transferred from iM_Zhong for variant effect estimation. (VCF)

S17 File Raw VCF file for the LG5R region comparing the 1316R assembly i90 against the reference assembly iM_Zhong, using the annotation transferred from Bg2024 for variant effect estimation. (VCF)

S18 File Raw VCF file for the LG5R region comparing the 1316R assembly i90 against the reference assembly iM_Zhong, using the annotation transferred from iBS90_Bu for variant effect estimation. (VCF)

S19 File Raw VCF file for the LG5R region comparing the 1316R assembly i90 against the reference assembly iM_Zhong, using the annotation transferred from iM_Bu for variant effect estimation. (VCF)

S20 File Raw VCF file for the LG5R region comparing the 1316R assembly i90 against the reference assembly iM_Zhong, using the annotation transferred from iM_Zhong for variant effect estimation. (VCF)

S21 File Raw VCF file for the LG5R region comparing the 1316R assembly i171 against the reference assembly iM_Zhong, using the annotation transferred from Bg2024 for variant effect estimation. (VCF)

S22 File Raw VCF file for the LG5R region comparing the 1316R assembly i171 against the reference assembly iM_Zhong, using the annotation transferred from iBS90_Bu for variant effect estimation. (VCF)

S23 File Raw VCF file for the LG5R region comparing the 1316R assembly i171 against the reference assembly iM_Zhong, using the annotation transferred from iM_Bu for variant effect estimation. (VCF)

S24 File Raw VCF file for the LG5R region comparing the 1316R assembly i171 against the reference assembly iM_Zhong, using the annotation transferred from iM_Zhong for variant effect estimation. (VCF)

S25 File Raw VCF file for the LG11L region comparing the 1316R assembly i36b against the reference assembly Bg2024, using the annotation transferred from Bg2024 for variant effect estimation. (VCF)

S26 File Raw VCF file for the LG11L region comparing the 1316R assembly i36b against the reference assembly Bg2024, using the annotation transferred from iBS90_Bu for variant effect estimation. (VCF)

S27 File Raw VCF file for the LG11L region comparing the 1316R assembly i36b against the reference assembly Bg2024, using the annotation transferred from iM_Bu for variant effect estimation. (VCF)

S28 File Raw VCF file for the LG11L region comparing the 1316R assembly i36b against the reference assembly Bg2024, using the annotation transferred from iM_Zhong for variant effect estimation. (VCF)

S29 File Raw VCF file for the LG11L region comparing the 1316R assembly i46 against the reference assembly Bg2024, using the annotation transferred from Bg2024 for variant effect estimation. (VCF)

S30 File Raw VCF file for the LG11L region comparing the 1316R assembly i46 against the reference assembly Bg2024, using the annotation transferred from iBS90_Bu for variant effect estimation. (VCF)

S31 File Raw VCF file for the LG11L region comparing the 1316R assembly i46 against the reference assembly Bg2024, using the annotation transferred from iM_Bu for variant effect estimation. (VCF)

S32 File Raw VCF file for the LG11L region comparing the 1316R assembly i46 against the reference assembly Bg2024, using the annotation transferred from iM_Zhong for variant effect estimation. (VCF)

S33 File Raw VCF file for the LG11L region comparing the 1316R assembly i68 against the reference assembly Bg2024, using the annotation transferred from Bg2024 for variant effect estimation. (VCF)

S34 File Raw VCF file for the LG11L region comparing the 1316R assembly i68 against the reference assembly Bg2024, using the annotation transferred from iBS90_Bu for variant effect estimation. (VCF)

S35 File Raw VCF file for the LG11L region comparing the 1316R assembly i68 against the reference assembly Bg2024, using the annotation transferred from iM_Bu for variant effect estimation. (VCF)

S36 File Raw VCF file for the LG11L region comparing the 1316R assembly i68 against the reference assembly Bg2024, using the annotation transferred from iM_Zhong for variant effect estimation. (VCF)

S37 File Raw VCF file for the LG11L region comparing the 1316R assembly i90 against the reference assembly Bg2024, using the annotation transferred from Bg2024 for variant effect estimation. (VCF)

S38 File Raw VCF file for the LG11L region comparing the 1316R assembly i90 against the reference assembly Bg2024, using the annotation transferred from iBS90_Bu for variant effect estimation. (VCF)

S39 File Raw VCF file for the LG11L region comparing the 1316R assembly i90 against the reference assembly Bg2024, using the annotation transferred from iM_Bu for variant effect estimation. (VCF)

S40 File Raw VCF file for the LG11L region comparing the 1316R assembly i90 against the reference assembly Bg2024, using the annotation transferred from iM_Zhong for variant effect estimation. (VCF)

S41 File Raw VCF file for the LG11L region comparing the 1316R assembly i171 against the reference assembly Bg2024, using the annotation transferred from Bg2024 for variant effect estimation. (VCF)

S42 File Raw VCF file for the LG11L region comparing the 1316R assembly i171 against the reference assembly Bg2024, using the annotation transferred from iBS90_Bu for variant effect estimation. (VCF)

S43 File Raw VCF file for the LG11L region comparing the 1316R assembly i171 against the reference assembly Bg2024, using the annotation transferred from iM_Bu for variant effect estimation. (VCF)

S44 File Raw VCF file for the LG11L region comparing the 1316R assembly i171 against the reference assembly Bg2024, using the annotation transferred from iM_Zhong for variant effect estimation. (VCF)
