# Supplementary material for: The growing repertoire of putative pathogen interaction genes in the Biomphalaria glabrata vs Schistosoma mansoni system
Source: PLoS Negl Trop Dis. 2026 Oct 21;20(10):e0014803. doi: 10.1371/journal.pntd.0014803 (PMC13712769; doi:10.1371/journal.pntd.0014803)
Supplement: Supplementary file 1 [file pntd.0014803.s001.pdf]

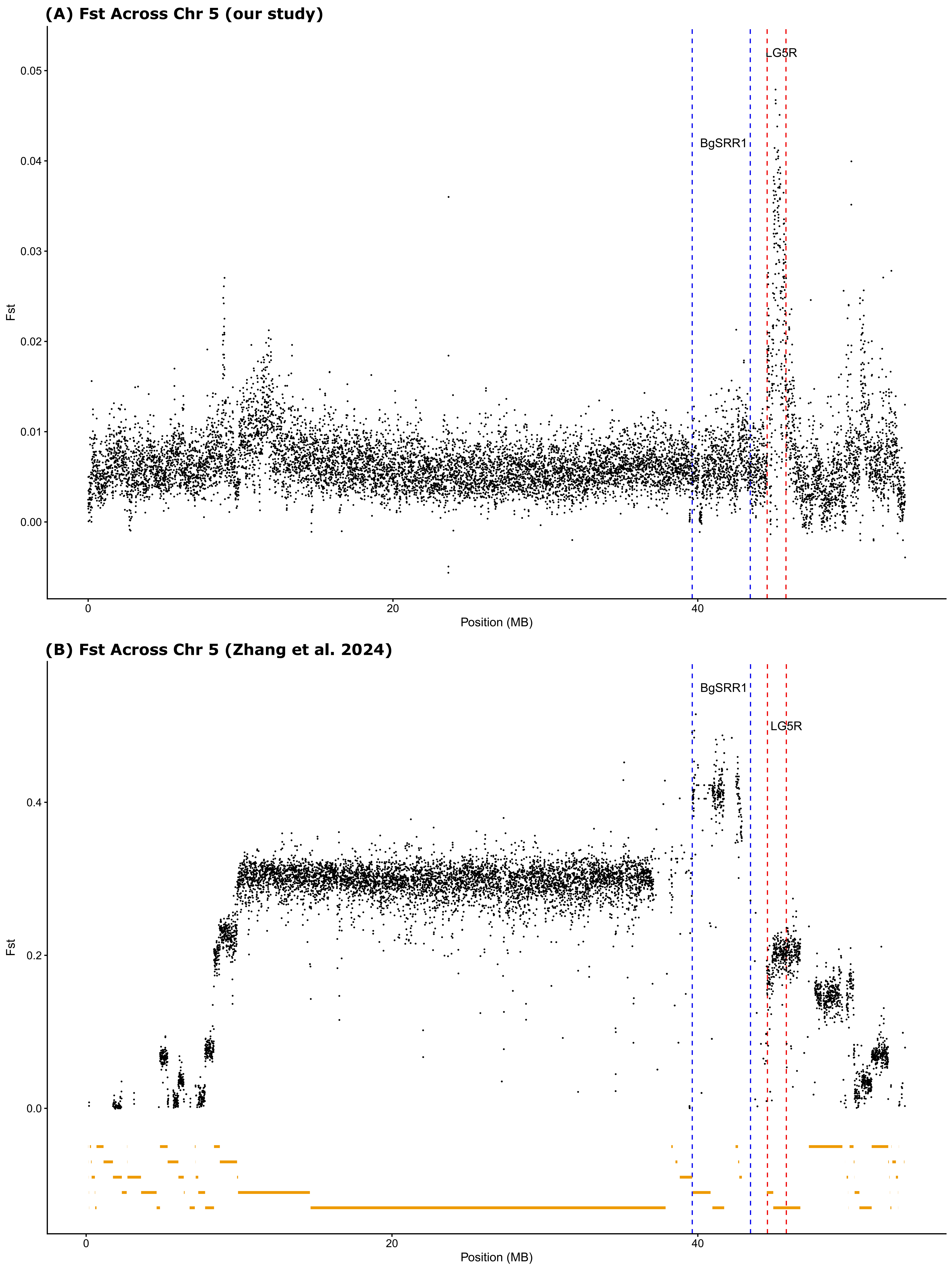

(A) Fst Across Chr 5 (our study)
LG5R
BgSRR1
Fst
0.05
0.04
0.03
0.02
0.01
0.00
0
20
40
Position (MB)
(B) Fst Across Chr 5 (Zhang et al. 2024)
BgSRR1
LG5R
Fst
0.4
0.2
0.0
0
20
40
Position (MB)
