# Supplementary material for: The growing repertoire of putative pathogen interaction genes in the Biomphalaria glabrata vs Schistosoma mansoni system
Source: PLoS Negl Trop Dis. 2026 Oct 21;20(10):e0014803. doi: 10.1371/journal.pntd.0014803 (PMC13712769; doi:10.1371/journal.pntd.0014803)
Supplement: Supplementary file 2 [file pntd.0014803.s002.pdf]

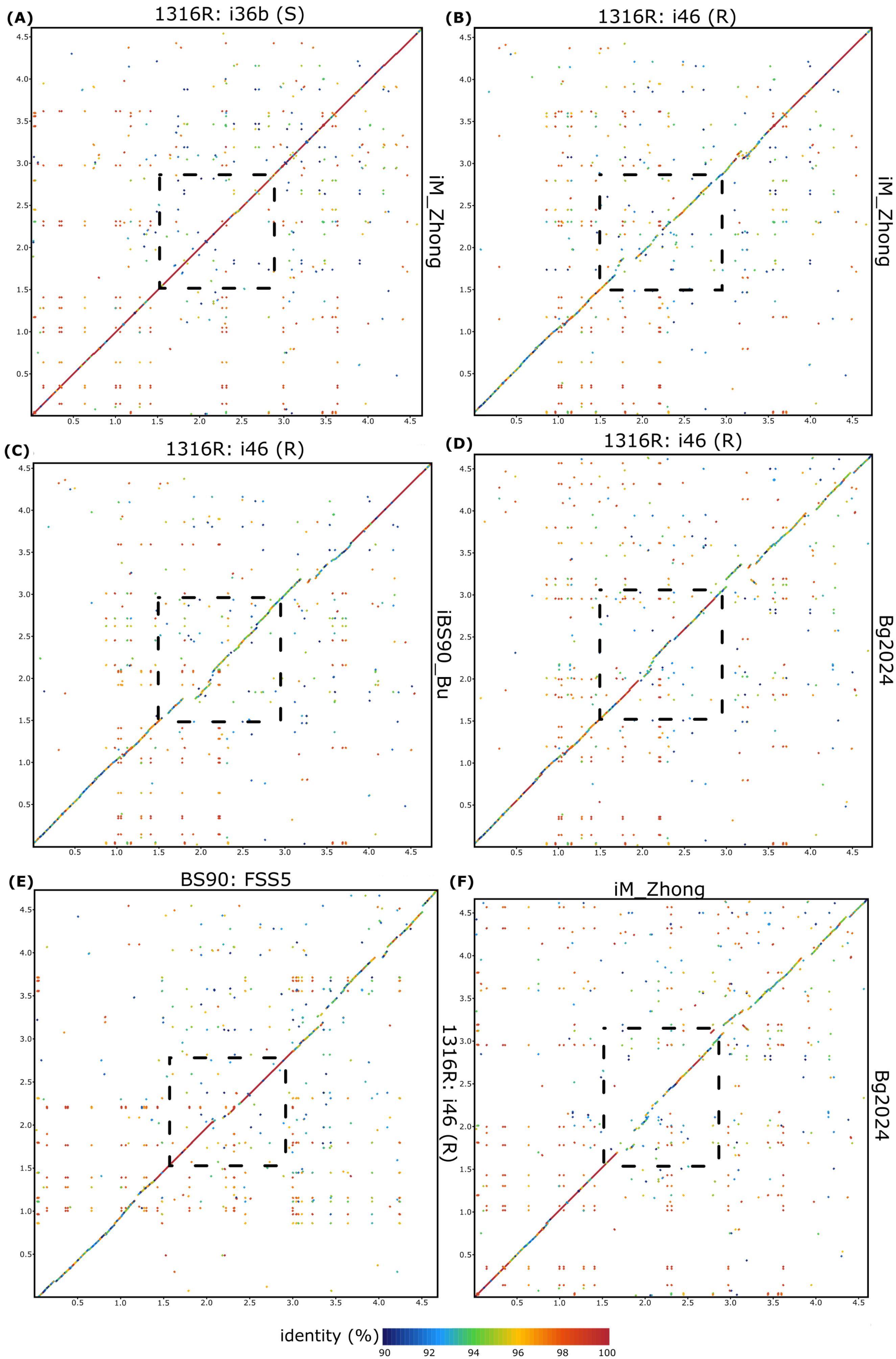

(A) 1316R: i36b (S)
iM_Zhong
(B) 1316R: i46 (R)
iM_Zhong
(C) 1316R: i46 (R)
iBS90_Bu
(D) 1316R: i46 (R)
Bg2024
(E) BS90: FSS5
1316R: i46 (R)
(F) iM_Zhong
Bg2024
0.5
1.0
1.5
2.0
2.5
3.0
3.5
4.0
4.5
identity (%)
90
92
94
96
98
100
