# Supplementary material for: The growing repertoire of putative pathogen interaction genes in the Biomphalaria glabrata vs Schistosoma mansoni system
Source: PLoS Negl Trop Dis. 2026 Oct 21;20(10):e0014803. doi: 10.1371/journal.pntd.0014803 (PMC13712769; doi:10.1371/journal.pntd.0014803)
Supplement: Supplementary file 3 [file pntd.0014803.s003.pdf]

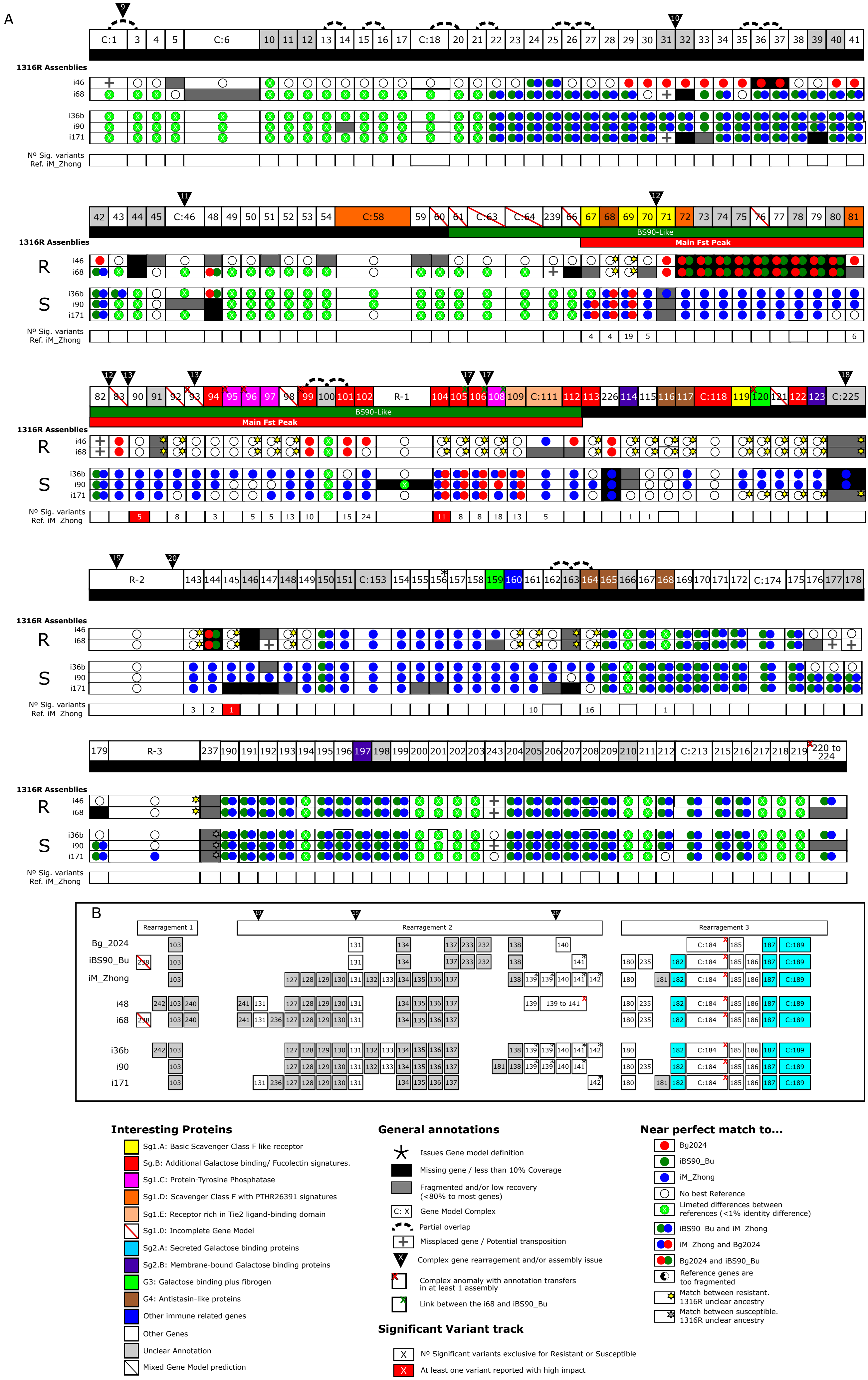

A
C:1 3 4 5 C:6 10 11 12 13 14 15 16 17 C:18 20 21 22 23 24 25 26 27 28 29 30 31 32 33 34 35 36 37 38 39 40 41
1316R Assemblies
i46
i68
i36b
i90
i171
Nº Sig. variants Ref. iM_Zhong
42 43 44 45 C:46 48 49 50 51 52 53 54 C:58 59 60 61 C:63 C:64 239 66 67 68 69 70 71 72 73 74 75 76 77 78 79 80 81
BS90-Like
Main Fst Peak
R
S
4 4 19 5 6
82 83 90 91 92 93 94 95 96 97 98 99 100 101 102 R-1 104 105 106 108 109 C:111 112 113 226 114 115 116 117 C:118 119 120 121 122 123 C:225
5 8 3 5 5 13 10 15 24 11 8 8 18 13 5 1 1
R-2 143 144 145 146 147 148 149 150 151 C:153 154 155 156 157 158 159 160 161 162 163 164 165 166 167 168 169 170 171 172 C:174 175 176 177 178
3 2 1 10 16 1
179 R-3 237 190 191 192 193 194 195 196 197 198 199 200 201 202 203 243 204 205 206 207 208 209 210 211 212 C:213 215 216 217 218 219 220 to 224
B
Rearrangement 1
Rearrangement 2
Rearrangement 3
Bg_2024
iBS90_Bu
iM_Zhong
i48
i68
i36b
i90
i171
Interesting Proteins
Sg1.A: Basic Scavenger Class F like receptor
Sg.B: Additional Galactose binding/ Fucolectin signatures.
Sg1.C: Protein-Tyrosine Phosphatase
Sg1.D: Scavenger Class F with PTHR26391 signatures
Sg1.E: Receptor rich in Tie2 ligand-binding domain
Sg1.0: Incomplete Gene Model
Sg2.A: Secreted Galactose binding proteins
Sg2.B: Membrane-bound Galactose binding proteins
G3: Galactose binding plus fibrogen
G4: Antistasin-like proteins
Other immune related genes
Other Genes
Unclear Annotation
Mixed Gene Model prediction
General annotations
Issues Gene model definition
Missing gene / less than 10% Coverage
Fragmented and/or low recovery (<80% to most genes)
C: X Gene Model Complex
Partial overlap
Missplaced gene / Potential transposition
Complex gene rearragement and/or assembly issue
Complex anomaly with annotation transfers in at least 1 assembly
Link between the i68 and iBS90_Bu
Significant Variant track
X Nº Significant variants exclusive for Resistant or Susceptible
X At least one variant reported with high impact
Near perfect match to...
Bg2024
iBS90_Bu
iM_Zhong
No best Reference
Limeted differences between references (<1% identity difference)
iBS90_Bu and iM_Zhong
iM_Zhong and Bg2024
Bg2024 and iBS90_Bu
Reference genes are too fragmented
Match between resistant. 1316R unclear ancestry
Match between susceptible. 1316R unclear ancestry
