# Supplementary material for: The growing repertoire of putative pathogen interaction genes in the Biomphalaria glabrata vs Schistosoma mansoni system
Source: PLoS Negl Trop Dis. 2026 Oct 21;20(10):e0014803. doi: 10.1371/journal.pntd.0014803 (PMC13712769; doi:10.1371/journal.pntd.0014803)
Supplement: Supplementary file 4 [file pntd.0014803.s004.pdf]

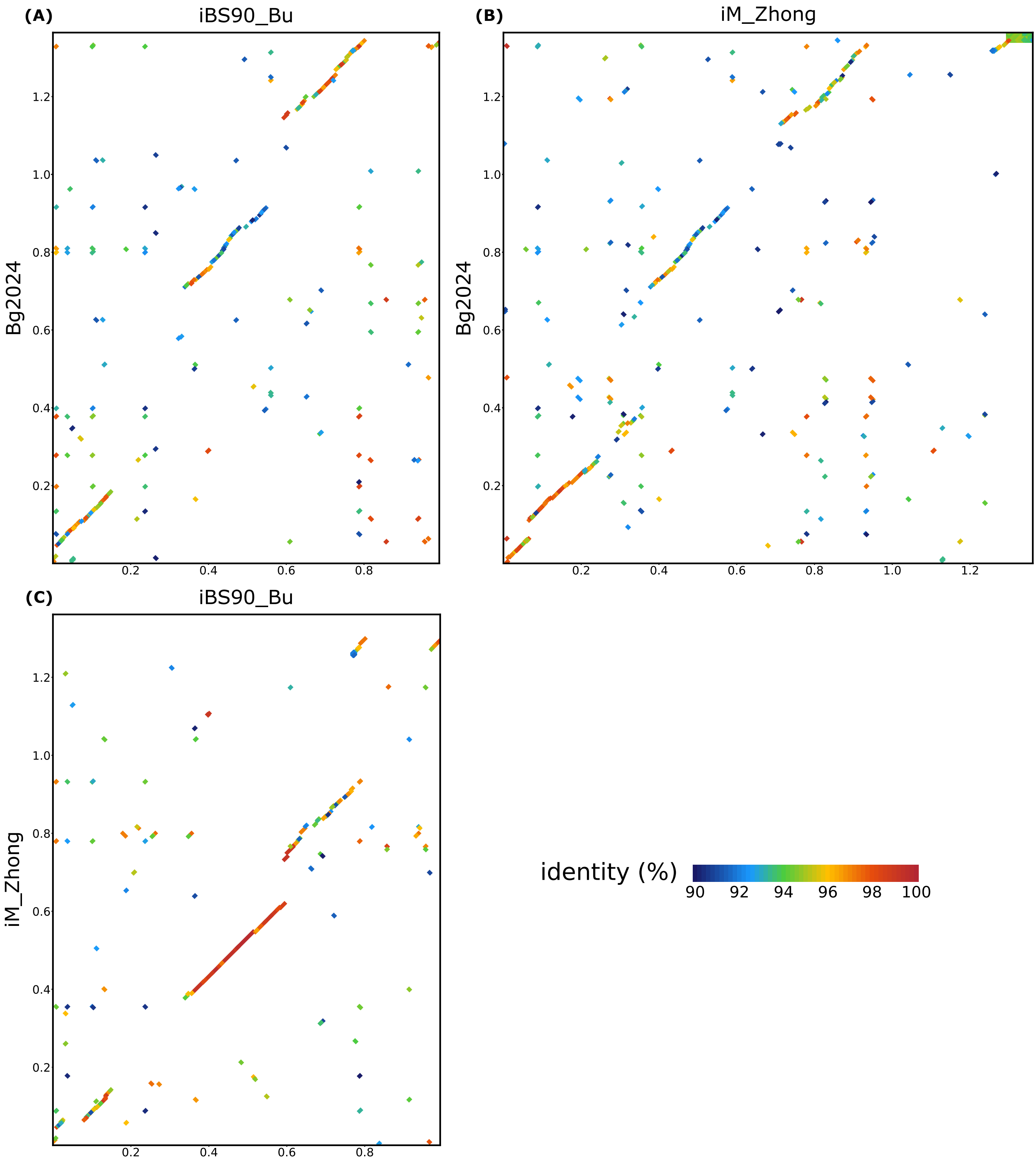
(A)
iBS90_Bu
Bg2024
(B)
iM_Zhong
Bg2024
(C)
iBS90_Bu
iM_Zhong
0.2
0.4
0.6
0.8
1.0
1.2
identity (%)
90
92
94
96
98
100
