# Supplementary material for: The growing repertoire of putative pathogen interaction genes in the Biomphalaria glabrata vs Schistosoma mansoni system
Source: PLoS Negl Trop Dis. 2026 Oct 21;20(10):e0014803. doi: 10.1371/journal.pntd.0014803 (PMC13712769; doi:10.1371/journal.pntd.0014803)
Supplement: Supplementary file 5 [file pntd.0014803.s005.pdf]

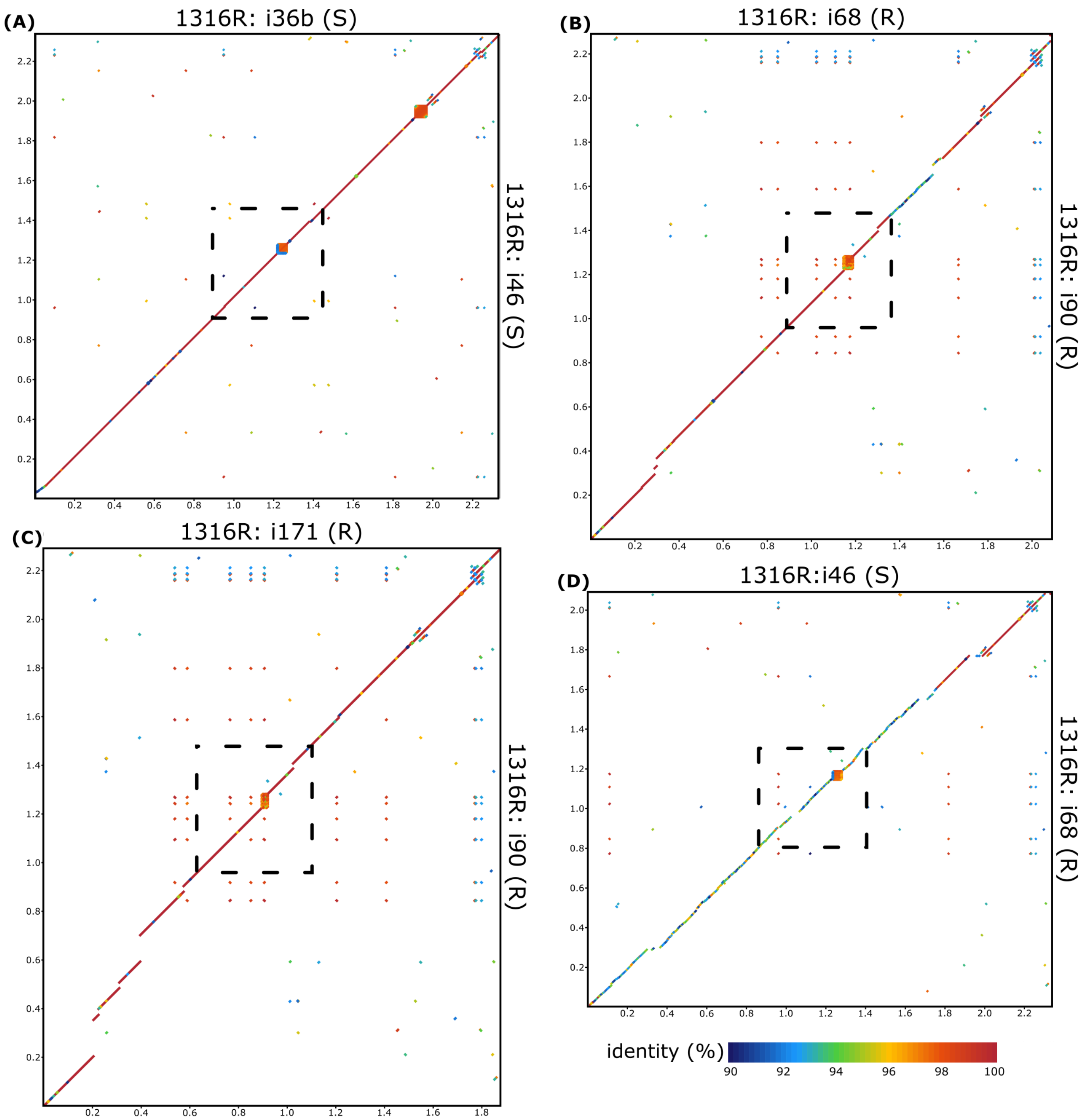
(A)
1316R: i36b (S)
1316R: i46 (S)
(B)
1316R: i68 (R)
1316R: i90 (R)
(C)
1316R: i171 (R)
1316R: i90 (R)
(D)
1316R:i46 (S)
1316R: i68 (R)
identity (%)
90
92
94
96
98
100
