# Supplementary material for: The growing repertoire of putative pathogen interaction genes in the Biomphalaria glabrata vs Schistosoma mansoni system
Source: PLoS Negl Trop Dis. 2026 Oct 21;20(10):e0014803. doi: 10.1371/journal.pntd.0014803 (PMC13712769; doi:10.1371/journal.pntd.0014803)
Supplement: Supplementary file 6 [file pntd.0014803.s006.pdf]

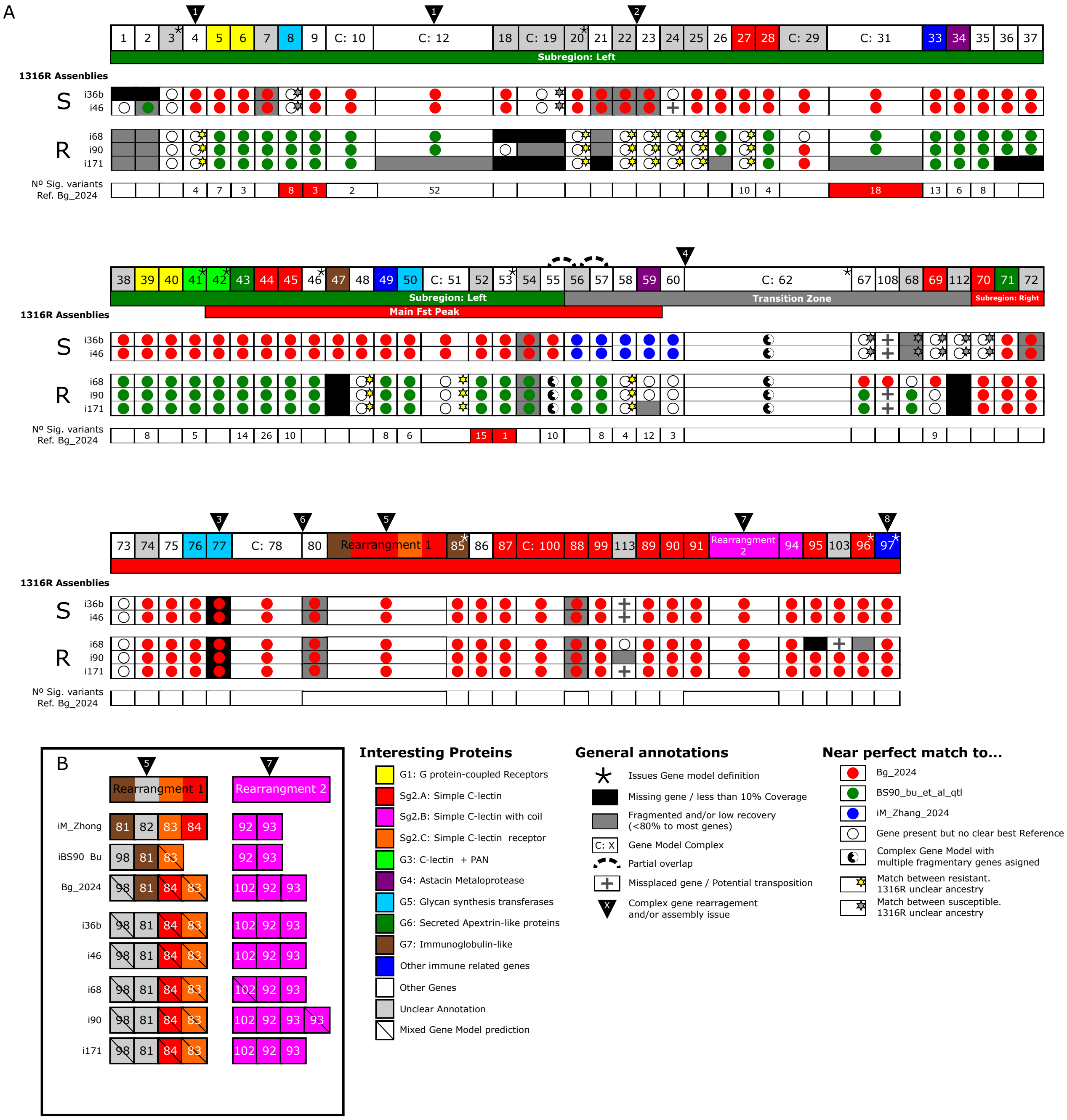
A
1316R Assenblies
S
i36b
i46
R
i68
i90
i171
Nº Sig. variants
Ref. Bg_2024
Subregion: Left
Main Fst Peak
Transition Zone
Subregion: Right
Rearrangment 1
Rearrangment 2
B
iM_Zhong
iBS90_Bu
Bg_2024
Interesting Proteins
G1: G protein-coupled Receptors
Sg2.A: Simple C-lectin
Sg2.B: Simple C-lectin with coil
Sg2.C: Simple C-lectin receptor
G3: C-lectin + PAN
G4: Astacin Metaloprotease
G5: Glycan synthesis transferases
G6: Secreted Apextrin-like proteins
G7: Immunoglobulin-like
Other immune related genes
Other Genes
Unclear Annotation
Mixed Gene Model prediction
General annotations
Issues Gene model definition
Missing gene / less than 10% Coverage
Fragmented and/or low recovery (<80% to most genes)
Gene Model Complex
Partial overlap
Missplaced gene / Potential transposition
Complex gene rearragement and/or assembly issue
Near perfect match to...
Bg_2024
BS90_bu_et_al_qtl
iM_Zhang_2024
Gene present but no clear best Reference
Complex Gene Model with multiple fragmentary genes asigned
Match between resistant. 1316R unclear ancestry
Match between susceptible. 1316R unclear ancestry
