# Supplementary material for: The growing repertoire of putative pathogen interaction genes in the Biomphalaria glabrata vs Schistosoma mansoni system
Source: PLoS Negl Trop Dis. 2026 Oct 21;20(10):e0014803. doi: 10.1371/journal.pntd.0014803 (PMC13712769; doi:10.1371/journal.pntd.0014803)
Supplement: Supplementary file 7 [file pntd.0014803.s025.pdf]

Supplemental data 1:

Identifying significant Fst peaks in our PoolSeq dataset:

We chose Fst > 0.04 as the cutoff for significance in the initial comparison for three reasons.

(1) Fst ~ 0.04 is the height of peaks around known resistance loci in the SOD and RADres regions, which also stood out as well above the genome-wide background Fst (Fig. 1 in Tennessen et al., 2020).

(2) In simulations of unlinked loci with minor allele frequencies ≥ 0.2, with copies randomly assigned to 600 infected and 600 uninfected individuals, and with 500x coverage, the probability of a locus having Fst ≥ 0.04 by chance alone equaled 0.0001 (Github script SimulatePools.pl at https://github.com/jacobtennessen/GOPOPS/).

(3) To run the original pools of N = 600, we made two, individually barcoded libraries of N = 300 each. Thus, we have two ways we can bioinformatically mix and match libraries to compare two pools of N = 600 each, in which each pool is a 50:50 mix of infected and uninfected snails. In neither comparison did any individual window exceed Fst = 0.031, and well-formed peaks were well below that (Fig. 1 below).

Artifact Peak on Chromosome 11

In examination of the sliding window plot for Chromosome 11 using 5 SNPs per window cutoff, we see a potential peak in the middle of the chromosome that did not meet criteria at 150 SNPs per window. However, that region also shows an increase in Fst in the two 50:50 mixture comparisons, including one window with Fst > 0.06 (Fig. 2 below). It is also a region of low read coverage and very low identity between haplotypes, so we were not confident in calling that middle peak a true region of association with the resistance phenotype.

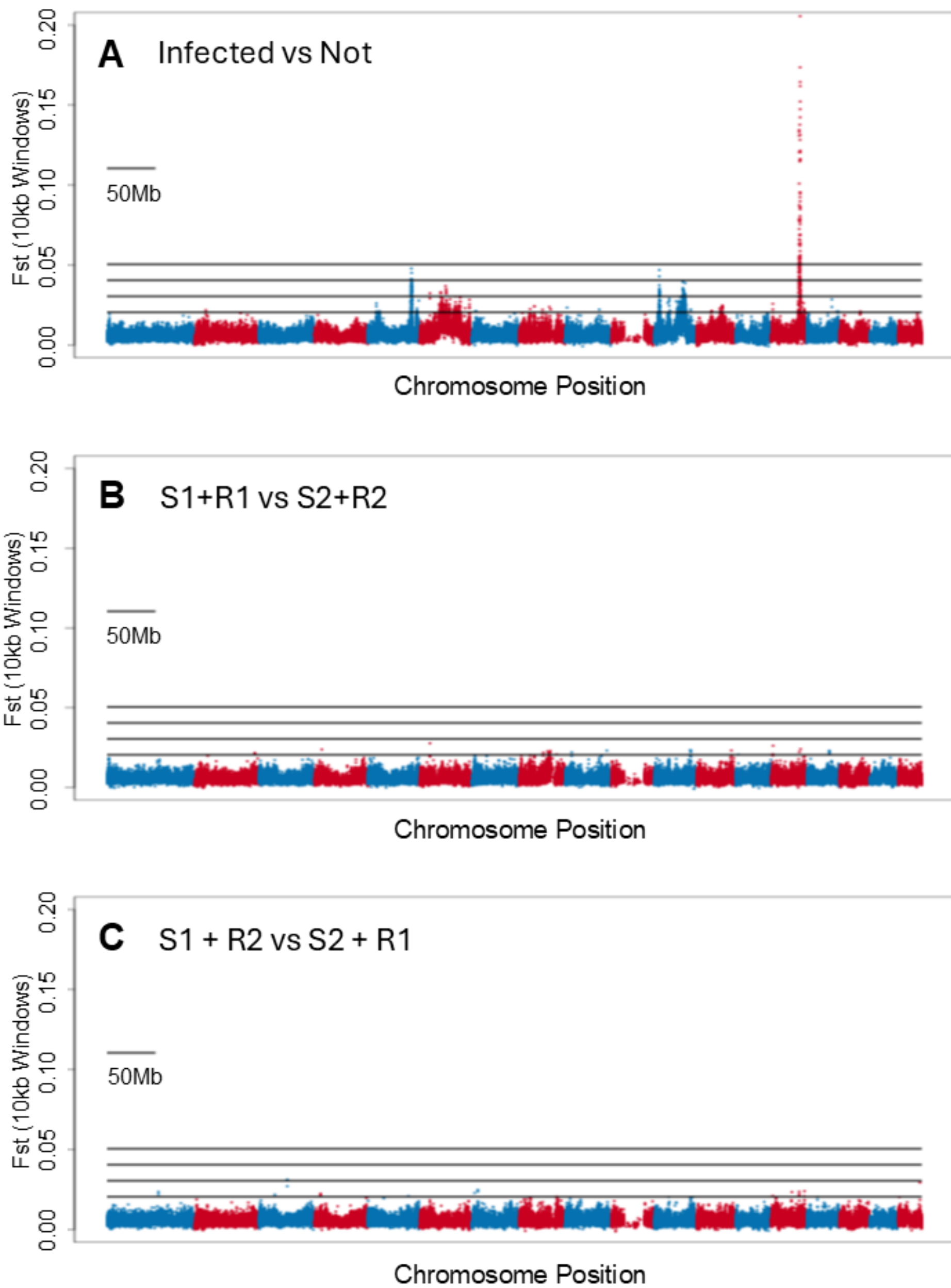

Fig. 1. Sliding window Fst (same methods as in main manuscript, minimum of 150 SNPs per window) on original data and on two pools of N = 600 each that each consist of DNA from 300 infected and 300 uninfected snails. The two libraries from the infected group are S1 and S2, and the two libraries from the uninfected group are R1 and R2. Panel A (Infected vs Not) shows the original comparison between the 600 infected and 600 non-infected groups (same as figure 1a in the main manuscript). Panel B shows artificial groups S1+R1 vs S2+R2. Panel C shows artificial groups S1 + R2 vs S2 + R1. Horizontal lines show Fst cutoffs from 0.02 to 0.05 in increments of 0.01. The highest Fst window in the comparisons between artificial groups = 0.031.

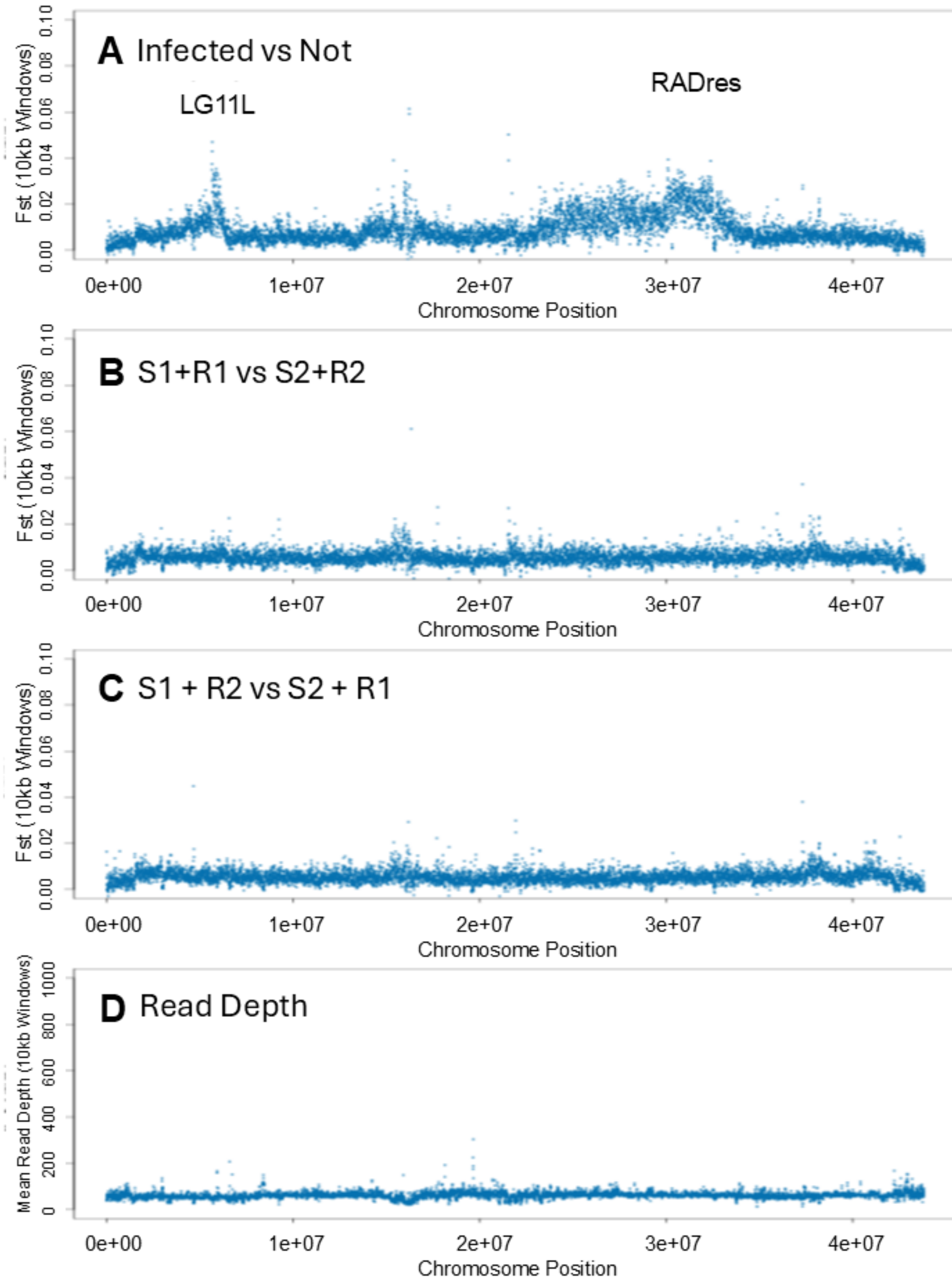

Fig 2. Sliding window Fst for chromosome 11 (same methods as in main manuscript, minimum of 5 SNPs per window), for the original comparison, and for the two artificial of pools N = 600 each that each consist of DNA from 300 infected and 300 uninfected snails. The two libraries from the infected group are S1 and S2, and the two libraries from the uninfected group are R1 and R2. Panel (A) shows comparison between the original infected and non-infected groups (same as figure 1C in the main manuscript). (B) group S1+R1 vs group S2+R2. (C) group S1 + R2 vs group S2 + R1. The middle peak shows elevated Fst in both of the artificial mixture comparisons (panels B and C), plus a dip in read depth relative to the rest of the chromosome (Panel D). Thus, we believe that peak is an artifact unrelated to schistosome resistance.
