# Supplementary material for: The growing repertoire of putative pathogen interaction genes in the Biomphalaria glabrata vs Schistosoma mansoni system
Source: PLoS Negl Trop Dis. 2026 Oct 21;20(10):e0014803. doi: 10.1371/journal.pntd.0014803 (PMC13712769; doi:10.1371/journal.pntd.0014803)
Supplement: Supplementary file 8 [file pntd.0014803.s026.pdf]

Supplemental Data 2:

Notes on discrepancies in the gene composition found among different assemblies inside the regions of association LG11L and LG5R. The causes of the discrepancies are likely a combination of biological factors (e.g. large structural variants) and artifacts of various causes (e.g. lack of sequence in the assembly forces liftoff to identify the wrong paralog to the gene or report a secondary best hit).

LG11L

Case 1: Inconsistent location of Gene_Model_15

Gene model is defined by the location of 3 genes: BgiBS90_020574 (original annotation: iBS90_Bu), BgiMline_021585 (original annotation: iM_Bu) and BgiMline_025496 (original annotation: iM_Zhong). As shown in **Fig_Case1**, all 3 genes are located within one of the introns of Gene_Model_12 on iM_Zhong, but they are found on Gene_Model_4 on i171 and both on Bg2024. Considering the mismatch and overall low identity of these sequences reported by Liftoff the more likely explanation is that they represent erroneous annotations of different paralogs. Potentially of a lncRNA given the lack of reliable annotations.

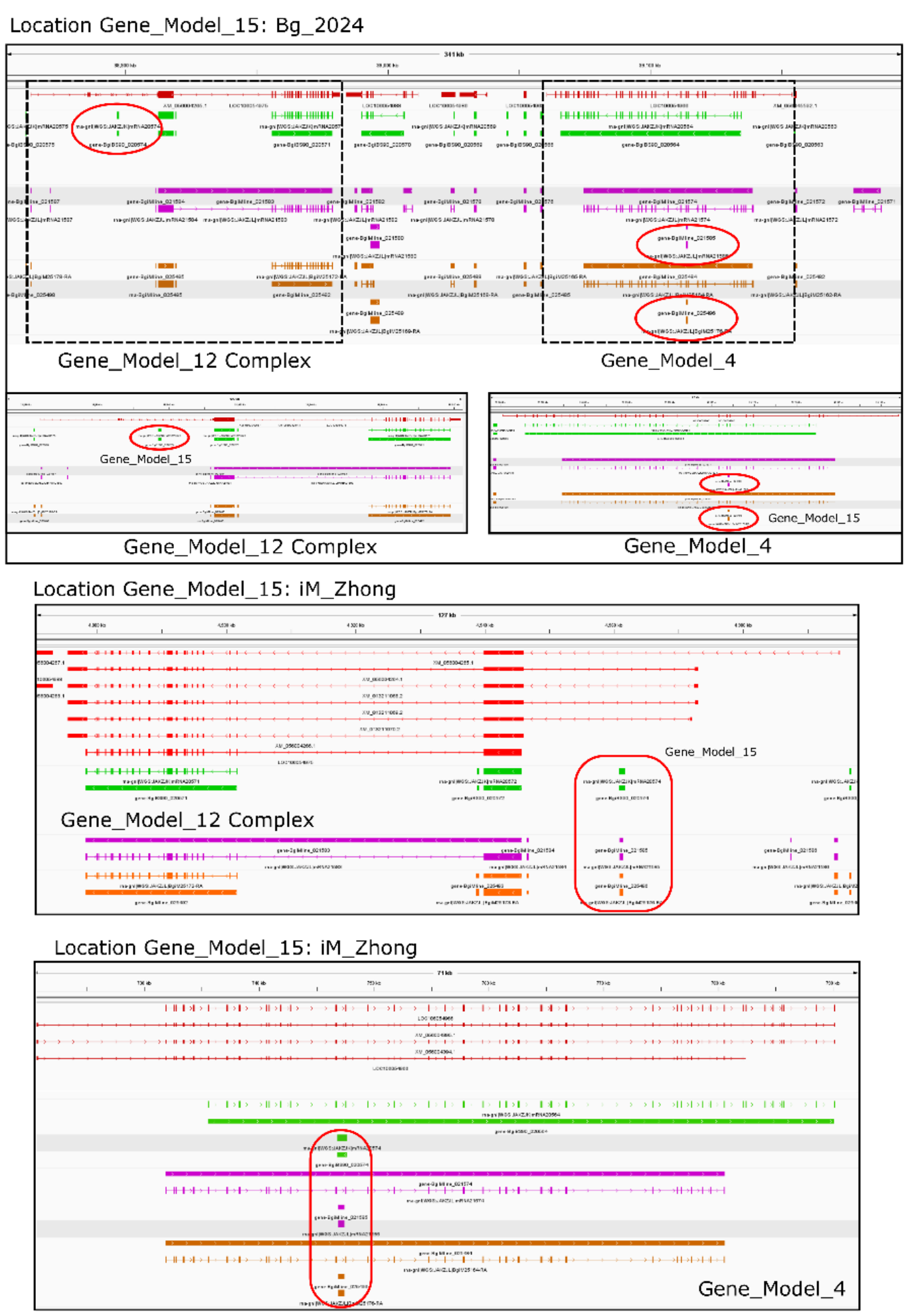

Fig_Case1: Sketches displaying the genome annotations detailing the location of Gene_Model_15 (red oval) for the assemblies iM_Zhang_2024, 1316_S2_i171 and Bg2024. Colored tracks represent genes from different sources: Bg2024 (red), iBS90_Bu (green), iM_Bu (purple) and iM_Zhong (orange).

Case 2: Overlap between Gene_Models_22 and _23

The Gene_Model _22 and _23, defined by genes BgiBS90_020578 and BgiBS90_020579 (original annotation: iM_Bu) respectively, are encoded in proximity but opposite strands in iM_Bu. On the references iM_Zhong and Bg2024 however they display a considerable overlap and a potential loss of function of Gene_Model_23 on Bg2024 given the gain of several stop codons (**Fig_Case2**). It remains unclear however if this is the case since the genes could not be properly annotated in the first place and Liftoff reports a considerable loss of gene coverage in both assemblies.

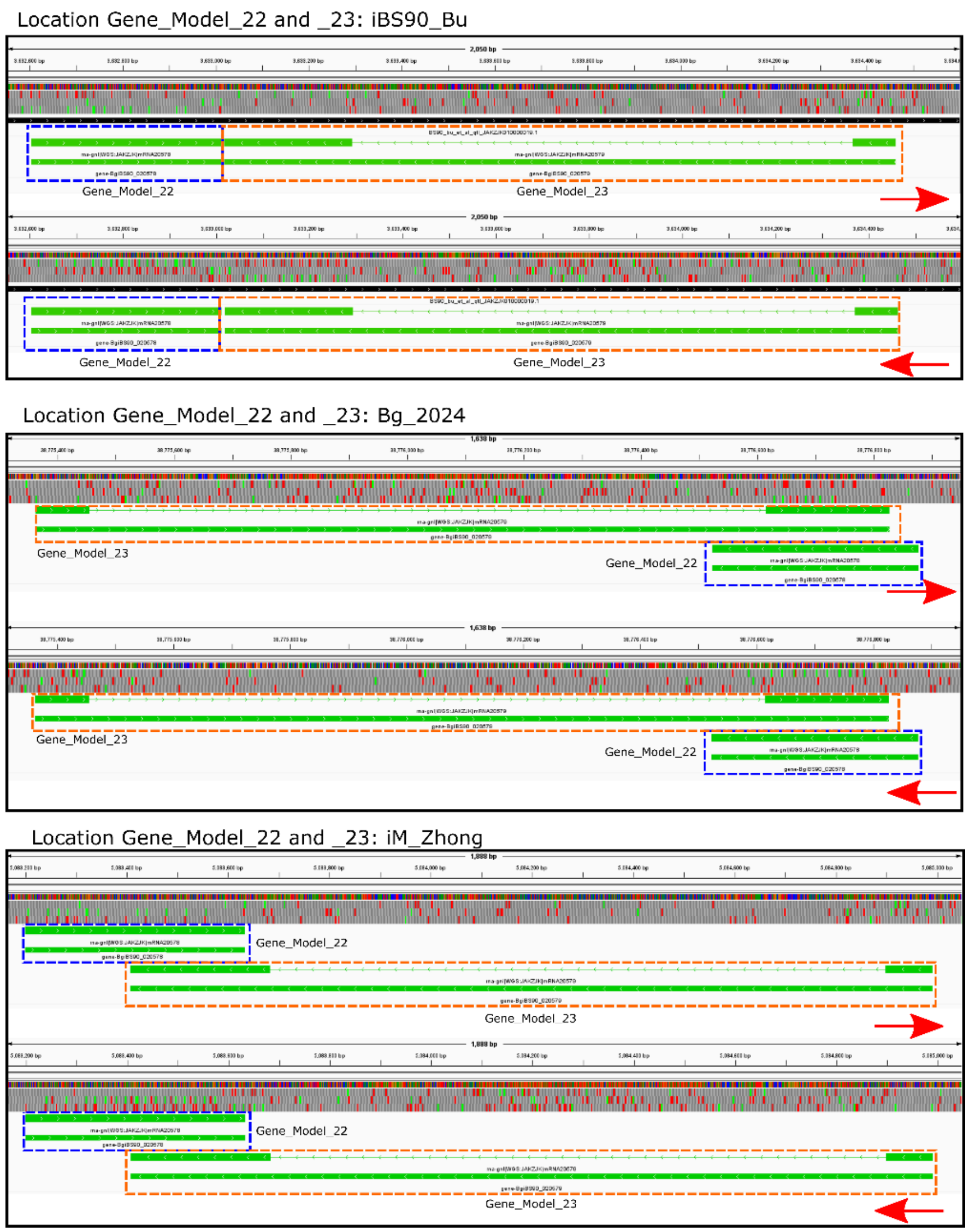

Fig_Case2: Sketches displaying the genome annotations detailing the location of Gene_Model_22 (small blue box) and _23 (large orange box) for assemblies iBS90_Bu, iM_Zhong, and Bg2024. Green tracks represent the gene annotations coming from the iBS90_Bu genome. Above them is a representation of the predicted open

reading frames with green marking methionine, red stop codons and red any other AA. The red arrow shows the orientation of the translation (plus or minus).

Case 3: Gain/Loss Gene_Model_77

Gene models Gene_Model_76 and _77 encode for paralog copies of “alpha-(1 3)-fucosyltransferase C” on the iM_Zhong and iBS90_Bu assemblies. However, on Bg2024 and all 1316Rs Gene_Model_77 is missing and as result BgiBS90_020642 (original annotation: iBS90_Bu) is placed within Gene_Model_76 by Liftoff (**Fig_Case3)** with low identity and coverage metrics (~35% and ~37% respectively).

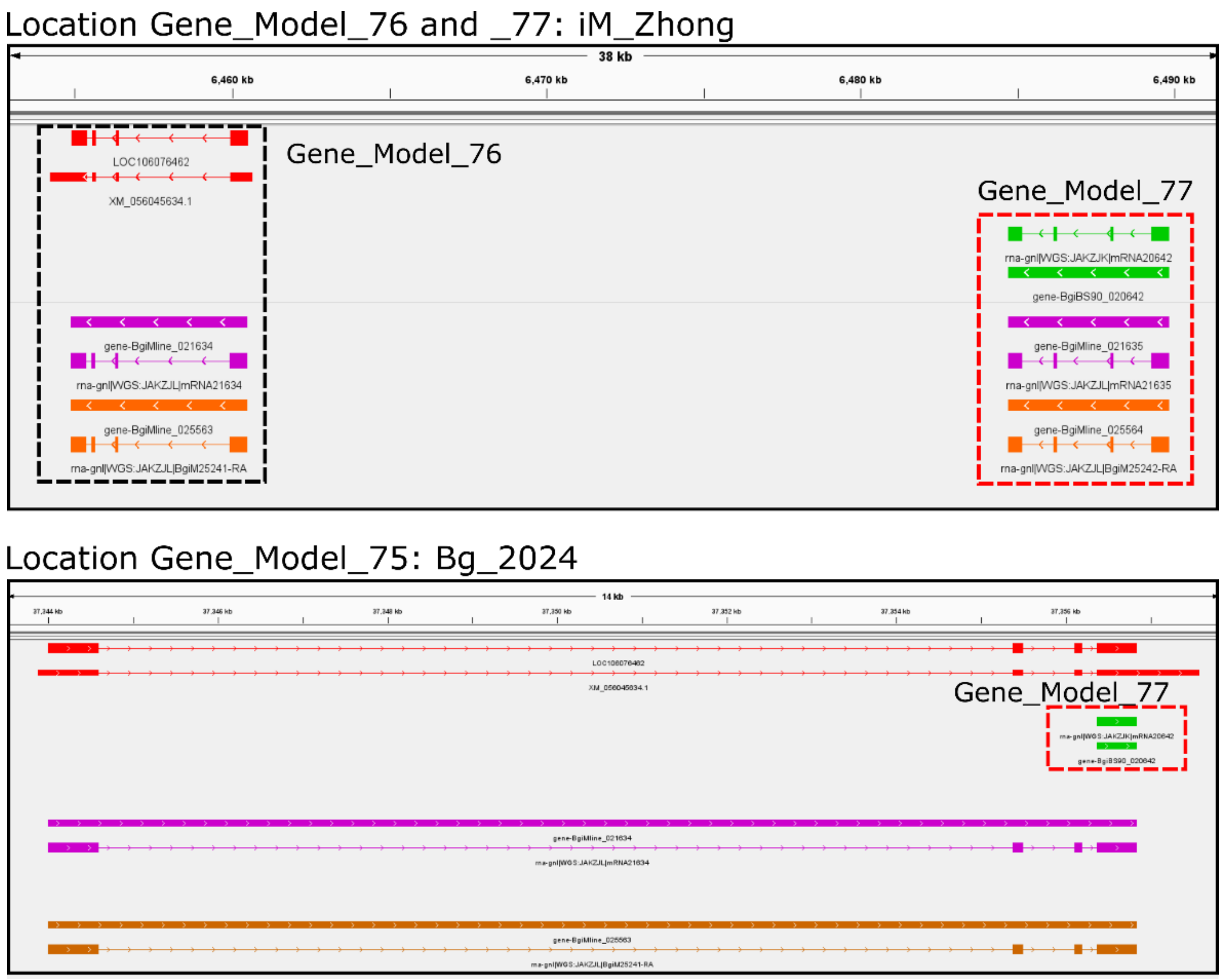

Fig_Case3: Sketches displaying the location of Gene_Model_76 (black box) and _77 (black box) on iM_Zhang_2024 and Bg2024. Colored tracks represent genes from different sources: Bg2024 (red), iBS90_Bu (green), iM_Bu (purple) and iM_Zhong (orange). Gene_Model_77 is missing on the Bg2024 genome assembly and gene BgiBS90_020642 (original annotation: iBS90_Bu) is assigned to Gene_Model_76 instead.

Case 4: Inconsistent location of Gene_Model_61

Gene_Model_61 is defined by the extra copies of gene BgiBS90_023561 identified by Liftoff's cluster mode. The original gene is a short protein of 56AA and has no domain signatures. The position of this gene varies across assemblies and can be found within the introns Gene_Model_60 in iM_Zhong and i36b; and within Gene_Model_66: BS90_bu_et_al_qtl (**Fig_Case4**).

Location Gene_Model_61: iM_Zhong

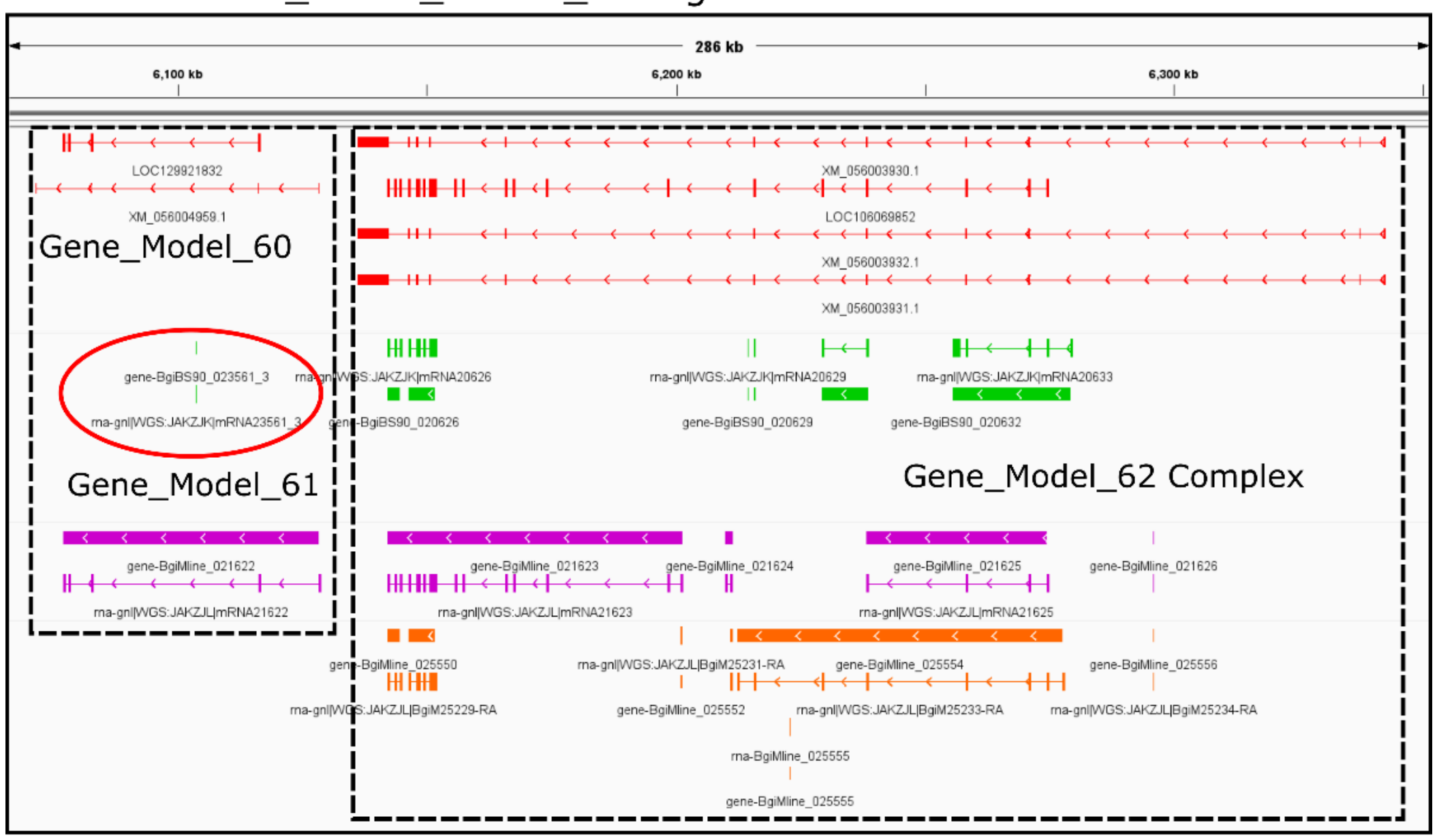

Location Gene_Model_61: iM_Bu

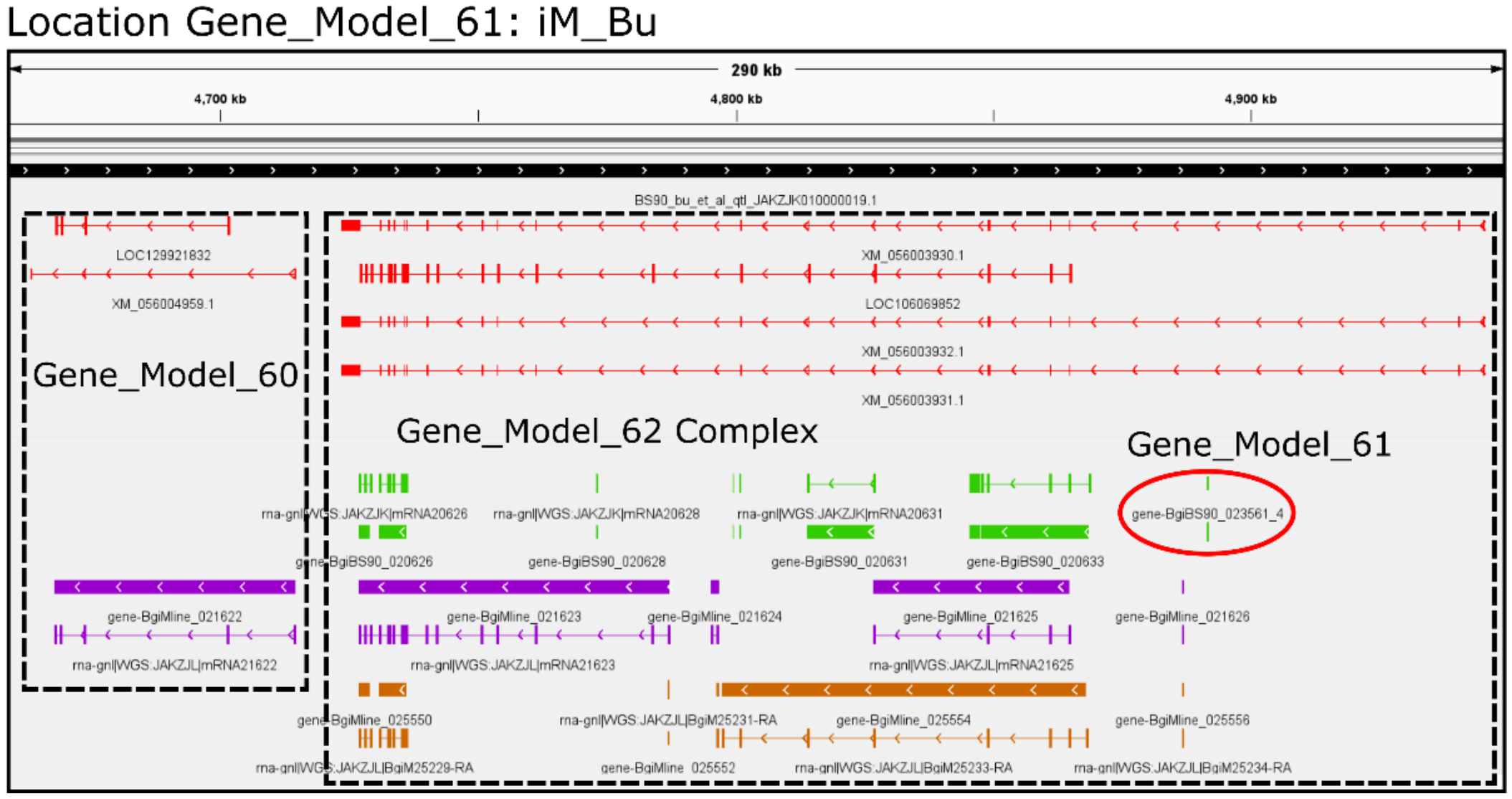

Fig_Case4: Sketches displaying the location of Gene_Model_61 (red oval) on assemblies iM_Zhong and BS90_bu_et_al_qtl. Colored tracks represent genes from different sources: Bg2024 (red), iBS90_Bu (green),

iM_Bu (purple) and iM_Zhong (orange). In both cases the location of Gene_Model_61 is dependent on copies of gene BgiBS90_023561 (original annotation: iM_Bu), identified by Liftoff cluster Mode.

Case 5: Evidence of re-arrangements within the C-lectin gene cluster – Reference assemblies

This is the First complex re-arrangement of genes found within the simple C-lectin rich conglomeration found to the right of the Fst peak, as displayed in the main manuscript (upstream on iM_Zhong and iBS90_Bu). All 3 reference genomes display different haplotypes with different gene composition and order. For simplicity this is spread in two subcases: 7a showing the transposition of Gene_Model_98 (gene: LOC106050975), 7b showing additional alterations upstream, and 7c showing rearrangements downstream.

Subcase 5a (**Fig_Case5a**):

- iM_Zhong has a relatively large intergenic space between Gene_Model_80 and _81, followed by Gene Model 82.
- iBS90_Bu shows a transposition of Gene_Model_98 between Gene_Model_80 and _81, accompanied with the loss of the first exons of the main gene signatures for Gene_Model_81. In this assembly the Gene_Model_78 Complex extends to include Gene Model 80 (Alterations described in Subcase 7b).
- Bg2024 has the same transposition as described in BS90_bu_et_al_qtl, but the alterations to Gene_Model_81 are more severe. Furthermore, gene BgiMline_025574 from Gene_Model_85 overlaps with Gene_Model_98, suggesting further alterations.

Subcase 5b (**Fig_Case5b**):

- iM_Zhong displays Gene_Model_80 is located immediately upstream of the Gene_Model_78 complex, that in this assembly only includes Gene_Model_79
- iBS90_Bu shows a split Gene_Model_80 with some gene signatures located within the Gene_Model_78 complex, that also incorporates Gene_Model_107.
- Bg2024 is similar to iM_Zhang_2024 with a non-split Gene_Model_80, but the Gene_Model_78 complex includes Gene_Model_107 and Gene_Model_79 is severely reduced.

Subcase 5c (**Fig_Case5c**):

- iM_Zhong has Gene_Model_82 to _84 clearly defined, with a lncRNA between _82 and _83 that was not included in the analysis because it is exclusive of this genome as part of LG10_Left (ID: LOC129921969, transferred from Bg2024).
- iBS90_Bu lacks Gene_Model_82 and _84. Some elements of the latter were found within Gene_Model_83.
- Bg2024 lacks Gene_Model_82, while Gene_Model_83 and Gene_Model_84 switch places without altering their strand orientation relative to Gene_Model_81 and _85

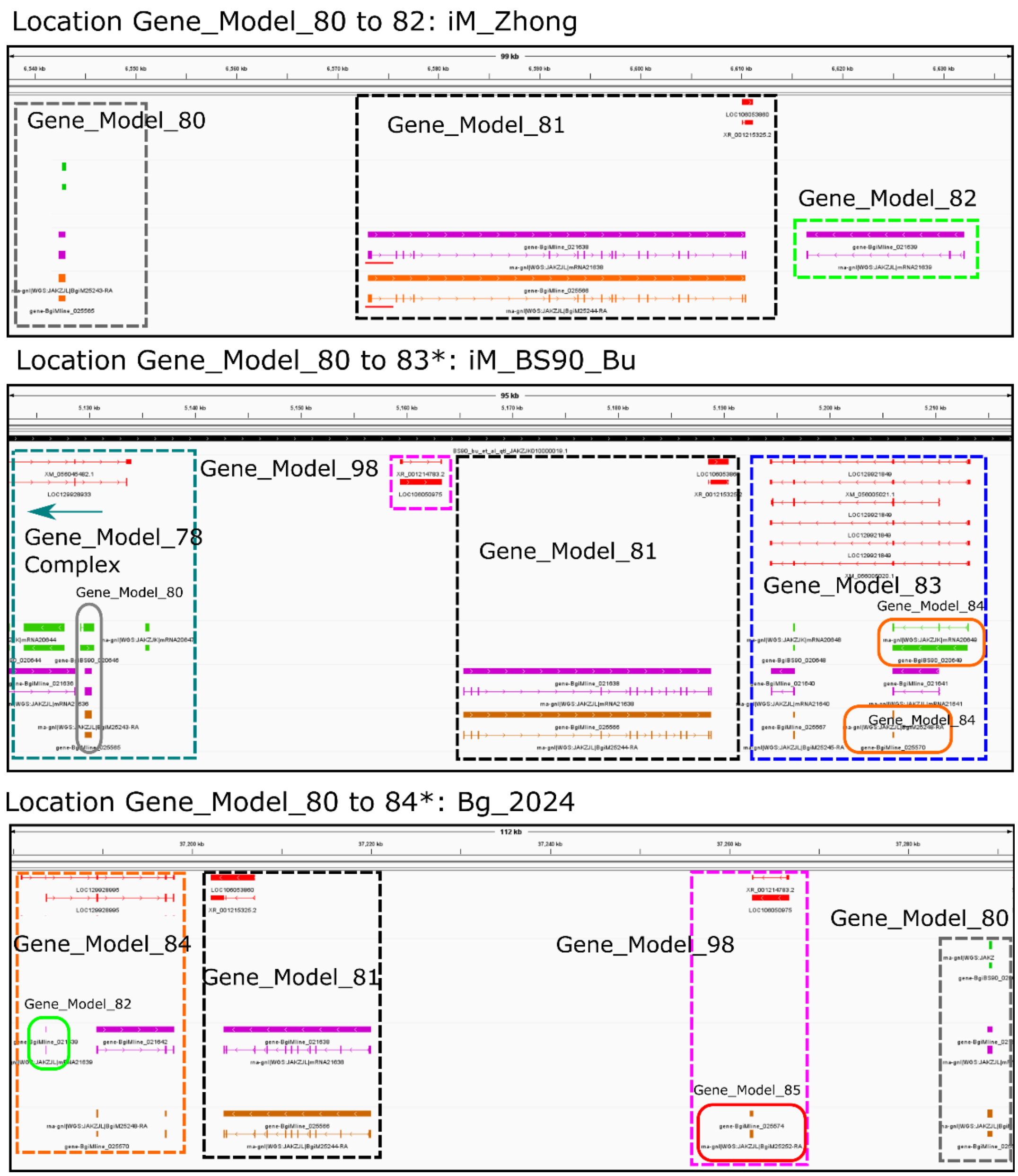

Fig_Case5a: Sketches displaying the location of Gene_Model_78 (teal box) _80 (grey box), _81 (black box), _82 (green box), _83 (blue box) and _84 (orange box) on assemblies iM_Zhong, iBS90_Bu and Bg2024. Colored tracks represent genes from different sources: Bg2024 (red), iBS90_Bu (green), iM_Bu (purple) and iM_Zhong (orange). Genes originally described as members of one gene model but annotated within another are marked with boxes with continuous borders and colored according to their original designation. Among them a gene signature for Gene_Model_85 is marked in red.

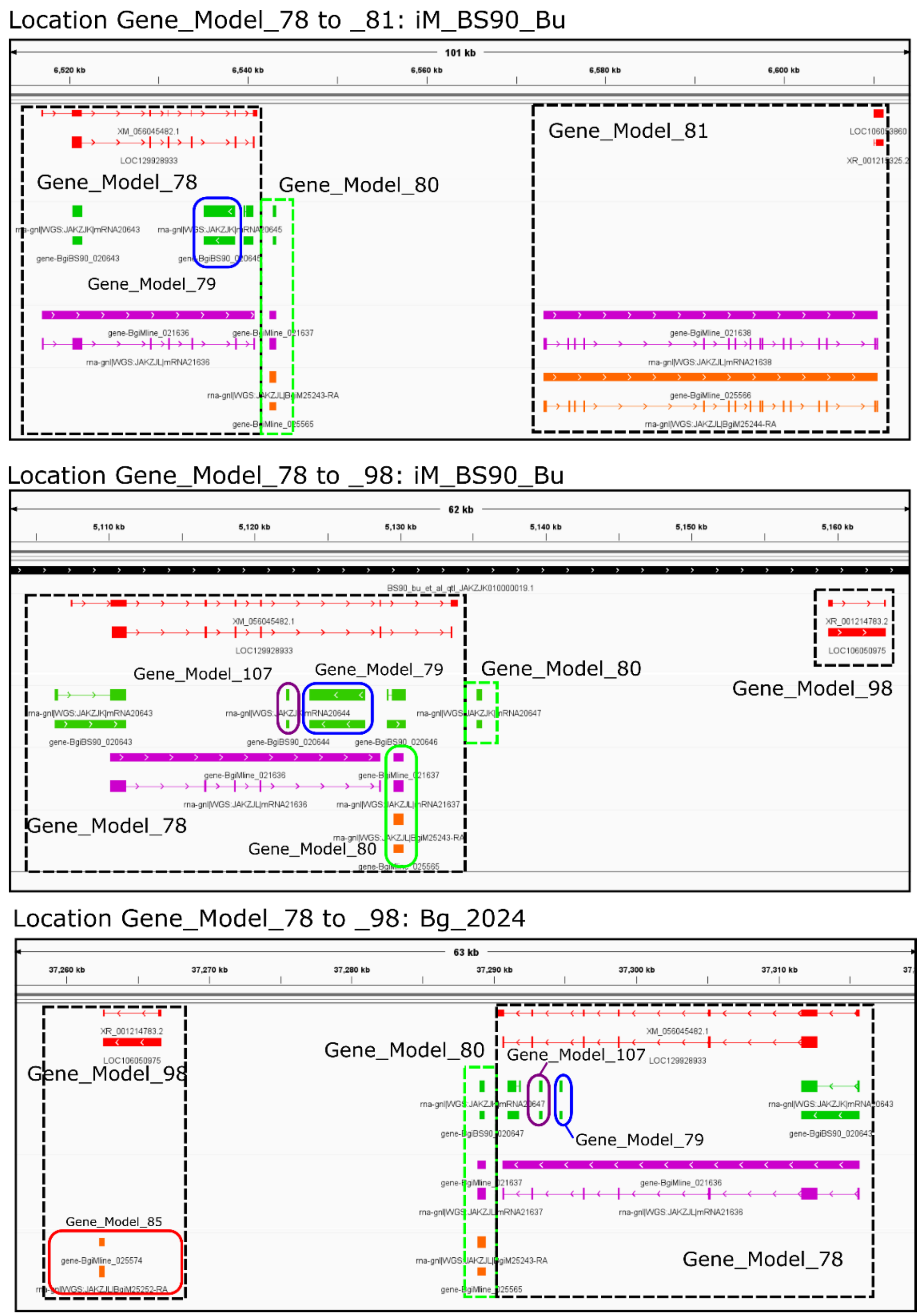

Fig_Case5b: Sketches displaying the location of Gene_Model_78, _81 and _98 (black box) the gene model discussed in this subsection: Gene_Model_79 (blue box), Gene_Model_80 (green box) and Gene_Model_107

(purple box). Plus, Gene_Model_85 (red box) that is better explained in subcase 7b. Colored tracks represent genes from different sources: Bg2024 (red), iBS90_Bu (green), iM_Bu (purple) and iM_Zhong (orange).

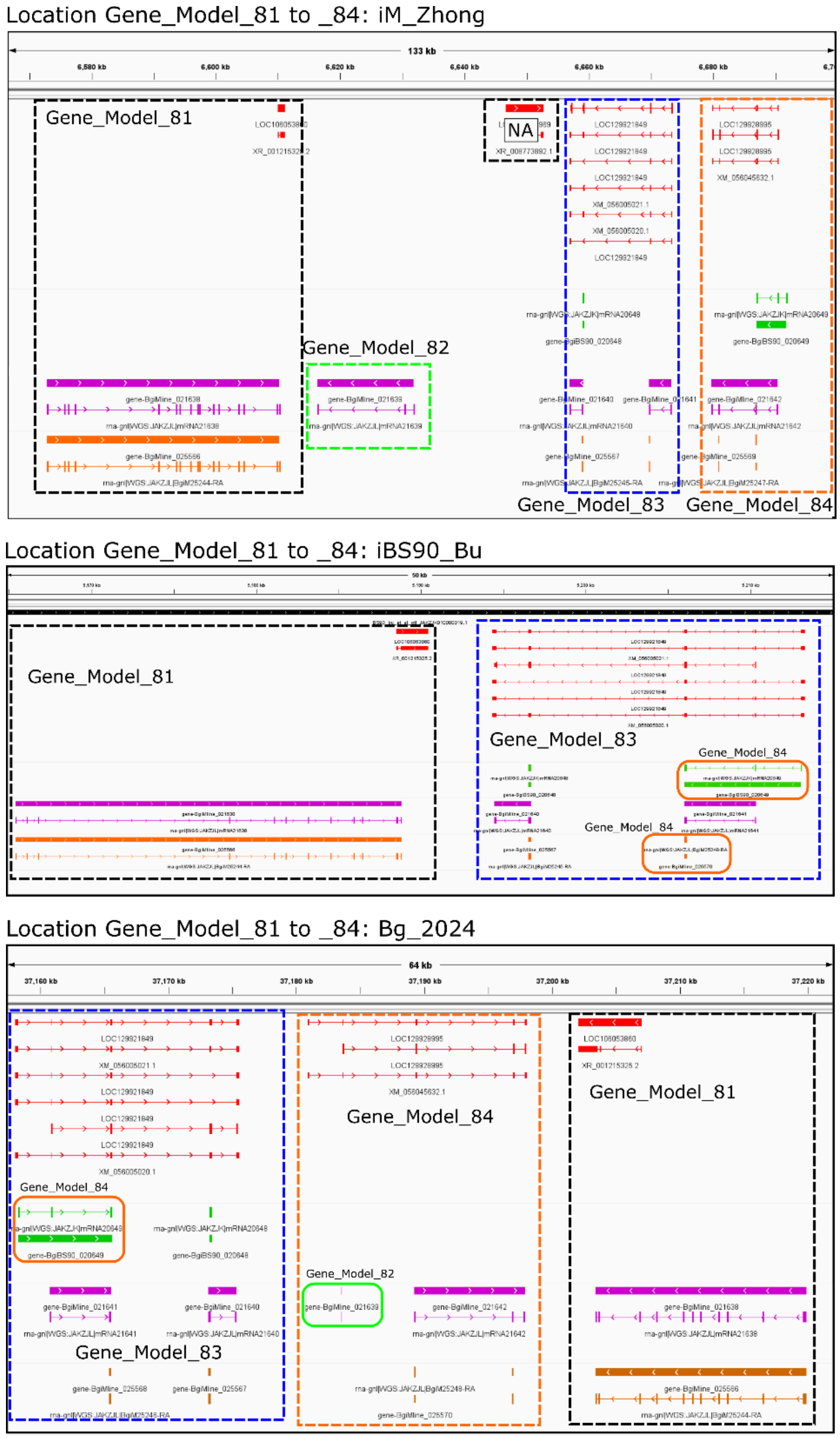

Fig_Case5c: Sketches displaying the location of Gene_Model_81 (black box), _82 (green box), _83 (blue box) and _84 (orange box) on assemblies iM_Zhong, iBS90_Bu and Bg2024. Colored tracks represent genes from different sources: Bg2024 (red), iBS90_Bu (green), iM_Bu (purple) and iM_Zhong (orange). Genes originally

described as members of one gene model but annotated within another are marked with boxes with continuous borders and colored according to their original designation.

Case 6: Evidence of re-arrangements within the C-lectin gene cluster – 1316R

The 1316R assemblies i68, i90 and i171 are derivates of Bg2024 for the loci configurations showcased in Case 7a and 7b, with an additional deletion of approximately ~50kb deletion between Gene_Model_81 and _98 that eliminates the remains of gene BgiMline_025566 (iM_Zhong) / BgiMline_021638 (original annotation: iM_Bu) (**Fig_Case6**). Likely as result, they are re-assigned as part of Gene_Model_85 as partial detections.

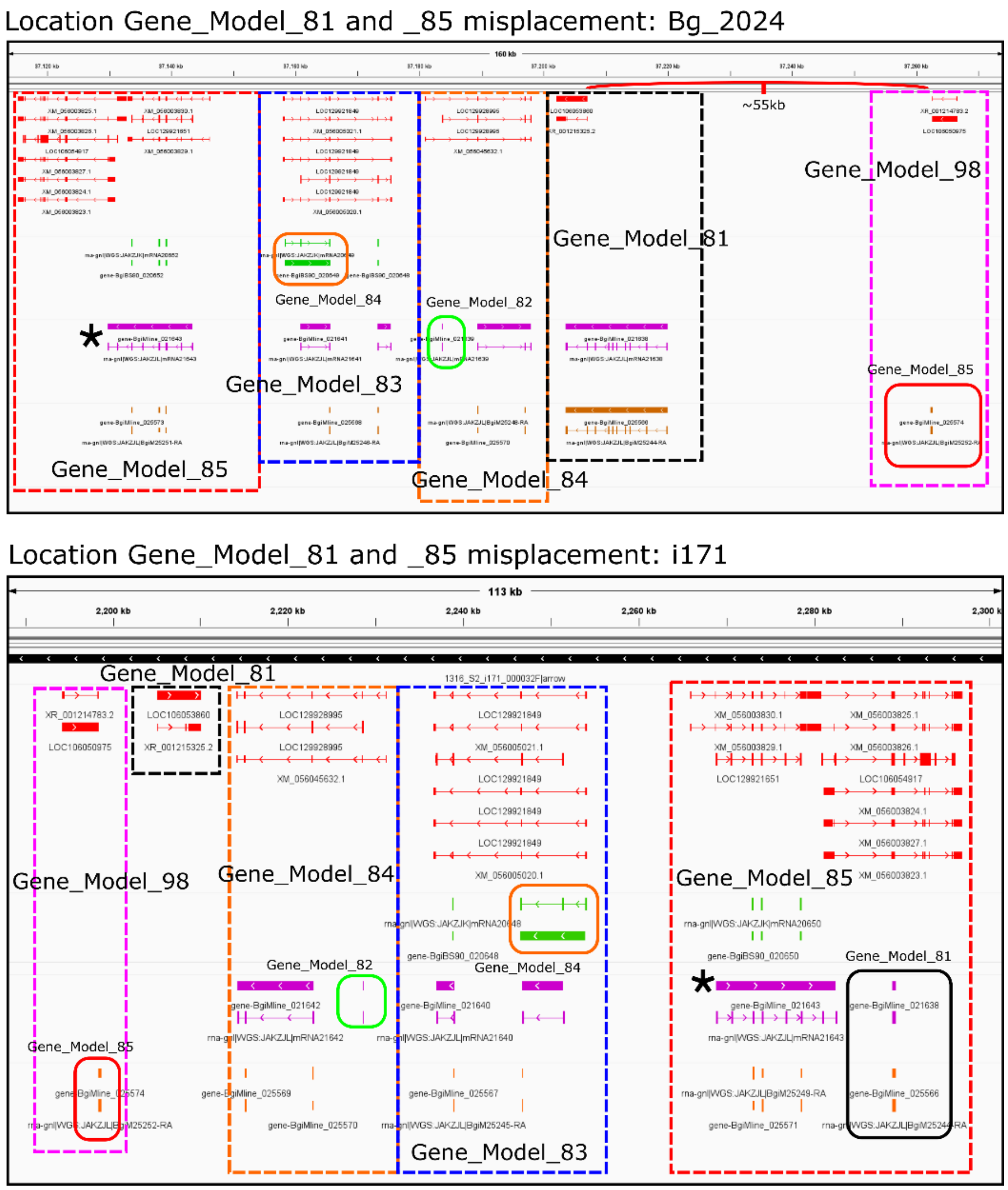

Fig_Case6: Sketches displaying the location of Gene_Model_81 (black box), _82 (green box), _83 (blue box), _84 (orange box) and _98 (purple box) on assemblies Bg2024 and i171. Genes originally described as members of one gene model but annotated within another are marked with boxes with continuous borders and colored according to their original designation and are further described in Case7 and 8. Colored tracks represent genes from different sources: Bg2024 (red), iBS90_Bu (green), iM_Bu (purple) and iM_Zhong

(orange). The deletion observed between Bg2024 and the 1316R assemblies is shown with an arc on the Bg2024 diagram. Gene_Model_85 is considered a potential chimera generated by our methodology due to gene BgiMline_021643 (original annotation: iM_Bu) serving as a bridge between 2 sets of overlapping genes (marked with an asterisk).

Case 7: Internal changes between assemblies on the C-lectin gene cluster

Upstream to the previous cases, the C-lectin cluster stabilizes until reaching Gene_Model_91. Here Bg2024 displays an indel relative to the other genomic references with the addition of Gene_Model_102 between 91 and 92. The 1316R assemblies show the same composition with only 2 exceptions:

- i90 shows a duplication of Gene_Model_92 (identified only by gene LOC106054921), that splits the genes from Gene_Model_102.
- i68 has Gene_Model_96 and _97 clustered with Gene_Model_102. But this is likely an artifact due to lost information forcing liftoff to relocate them into a different paralog (see **Case 8**).

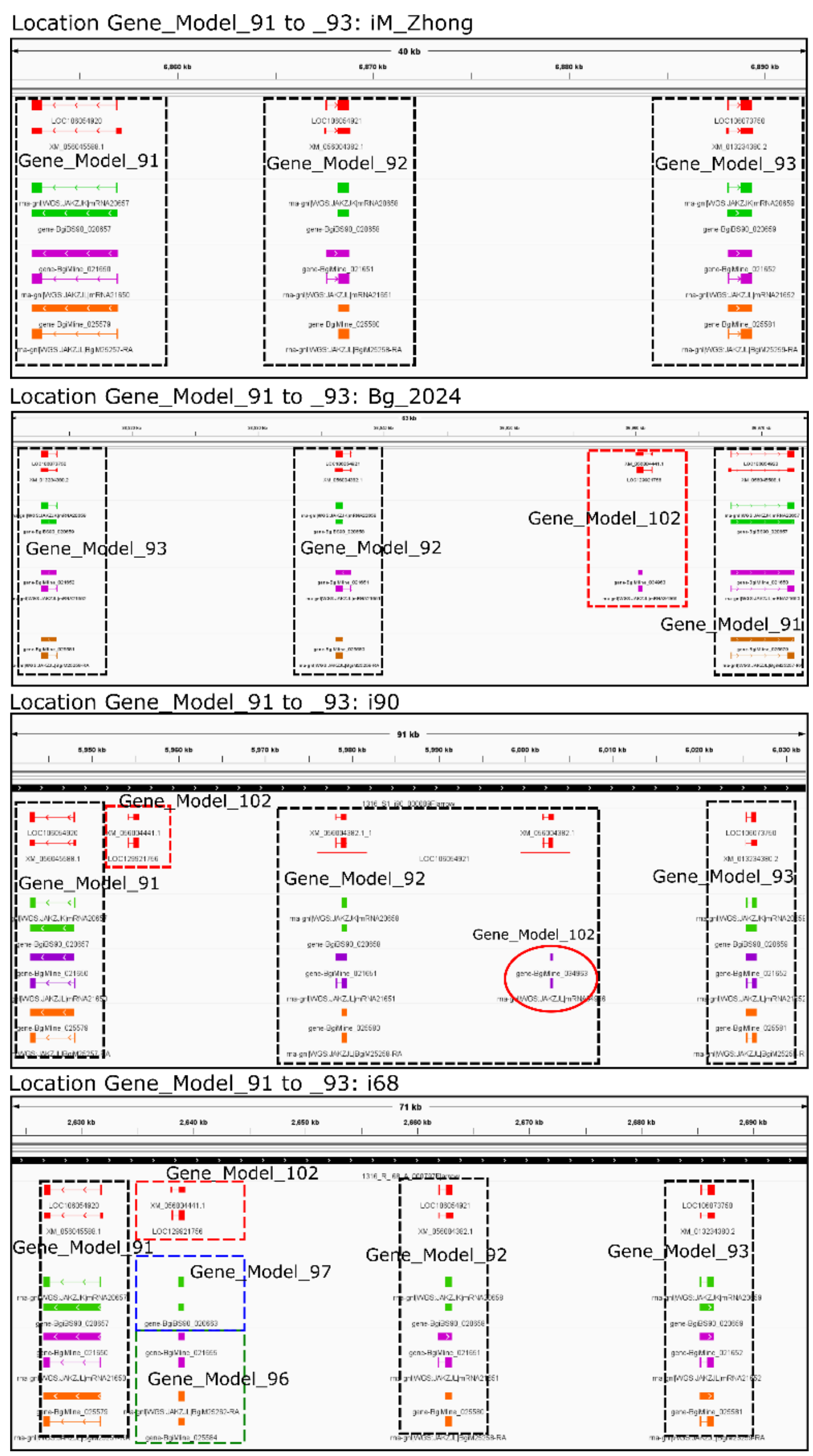

Fig_Case7: Sketches displaying the location of the somewhat conserved Gene_Model_91, _92 and _93 (black boxes), plus the discordant gene models _102 (red box), _96 (green box) and _97 (blue box) for the assemblies iM_Zhong, Bg2024, i90 and i68. Colored tracks represent genes from different sources: Bg2024 (red), iBS90_Bu (green), iM_Bu (purple) and iM_Zhong (orange).

Case 8: Potential chimerism issues around Gene_Model_96 and Gene_Model_97 definition

While almost outside the study area, Gene models _96 and _97 require a special mention as they portray some limitations with our annotation approach: potential chimerism between annotations. Gene_Model_97 in it's more common configuration in this study shows 2 sets of genes that are connected by BgiMline_021656 (original annotation: iM_Bu). However, BS90_bu_et_al_qtl displays a different configuration where this gene is absent and the downstream portion of the gene model is fully independent and the upstream is merged with Gene_Model_96 by gene BgiMline_034962 (original annotation: iM_Bu) (see **Fig_Case10**). The original gene was also found on the iM_Zhong assembly but on the un-scaffolded contig JAKZJL020000167.1, making its actual location unclear.

All 1316R assemblies show the same organization as Bg2024 for this locus except for i68 where it is compromised by lost sequence (not shown).

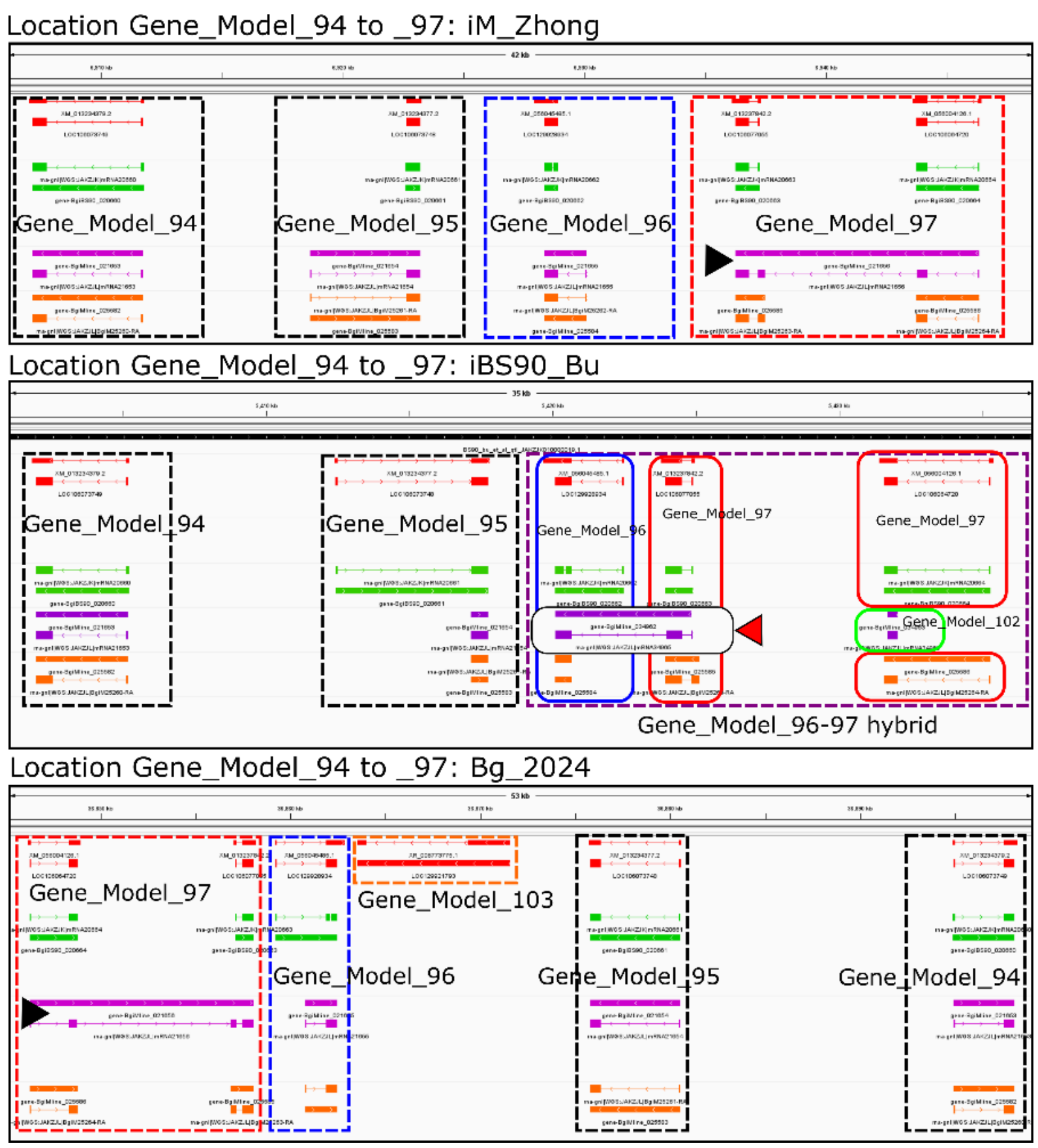

Fig_Case8: Sketches displaying the situation of Gene_Model_96 (blue box) and _97 (red box) on the 3 reference assemblies: iM_Zhong, iBS90_Bu and Bg2024. Other displayed gene models are the conserved Gene_Model_94 and _95 (black boxes), the Bg2024 + 1316R only Gene_Model_102 (green box), and _103 (orange box). Colored tracks represent genes from different sources: Bg2024 (red), iBS90_Bu (green), iM_Bu (purple) and iM_Zhong (orange). In most assemblies Gene_Model_97 shows the same configuration as in

iM_Zhong and Bg2024 where two sets of overlapping genes are bridged together by gene BgiMline_021656 (original annotation: iM_Bu) marked with a black pointer. On iBS90_Bu however the gene is missing and instead replaced with BgiMline_034962 (original annotation: iM_Bu) that combines it with Gene_Model_96, marked with a red pointer.

LG5R

Case 9: Example of gene model overlaps

The first 3 Gene models on LG5R show a significant overlap that is conserved in all assemblies except i46 where Gene_Model_1, defined by gene LOC129926517, was located on a different contig. When all gene models are present, Gene_model_2 is fully encoded within an intron of Gene_Model_1, while _1 and _3 overlap on their predicted 5' UTR regions (**Fig_Case12**). Several gene models on LG5R display the same sort of overlaps, for simplicity only this case is illustrated

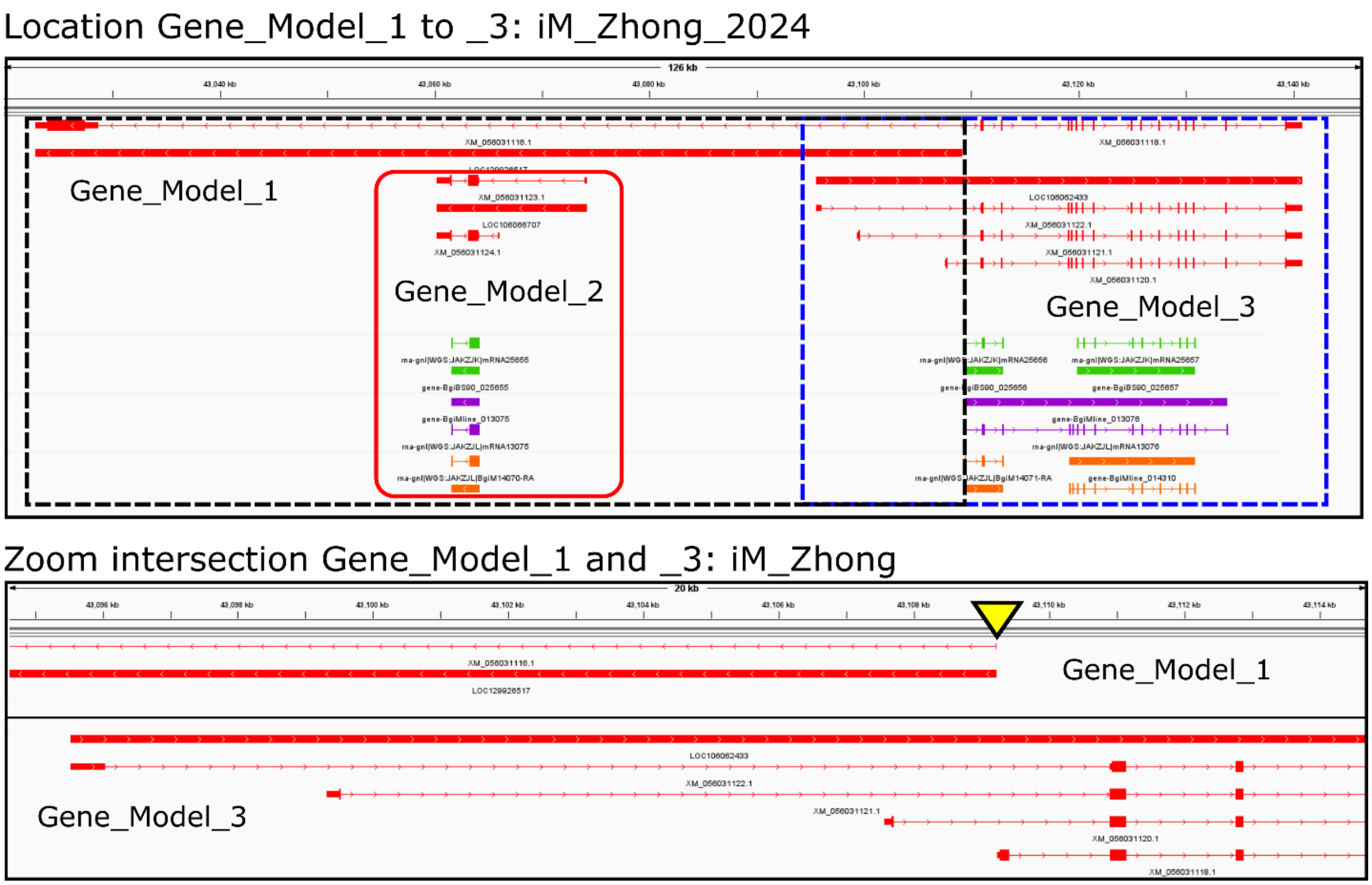

Fig_Case9: Sketches displaying the overlaps of the first 3 gene models of Region LG5R on the reference assembly iM_Zhong. Colored tracks represent genes from different sources: Bg2024 (red), iBS90_Bu (green), iM_Bu (purple) and iM_Zhong (orange). First is the overall representation of the area with Gene_Model_1 to _3 represented in black, red and blue respectively. Below are the gene tracks showing the overlap of the 5'UTRs of Gene_Model_1 and _3, the yellow arrow highlights the split. Note that both gene models are encoded in opposite strands.

Case 10: Gene models encompassing low complexity regions

Deletion present on Bg2024 causes Gene_Model_31 and _32 to overlap over low complexity regions. Leading to higher estimates of reported identity and coverage for the genes by Liftoff (**Fig_Case_10**).

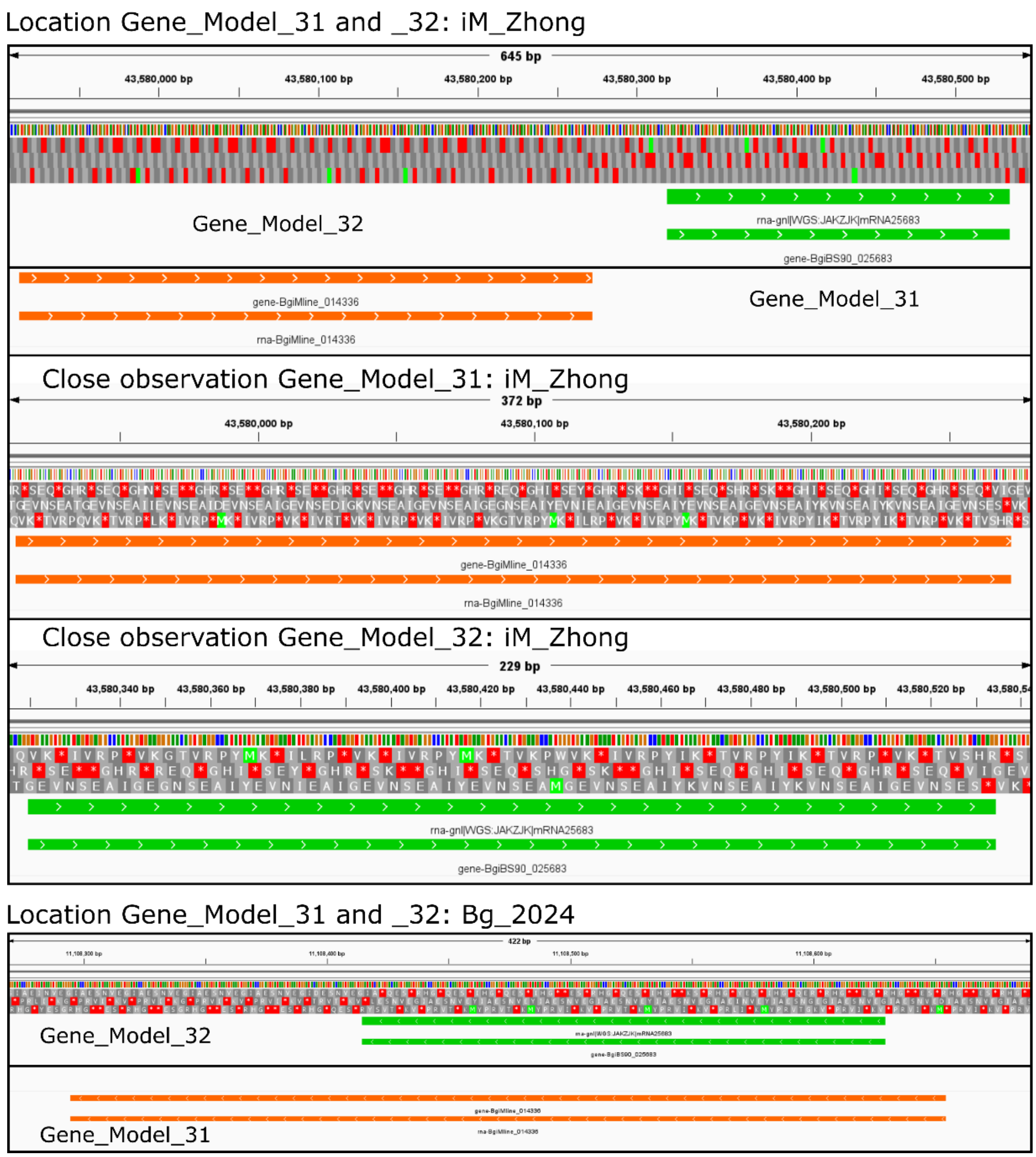

Fig_Case_10: Sketches displaying Gene_Model_31 (below) and _32 (above) for iM_Zhong, as representative of most assemblies, and Bg2024 where both gene models are compromised by a deletion. Leading to both gene models to be predicted as overlapping due to high repeated sequence. The first track shows the

predicted coding protein sequence for all 3 reading frames matching the direction of both genes. Colored tracks represent genes from different sources: iBS90_Bu (green) and iM_Zhong (orange).

Case 11: Ambiguous location of Gene_Model_230

Gene_Model_230 was found in 3 assemblies (Bg2024, i46 and i90) but with a relatively different location relative to Gene_Model_46 and _47. The most common configuration is shown in iM_Zhong where Gene_Model_47 is located within an intron of Gene_Model_46 and _230 is missing; while Bg2024 conserves this disposition but has Gene_Model_230 located between _45 and _46 (**Fig_Case_11a**). In contrast, on i46 Gene_Model_46 is modified (gene LOC106073718 is missing) and Gene_Model_230 overlaps extensively with its remaining gene signatures, while i90 is seemingly similar but the locus is disrupted by lost sequence in the genome assembly (**Fig_Case_11b**).

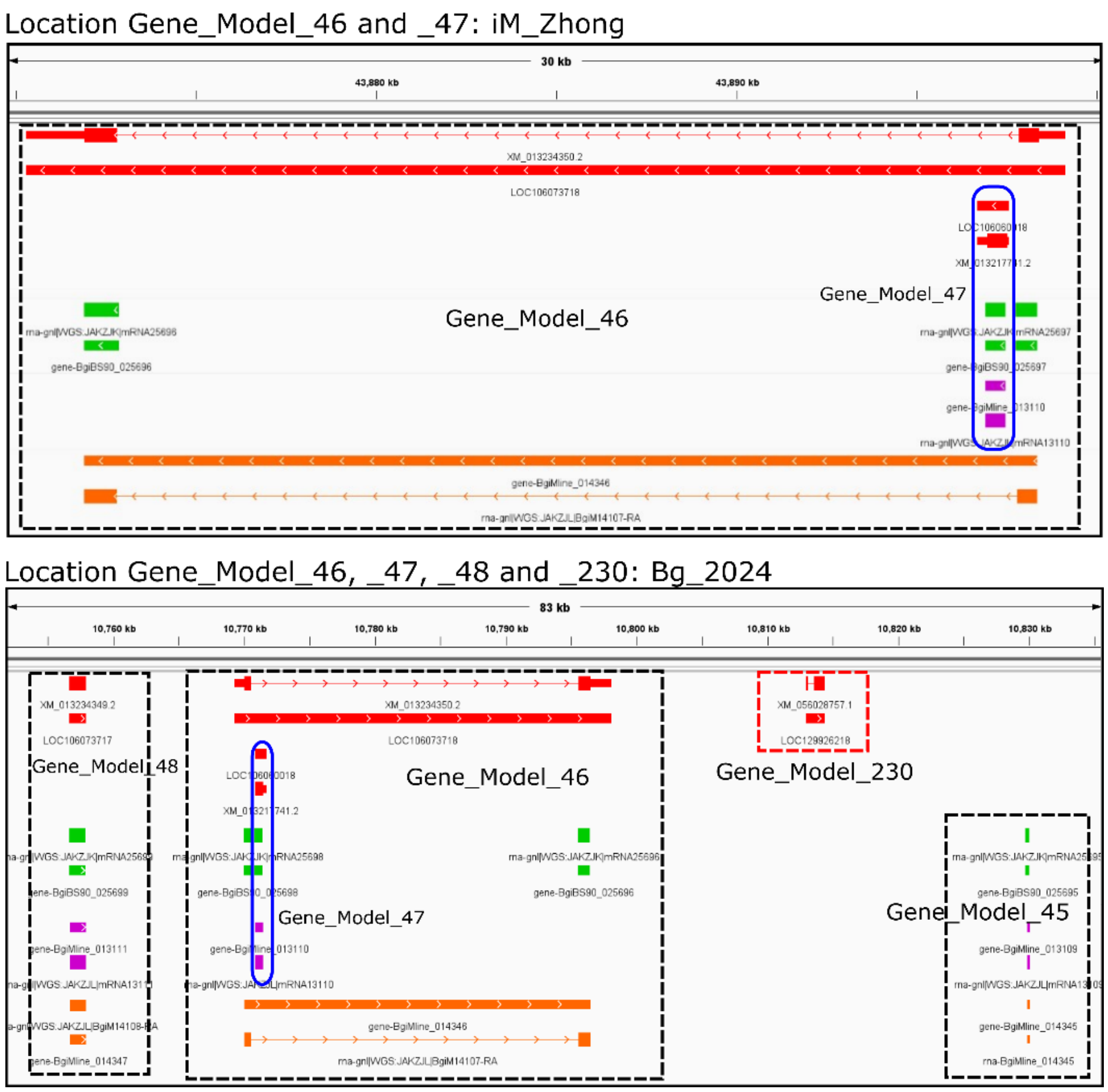

Fig_Case_11a: Sketches displaying Gene_Model_46 (black box), _47 (blue box) and _230 (red box) for the reference assemblies iM_Zhong and Bg2024. Colored tracks represent genes from different sources: Bg2024 (red), iBS90_Bu (green), iM_Bu (purple) and iM_Zhong (orange).

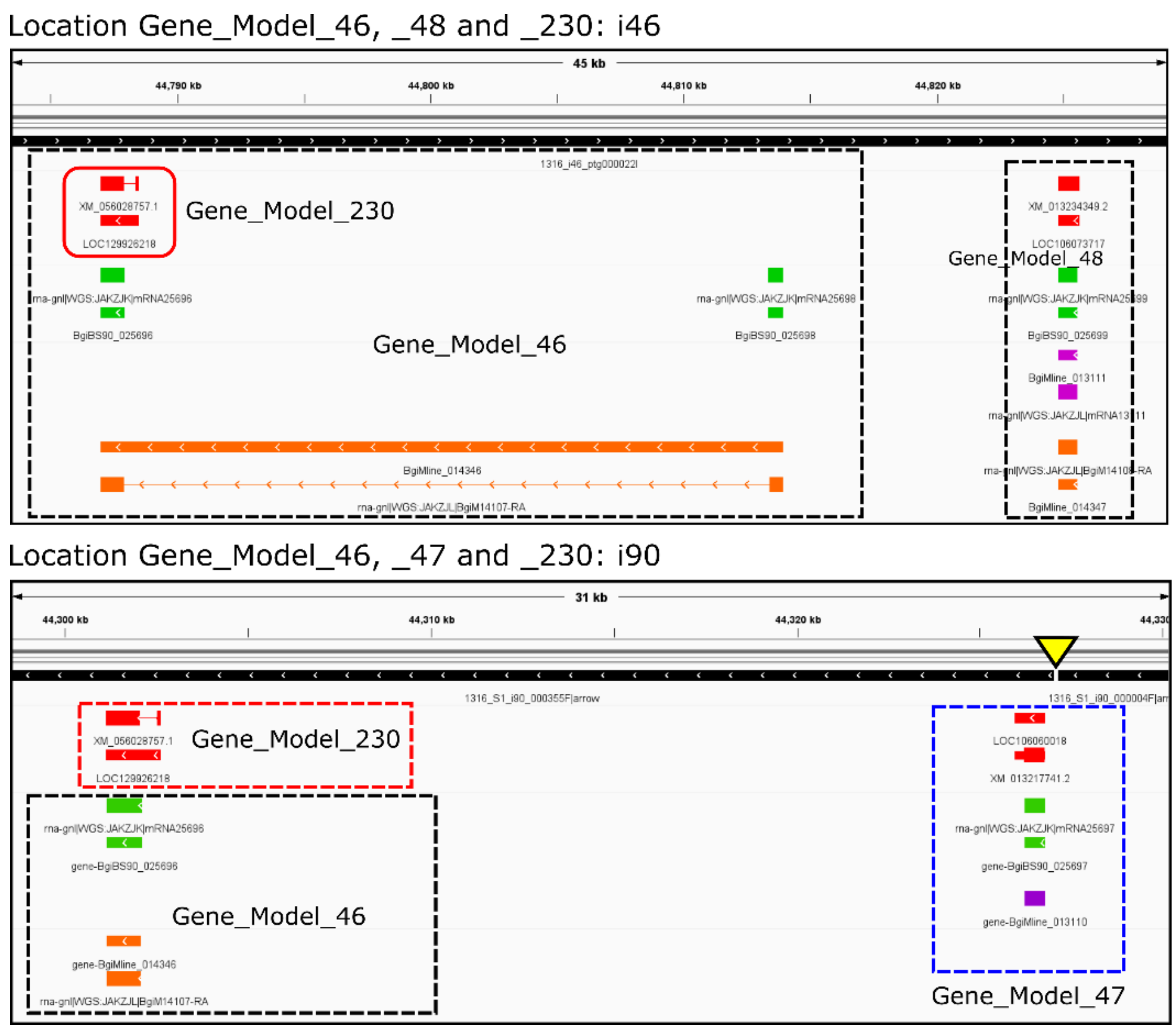

Fig_Case_11b: Sketches displaying Gene_Model_46 (black box), _47 (blue box) and _230 (red box) for the 1316R assemblies i46 and i90. Colored tracks represent genes from different sources: Bg2024 (red), iBS90_Bu (green), iM_Bu (purple) and iM_Zhong (orange). Yellow triangles highlight the original contig boundaries.

Case12: Ambiguous location of Gene_Model_229

Gene_Model_229 is found in the reference assembly Bg2024, the resistant 1316R for the region (i46 and i68) and the susceptible i90. In the latter however Gene_Model_229 is a small fragment and located in a different location (**Fig_Case12**).

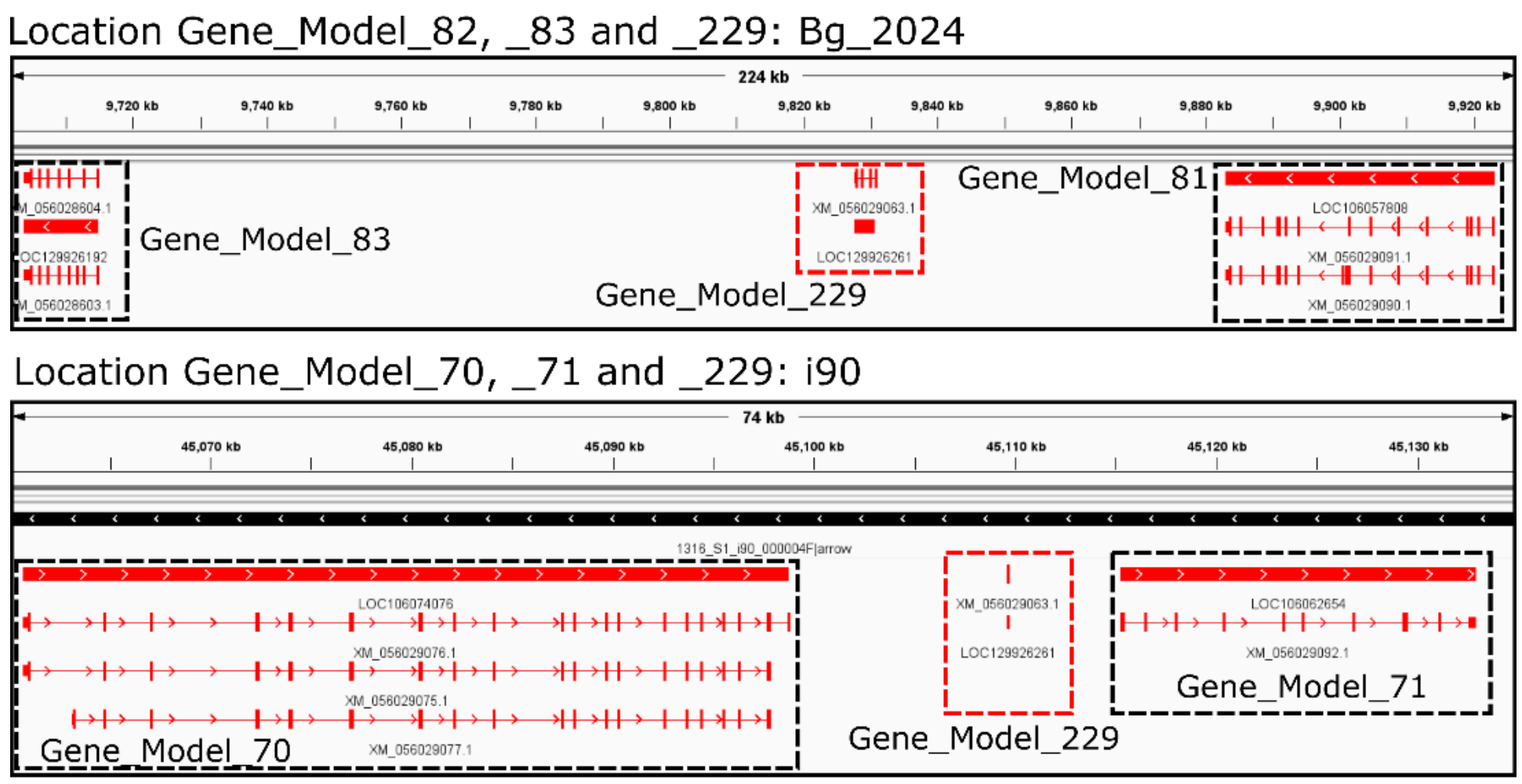

Fig_Case_12: Sketches displaying Gene_Model_231 (red box) and surrounding Gene Models (black box) for the reference assembly Bg2024 and the 1316R assembly i90. Red tracks show the genes assigned from the Bg2024, other sources are not shown for simplicity.

Case13: Frequent annotation transfer artifacts on several LG5R gene models

Reference genomes Bg2024 and iBS90_Bu show a complex cluster of several gene models around Gene_Model_93 that is absent on iM_Zhong (**Fig_Case13**). Upon inspection this seems to be a more extreme example of the C-lectin issues described before where annotation fragmentation combined with partial deletions causes Liftoff to assign the genes to the next best location. Typically, a paralog though in this case the main driver seems to repeat the abundance of Epidermal Growth Factor. In the name of simplicity, we will only illustrate this locus and refer to similar cases as “repeat driven anomalies”.

In addition to this case, Gene_Models _85 to _89 are excluded from the region map for this non-conserved location.

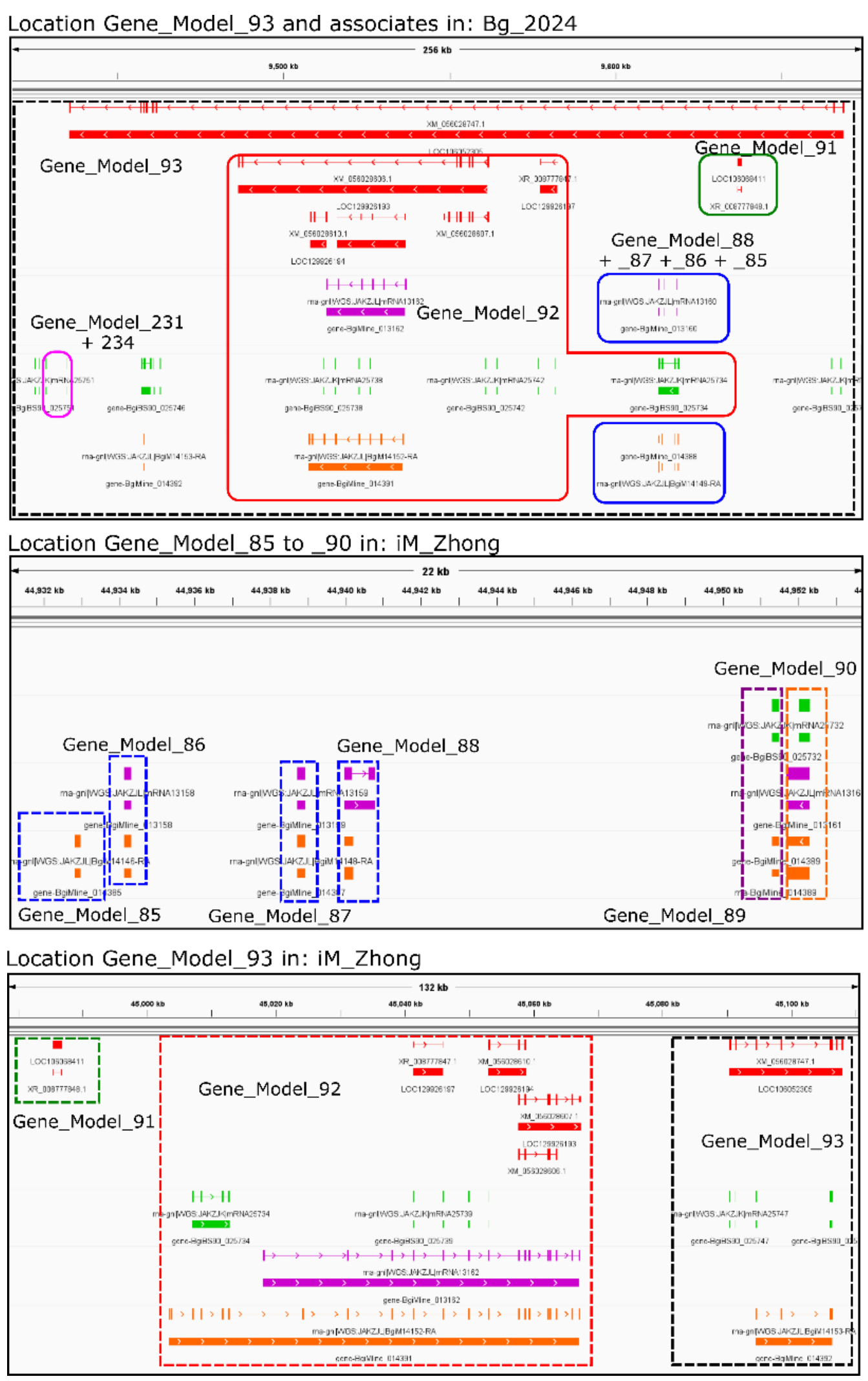

Fig_Case_13: Sketches displaying anomaly found on Gene_Model_93 (black box) on Bg2024 relative to iM_Zhong where 8 gene models get assigned as part of Gene_Model_93: Gene_Model_85 to _88 (blue box), _91 (dark green box), Gene_Model_92 (red box), _233 and _236 (pink box). Plus, the surrounding gene models _89 (purple box) and _90 (orange box). Colored tracks represent genes from different sources: Bg2024 (red), iBS90_Bu (green), iM_Bu (purple) and iM_Zhong (orange).

Case14: In most assemblies Gene_Model_107 is found overlapping the first exon of Gene_Model_108 but in the opposite strand (**Fig_Case14a**). Unlike other similar cases however, in iBS90_Bu the gene model is bigger, found between Gene_Model_105 and Gene_Model_106, and with small gene originally assigned to Gene_Model_108. Part of the explanation of this strange pattern is found directly upstream: Gene_Model_108 is present without obvious alterations, but some of their signatures are located outside it in an arrangement that suggests the existence of an extra gene that: a) was not fully recovered by any of the annotations and b) it is apparently missing on other assemblies. Assemblies i46 and i68 have a similar arrangement to iBS90_Bu, but Gene_Model_107 is reduced and there is no sign of the additional Gene_Model_108 copy **(Fig_Case14b)**.

Location Gene_Model_106 to _108 in: iM_Zhong

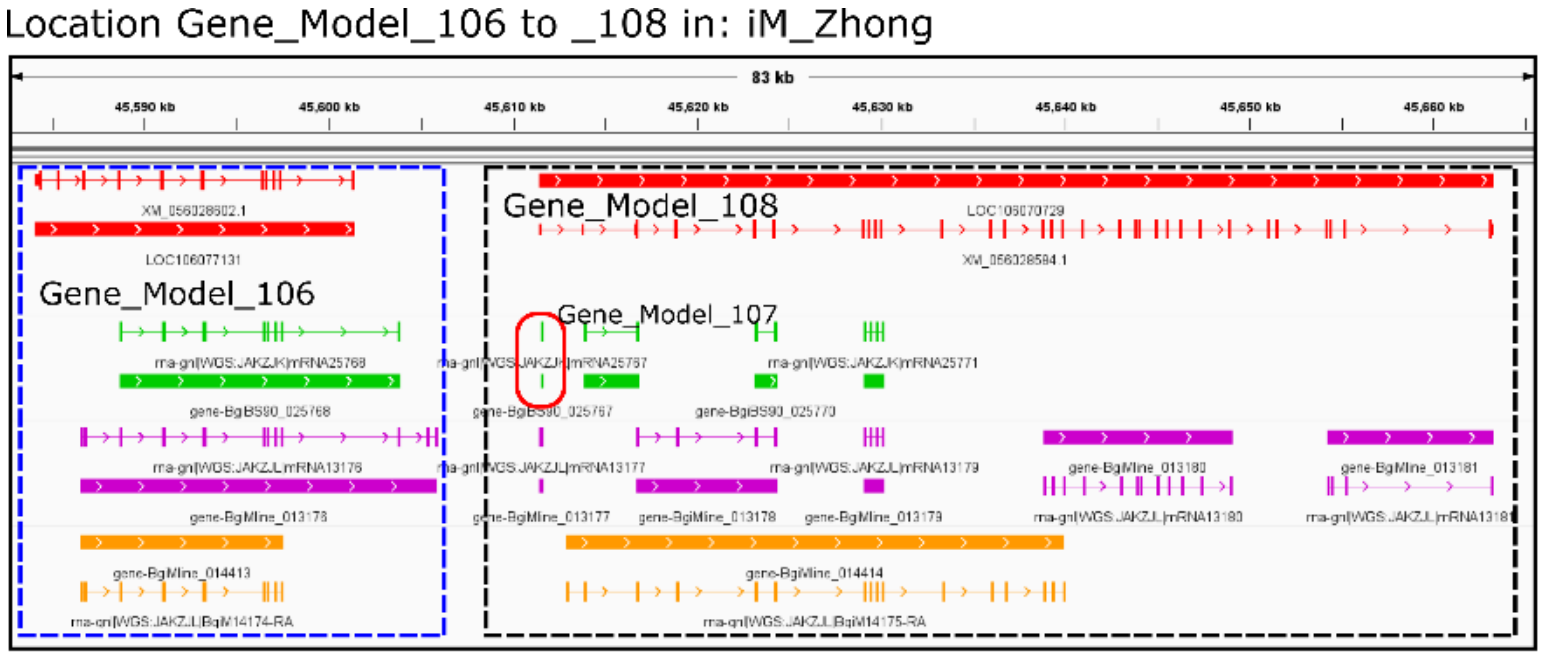

Location Gene_Model_106 to _108 in: iBS90_Bu

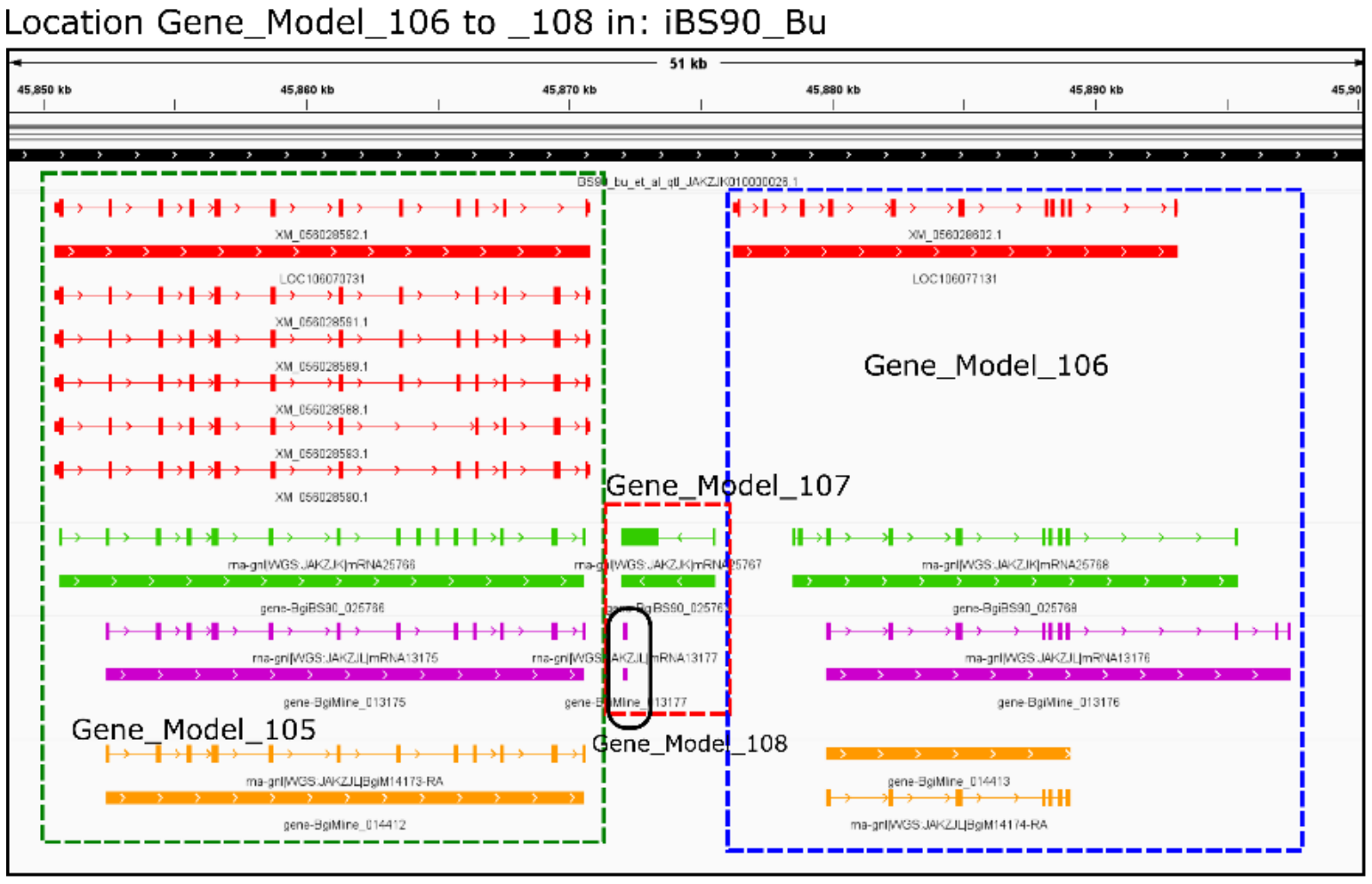

Location Gene_Model_106 to _108 in: iBS90_Bu

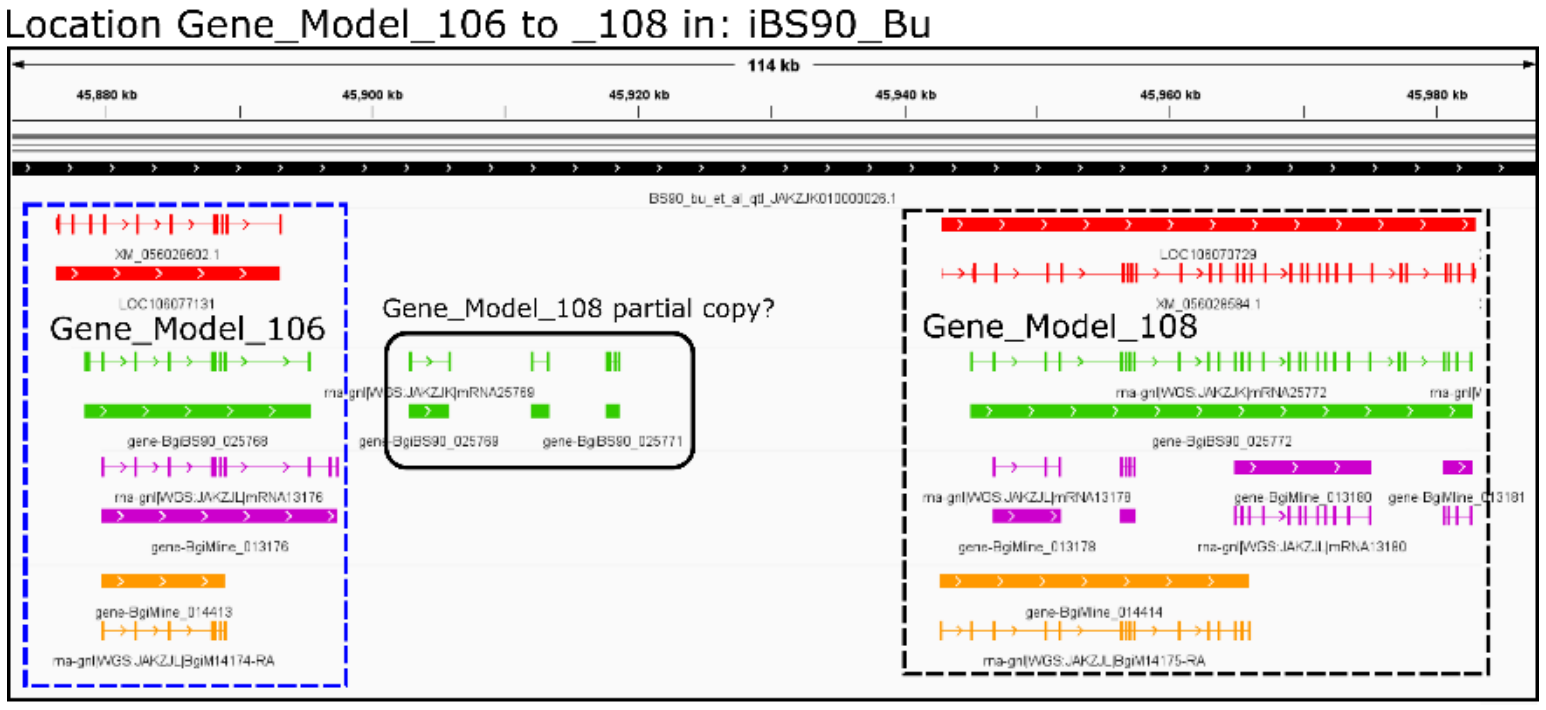

Fig_Case_14a: Sketches displaying the location of Gene_Model_107 (black box), plus the surrounding _105 (dark green box), _106 (blue box) and _108 (black box) on iBS90_Bu and iM_Zhong. Including a partial copy of Gene_Model_108 in iBS90_Bu. Colored tracks represent genes from different sources: Bg2024 (red), iBS90_Bu (green), iM_Bu (purple) and iM_Zhong (orange).

Location Gene_Model_106 to _108 in: i68

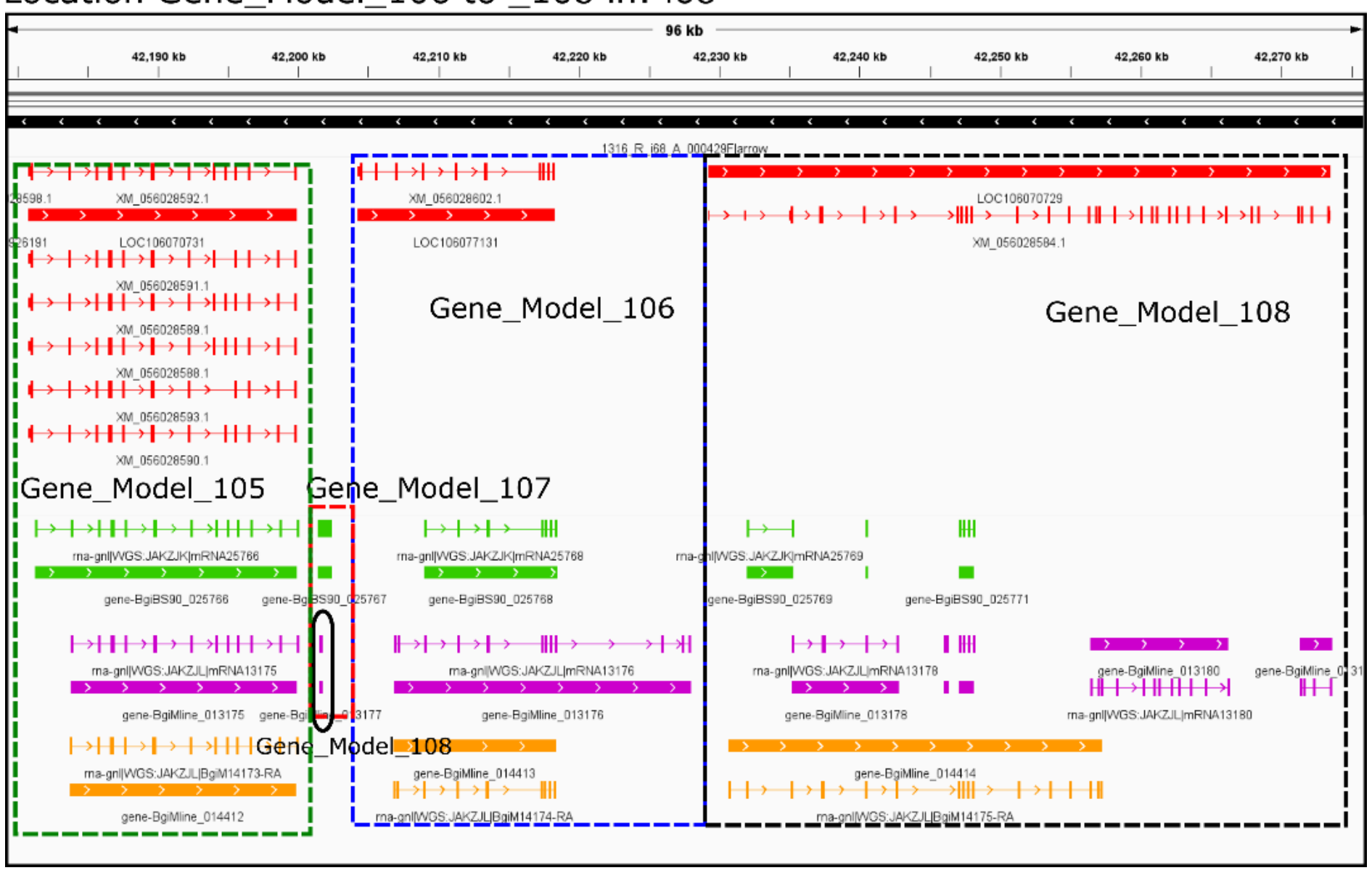

Fig_Case14b: Sketches displaying the location of Gene_Model_107 (black box), plus the surrounding _105 (dark green box), _106 (blue box) and _108 (black box) on iBS90_Bu and i68. Colored tracks represent genes from different sources: Bg2024 (red), iBS90_Bu (green), iM_Bu (purple) and iM_Zhong (orange).

Case15: Discrepancies on gene boundaries across assemblies

Gene boundaries and relationships between Gene_Model_124, _125, _126 and _226 show marked differences between assemblies the Bg2024 -like configuration (Bg2024, iBS90_Bu, 1316_S2_i171and i68) and the rest (**Fig_Case15**). Assembly i46 shows a similar configuration than Bg2024 but with Gene_Model_225 reduced relative to others.

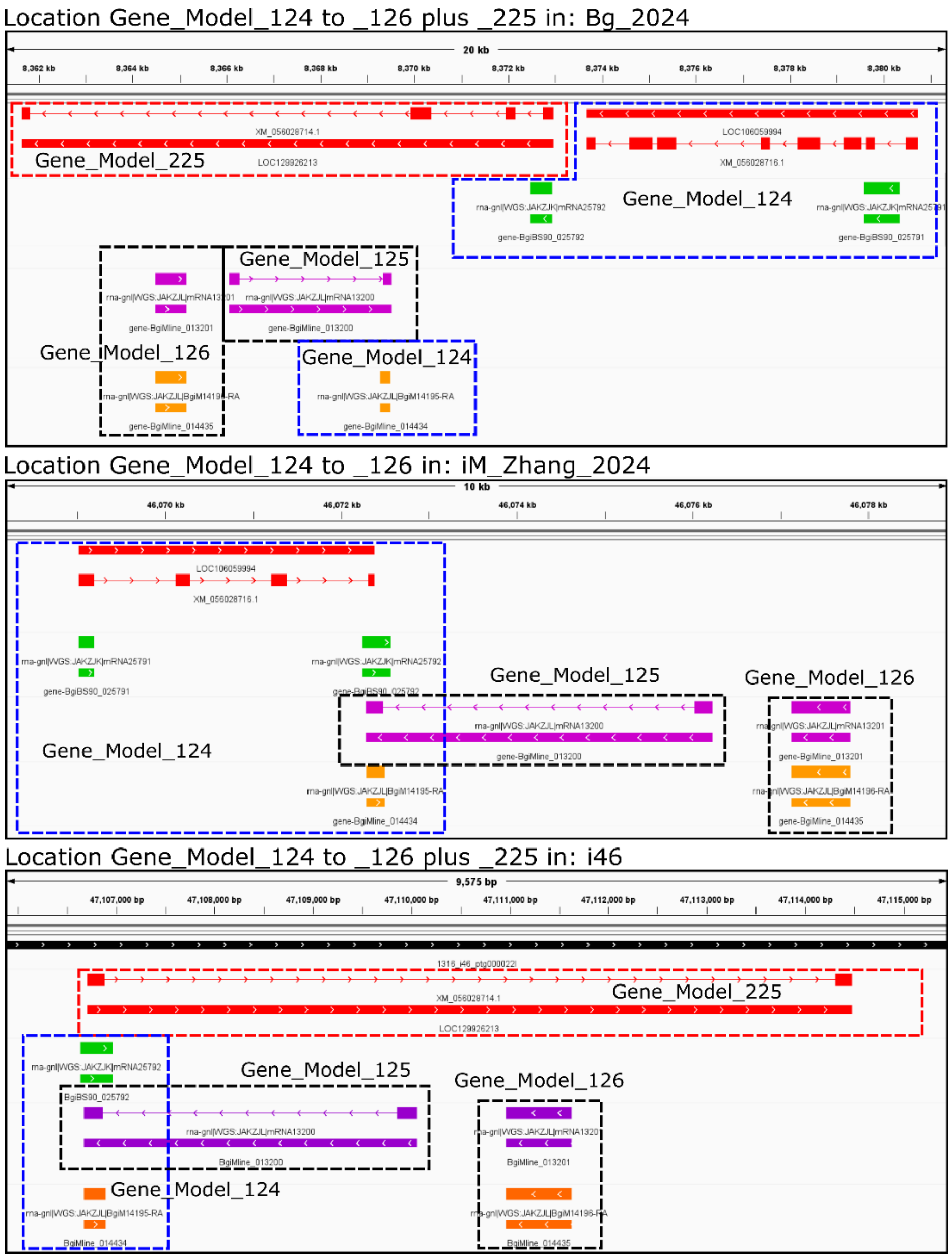

Fig_Case15: Sketches displaying the different associations of Gene_Model_124 (blue box), _226 (red box), relative to the more conserved _125 and _126 (black boxes) in Bg2024, iM_Zhong and i46. Colored tracks

represent genes from different sources: Bg2024 (red), iBS90_Bu (green), iM_Bu (purple) and iM_Zhong (orange).

Case16: Split gene model in two locations

On assemblies i46, i68 and i171 the Gene_Model_131 is split into two discrete loci, separated by ~100kb. The cause is unclear.

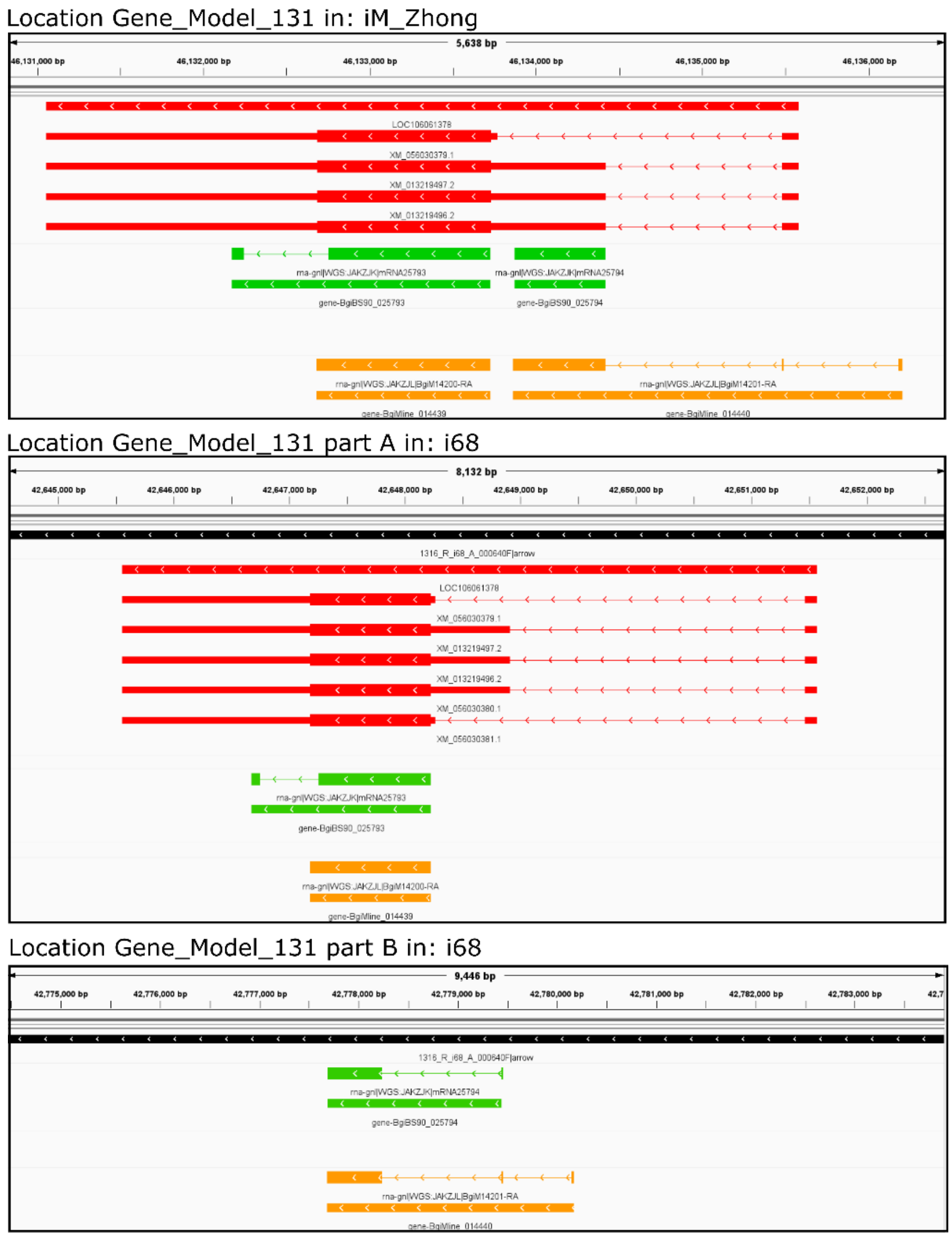

Fig_Case16: Sketches displaying the split location of Gene_Model_132 in iM_Zhong and i68. Colored tracks represent genes from different sources: Bg2024 (red), iBS90_Bu (green) and iM_Zhong (orange).

Case17: Pseudogenization of Gene_Model_107

Gene_Model_107 is a mitochondrial gene with an inconsistent annotation and dubius functionality in the *B. glabrata* assemblies (**Fig_Case17**). On most studied assemblies only a fragment of Gene_Model_107 is found as part of Gene_Model_108; while on iBS90_Bu (the source of the gene annotation) it is located between Gene_Model_106 and _107. The R 1316R assemblies i46 and i68 show Gene_Model_107 located in the same relative position as iBS90_Bu but in a disminished condition (Liftoff coverage: 64%)

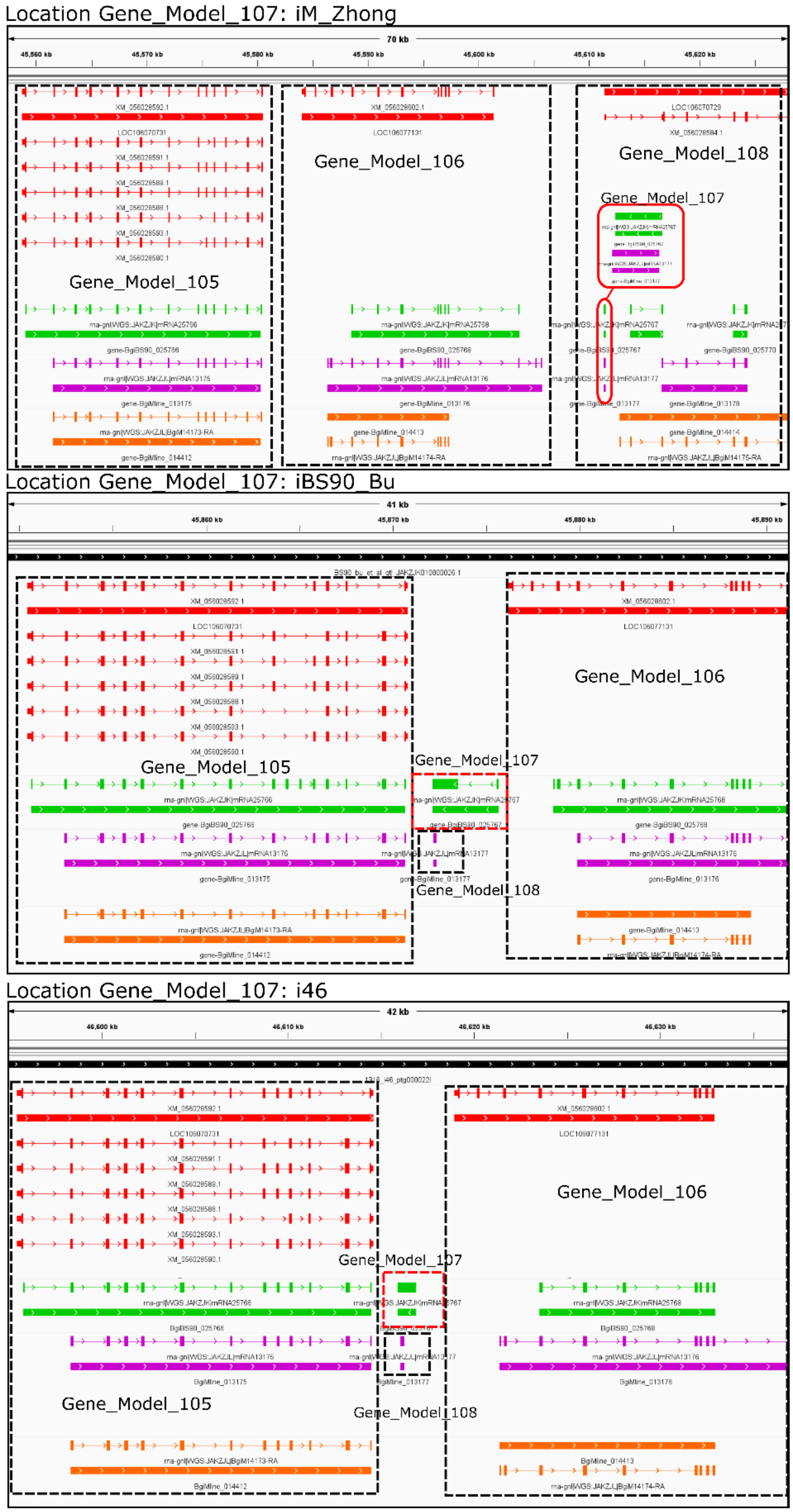

Fig_Case17: Sketches displaying the location of Gene_Model_107 (Red box) in iM_Zhong, Bg2024 and i68. Colored tracks represent genes from different sources: Bg2024 (red), iBS90_Bu (green) and iM_Zhong (orange).

Case 18: Inconsistent recovery of C:225 supports link between R 1316R and i90 for a small section outside the main Fst peak.

Gene models included in C:225 are recovered inconsistently between assemblies (**Fig_Case18**). On assemblies iM_Zhong, i36b and i90 the Gene_Model_225 is missing, instead Gene_Model_124 partially overlaps with _125 and _126 is nearby. Meanwhile on Bg2024 Gene_Model_124 is compromised and split and they are all encompassed by Gene_Model_225. Assemblies i68, i46 and i90 are similar to Bg2024 but with Gene_Model_225 reduced.

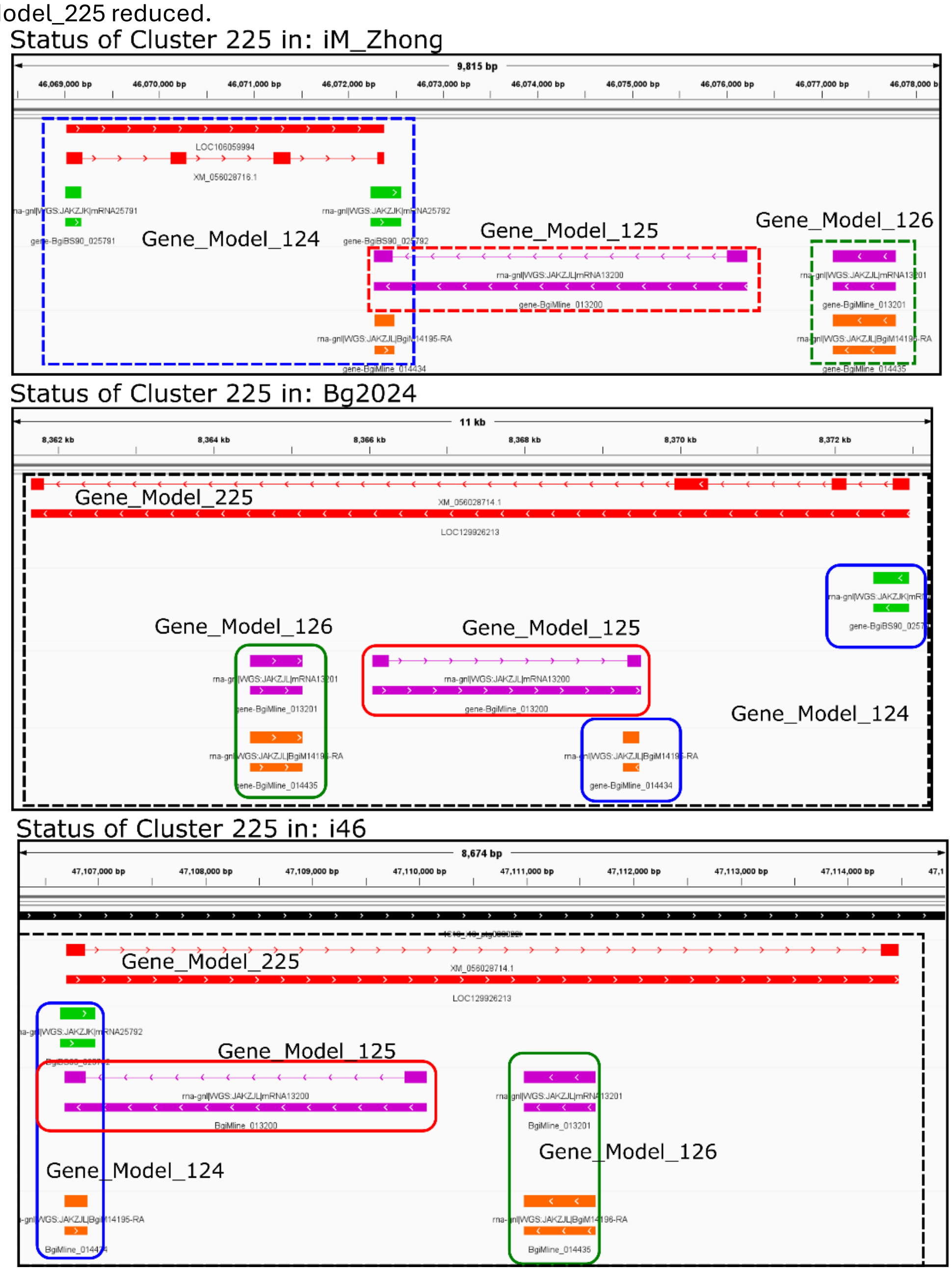

Fig_Case18: Sketches displaying the status of C:225 across assemblies (Black box) in iM_Zhong, Bg2024 and i46. Colored tracks represent genes from different sources: Bg2024 (red), iBS90_Bu (green) and iM_Zhong (orange). The nested gene models Gene_Model_124, _125 and _126 are shown with blue, red and green boxes respectively

Case 19: Cryptic duplication affecting Gene_Model_131

In most assemblies Gene_Model_131 is made of 2 sets of genes, linked by a small exon (**Fig_Case19**): First set LOC106061378 (Bg2024), BgiBS90_025793 (iBS90_Bu) and BgiMline_014439 (iM_Zhong) and Second set: BgiBS90_025794 (iBS90_Bu) and BgiMline_014440 (iM_Zhong). On assemblies i46, i68 and i171 they are split in two locations with no differences on their Liftoff Identity and Coverage. Likely as result of a duplication misleading the annotation transfer.

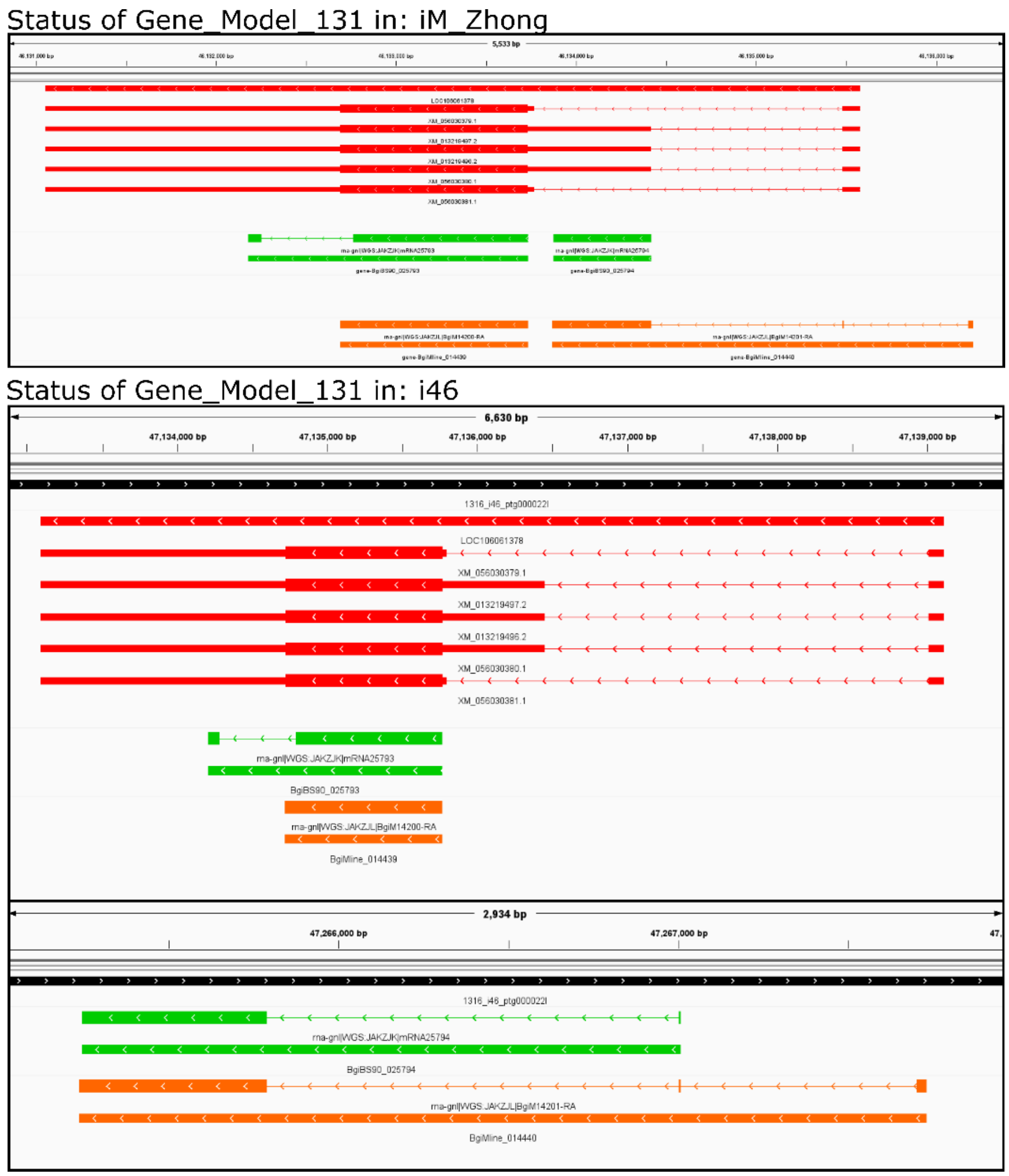

Fig_Case19: Sketches displaying transferred genes included in Gene_Model_131 in iM_Zhong and i46. Colored tracks represent genes from different sources: Bg2024 (red), iBS90_Bu (green), iM_Bu (purple) and iM_Zhong (orange).

Case20: Low complexity region makes gene transfers unreliable

The area between Gene_Models_138 to _142 in iM_Zhong is marked by a low complexity and proved hard to recover among assemblies. The most extreme example is the 1316R assembly i46 where multiple genes get re-arranged in order without clear changes to their sequence identity and coverage (Fig_Case20)

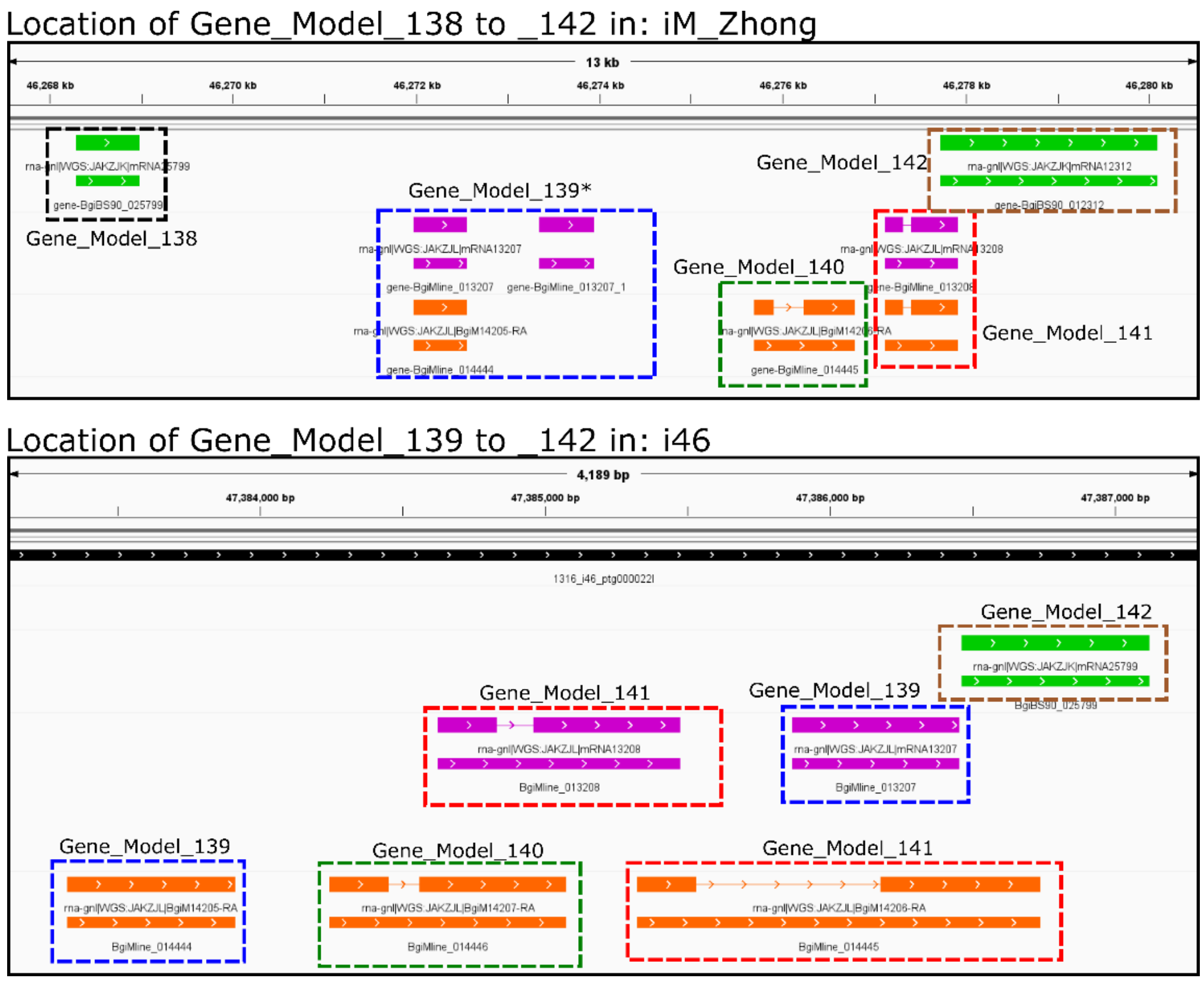

Fig_Case20: Sketches displaying transferred genes included in Gene_Model_138 to _142 in iM_Zhong and i46. Given the marked examples on gene model order and overlaps between one another, and lack of clear annotation of the genes within, it was not further analyzed. Colored tracks represent genes from different sources: Bg2024 (red), iBS90 (green), iM_Bu (purple) and iM_Zhong (orange).
