# Supplementary material for: The growing repertoire of putative pathogen interaction genes in the Biomphalaria glabrata vs Schistosoma mansoni system
Source: PLoS Negl Trop Dis. 2026 Oct 21;20(10):e0014803. doi: 10.1371/journal.pntd.0014803 (PMC13712769; doi:10.1371/journal.pntd.0014803)
Supplement: Supplementary file 9 [file pntd.0014803.s027.pdf]

Supplemental data 3:

Allelic variation between homologues on the S and R haplotypes

With the aim to reduce the gene candidate list behind the observed resistance/susceptibility against *S. mansoni*, we identified variants (SNPs and indels) located in homologous loci between the R and S haplotypes of the 1316R assemblies with likely function altering consequences: non-synonymous substitutions, premature stop codons, splice site disruptions. First the original contigs from the 1316R assemblies were mapped to the reference assembly that best matched one of the haplotypes for using Minimap2 v2.30-r1287 (Li, 2018). For LG5R this turned out to be iM_Zhong and Bg2024 for LG11L, in both cases representing the S haplotype. This choice allows us to avoid potential issues with the scaffolding outside the region of association that could hinder the results unexpectedly while minimizing inconsistencies with the annotation transfers on more diverse regions between haplotypes. Individual variants were then called using paftools.js call (included in Minimap2) and potential functional impacts measured predicted with SnpEff v5.4a (Cingolani et al., 2012) relative to the 4 annotation sources.

Variants were counted according to the following rules:

1) The gene had no warnings by snpEff.
2) There was no overlap between genes from the same annotation.
3) They were found in either:
    a. At least 2 susceptible/resistant 1316R assemblies with no intermix between them.
    b. All analyzed 1316R.
4) The highest impact prediction for a gene or isoform was representative for the variant.

Overlaps between Gene Models and other discrepancies with the gene transferred to the reference assembly from other references were issues manually reviewed and accounted for in the counts.

References

Cingolani, P., Platts, A., Wang, L. L., Coon, M., Nguyen, T., Wang, L., Land, S. J., Lu, X., & Ruden, D. M. (2012). A program for annotating and predicting the effects of single

nucleotide polymorphisms, SnpEff: SNPs in the genome of Drosophila melanogaster strain w1118; iso-2; iso-3. *Fly*, *6*(2), 80–92. https://doi.org/10.4161/fly.19695

Li, H. (2018). Minimap2: Pairwise alignment for nucleotide sequences. *Bioinformatics*. https://doi.org/10.1093/bioinformatics/bty191
